# Shaping and Stabilizing the Active Phase: The Role of Carbon Surface Defects in Carbon-Supported Co Fischer–Tropsch Synthesis Catalysts

**DOI:** 10.1021/acscatal.5c06572

**Published:** 2025-12-19

**Authors:** Felix Herold, Mei Ju A. Goemans, Pierre Cautaerts, Bastian J. M. Etzold, Magnus Rønning

**Affiliations:** † 8018 Norwegian University of Science and Technology, Department of Chemical Engineering, Trondheim 7491, Norway; ‡ Institute for Power-To-X Technologies, 9171 Friedrich-Alexander-Universität Erlangen-Nürnberg, Fürth 90762, Germany

**Keywords:** Fischer−Tropsch synthesis, carbon support, carbon surface defects, metal−support interactions, sintering, metal anchoring

## Abstract

Carbon supports offer a promising alternative to conventional oxide supports for cobalt-based Fischer–Tropsch synthesis (FTS) catalysts. However, unlike well-studied oxide systems (e.g., Co/Al_2_O_3_, Co/TiO_2_), the fundamental interactions between cobalt nanoparticles (Co NP’s) and unfunctionalized carbon surfaces remain poorly understood, largely due to the structural and chemical diversity of carbon materials. Establishing a universal “baseline” interaction for Co/C interfaces has therefore remained elusive. In this work, we investigated Co anchoring mechanisms on two carbon black model supports that differ by a factor of 20 in surface defect (chemisorption) site density but exhibit otherwise similar properties. On this basis, Co-based catalysts were synthesized using size-controlled colloidal Co nanoparticles and conventional incipient wetness impregnation. Employing high-resolution SEM and HAADF STEM imaging, we could show that Co NP sintering occurs predominantly via nanoparticle migration and coalescence during catalyst reduction, with negligible additional growth under FTS conditions—implying that Co NP anchoring is established in the reduction step. Combined in situ XANES/XRD experiments during reduction, coupled with off-gas analysis by online mass spectrometry, showed that Co phase transformations coincided with significant CO_2_ and CH_4_ evolution. This was attributed to carbothermal reduction and carbon hydrogasification at the Co/C interface, which appeared to correlate with the density of carbon surface defect (chemisorption) sites. We hypothesize that carbon gasification at the Co/C interface is directly linked to the immobilization of Co NP, as it generates highly reactive “dangling bonds” at the Co/C interface, which act as anchoring points. Overall, the defect-rich carbon support stabilized Co nanoparticles more effectively than its defect-poor counterpart, resulting in most cases in higher FTS activity. Our results imply carbon gasification-mediated anchoring as a “baseline” interaction for Co/C catalysts and suggest that the chemisorption site density—as measurable by simple TPD or TPO—can serve as a practical descriptor for designing more stable carbon-supported FTS catalysts.

## Graphical abstract

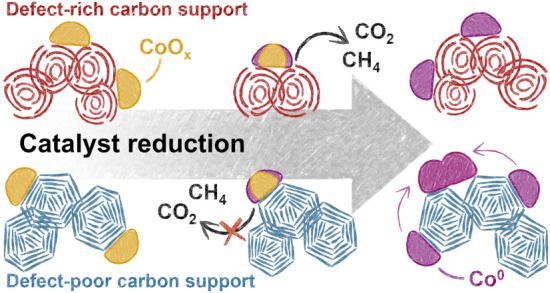

## 1 Introduction

The Fischer–Tropsch Synthesis (FTS) is a key process for producing sustainable aviation fuels^1^ and chemical building blocks,^2–4^ converting CO and H_2_ (syngas) sourced from renewable carbon and green hydrogen into hydrocarbons. A frequently employed catalyst system for FTS is composed of cobalt nanoparticles (Co NP’s) dispersed on a suitable support, which ideally stabilizes the active Co^0^ phase while providing high surface areas and anchoring points for the Co NP’s.^5^ Catalysts developed for industrial use often employ strongly interacting porous oxides like γ-Al_2_O_3_ and TiO_2_ as supports,^5,6^ which anchor Co NPs effectively and limit sintering but suffer from low Co reducibility and from the formation of nonregenerable metal–support compounds (e.g., Co_*x*_Al_*y*_O_*z*_, Co_*x*_Ti_*y*_O_*z*_).^7–11^ A contrasting alternative are weakly interacting carbon supports, sometimes exaggeratedly described as “inert”,^12^ which offer high cobalt reducibility and avoid the formation of cobalt-support compounds.^13^ However, due to the lack of strong metal/support interactions, carbon supports often fail to adequately anchor cobalt nanoparticles, leading to catalyst deactivation through increased cobalt sintering.

For strongly interacting catalyst supports such as γ-Al_2_O_3_ and TiO_2_, metal–support interactions have been widely studied, establishing an understanding of the evolution of the Co/Al_2_O_3_ ^7,10,14–19^ and Co/TiO_2_ ^10,11,20–26^ interfaces during catalyst synthesis, activation, and FTS. This understanding includes the effects of catalyst precursors,^17,24^ activation/reaction conditions (for example H_2_:H_2_O ratios),^7,10,11,15,16,19,21–23,25^ and Co nanoparticle size distribution,^7,15,19,23–25^ thus clarifying how metal–support interactions govern catalyst activity and stability.

While it has been recognized that carbon supports are certainly not “inert”,^27^ the understanding of cobalt/carbon interactions remains much less developed compared to Co/Al_2_O_3_ and Co/TiO_2_ systems. This gap largely stems from the fact that, unlike Al_2_O_3_ and TiO_2_—where a narrow range of materials dominates literature (e.g., “Puralox” from Sasol for γ-Al_2_O_3_;^7,10,16,17,28–30^ “P25” from Evonik for TiO_2_,^10,21,22,24,26^ )—carbon supports encompass a wide variety of materials with diverse nanostructures, textures, and surface functionalities. As a result, the structure of carbon support surfaces—and thus the properties of Co/C interfaces—varies significantly, particularly since most studies focus on functionalizing carbon supports with electronegative heteroatoms (e.g., O,^31–41^ N,^42–53^ S,^43^ P^43^), which are usually present in form of complex ensembles of various chemically unique surface species. In consequence, a universally valid “baseline” interaction between Co NP’s and unmodified carbon surfaces, as well as the structural features and mechanisms governing this interaction, remain largely unknown.

Considering the interaction between metal NP’s and unmodified carbon surfaces, there is growing evidence that carbon surface defects play a key role, acting as nucleation sites during catalyst synthesis and anchoring sites for metal nanoparticles.^27,54–57^ In this context, the term “defect” broadly refers to deviations from the ideal trigonal planar configuration of sp^2^-hybridized carbon in terms of bond angle and/or binding configuration, including structural features such as surface curvature, nonhexagonal ring structures (e.g., 5-, 7-, or 8-membered rings), sp^3^- or sp-hybridized carbon atoms, carbenes, or unpaired electrons.^27,57–59^ Naturally, these defects exhibit a wide range of chemical reactivities depending on their structure, influencing their ability to adsorb and interact with metal species.^27,60^ Due to the low concentration of such defect sites relative to regular sp^2^-hybridized carbon atoms in most carbon materials and the high reactivity of some species like carbenes or radicals—which are readily passivated by O_2_, H_2_O or CO_2_ upon air exposure^61,62^ —their experimental characterization remains highly challenging.^57,63–65^ As a result, the investigation of metal–carbon defect interactions remains largely theoretical.^63,66–71^ However, although the selective introduction and characterization of individual carbon defects remains a challenge, their overall concentration and collective reactivity may be probed experimentally through their behavior as chemisorption sites for heteroatoms (e.g., H, O), providing a pathway to experimentally investigate defect-related metal–support interactions.^64,65^

In this study, we investigated the role of carbon surface defects in carbon-supported Co-based Fischer–Tropsch synthesis catalysts, aiming to explore a more universal “baseline” interaction between cobalt nanoparticles and the “plain” carbon surface. To this end, we synthesized two carbon black-based model supports: one with a high and one with a low surface defect density, while maintaining similar morphology and texture. Co-based FTS catalysts were then prepared via incipient wetness impregnation and deposition of colloidal Co NP’s, enabling an assessment of the influence of carbon surface defects across commonly used synthesis methods. The use of colloidal Co NP’s ensured well-defined cobalt nanoparticle size distributions and morphologies, allowing insights into Co particle growth mechanisms. By combining comprehensive (*in situ*) characterization with FTS performance data, we developed a hypothesis for the anchoring of Co nanoparticles on carbon surfaces and identified structural features and descriptors for carbon supports capable of effectively stabilizing cobalt nanoparticles during FTS.

## 2 Materials and Methods

### 2.1 Materials

All gases were purchased from Linde AG. Carbon black (Printex 60) was provided by Orion Engineered Carbons. Nitric acid (65 wt %), hydrochloric acid (36 wt %), oleic acid (90%, technical grade), 1,2-dichlorobenzene (99%, anhydrous), cobalt­(II) nitrate hexahydrate (≥98%) and dicobalt octacarbonyl (90%) were obtained from Sigma-Aldrich. *n*-Hexane, ethanol, and toluene were acquired from Merck, while CaC_2_O_4_·H_2_O, isopropanol, acetone, and 1-octadecene (90%) were sourced from Fisher Scientific. CoO and Co_3_O_4_ powder standards were purchased from Alfa Aesar. It should be noted that all volumetric flow rates reported in the following are specified for standard conditions (STP).

### 2.2 Acid Washing of Carbon Black

In a round-bottom flask fitted with a reflux condenser, 10 g of carbon black were suspended in 150 mL of HCl (36 wt %) by magnetic stirring and heated to 90 °C for 24 h. After cooling, the carbon black was collected by filtration, washed with deionized water until the effluent reached neutral pH and dried in air at 100 °C for 24 h.

### 2.3 Graphitization of Carbon Black

5 g of the acid-washed carbon black was heated in a graphite vacuum furnace (CAF 140/140–2000G, MUT Advanced Heating) at a total pressure of 15 mbar and a flow of 2 L min^–1^ Ar to 1900 °C for 10 min. The heating rate was 20 °C min^–1^ from 20 – 1200 °C, 15 °C min^–1^ from 1200 to 1500 °C, 10 °C min^–1^ from 1500 to 1700 °C and finally 5 °C min^–1^ from 1700 to 1900 °C.

### 2.4 High-Temperature Hydrogen Treatment

5 g of the graphitized and nongraphitized (after acid washing) carbon black samples were heated in a vertical tube furnace in a flow of 100 mL min^–1^ H_2_ at a rate of 10 °C min^–1^ to 950 °C, which was kept constant for 0.5 h before allowing it to cool. After this hydrogen treatment, the nongraphitized carbon black is designated as “CB”, while the graphitized carbon black sample is denoted as “CB-1900”.

### 2.5 Colloidal Nanoparticle Synthesis and Loading

The synthesis and loading of colloidal Co nanoparticles followed the procedures described by van Deelen et al.^33,72^ A 100 mL three-neck flask was fitted with a condenser, two septa and a magnetic stirrer, was connected to a Schlenk line in order to degass 73.5 μL of oleic acid under vacuum at 100 °C while stirring at 150 rpm. After degassing, the system was flushed with nitrogen (N_2_), and 7.5 mL of dry 1,2-dichlorobenzene were introduced before heating the mixture to 174 °C. Inside a glovebox, 270 mg of dicobalt octacarbonyl was dissolved in 1.5 mL of 1,2-dichlorobenzene by stirring at room temperature and quickly injected into previously prepared reaction mixture at 174 °C while stirring at 750 rpm. After maintaining the temperature at 174 °C for 20 min, the Co nanoparticle (Co NP) dispersion was quenched in a water bath. One septum was then removed, and the N_2_ flow was stopped, allowing the Co NPs to be gradually exposed to air over the course of 1 h while stirring at 650 rpm. The Co NP dispersion was subsequently transferred to a 50 mL centrifuge tube, and isopropanol was added to bring the total volume to 45 mL. The dispersion was centrifuged at 2200*g* for 20 min, the supernatant was discarded, and the Co NPs were redispersed in 1 mL of *n*-hexane, followed by the addition of isopropanol to reach a total volume of 40 mL. This washing cycle was repeated three times, and the Co NPs were finally redispersed in 2 mL of toluene and stored in a glass vial. After quality control using scanning transmission electron microscopy (STEM), several batches of Co NP dispersions were combined, ensuring that all catalysts in this study were loaded with the same composition of mixed Co NP batches.

The loading of Co NPs onto the carbon black supports was performed via wet impregnation. A 100 mL three-neck flask was fitted with a condenser, two septa, and a magnetic stirrer in order to disperse 750 mg of carbon black (CB) in 15 mL of 1-octadecene by stirring at 400 rpm for 15 min. Subsequently, 2.4 mL of the Co NP dispersion in toluene was added, and the suspension was degassed under vacuum at 100 °C for 30 min. The system was then flushed with N_2_, heated to 200 °C for 30 min, before being allowed to cool. The resulting dispersion was transferred to a 50 mL centrifuge tube, diluted by 20 mL acetone, and centrifuged at 2500*g* for 5 min. After removing the supernatant by decantation, the catalyst was redispersed in 2 mL of *n*-hexane, diluted by 6 mL of acetone before centrifuging again. After repeating this washing cycle six times, the Co/CB catalysts were dried overnight under vacuum at 60 °C. Co/CB catalysts prepared by loading of colloidal Co NP’s are denoted by the ending “–NP”.

### 2.6 Incipient Wetness Impregnation

Incipient wetness impregnation (IWI) was carried out using a procedure by Eschemann et al.,^38^ using a 1.5 M solution of Co­(NO_3_)_2_·6H_2_O in EtOH. Impregnation was conducted by dropwise adding the solution to a sample of CB support while continuously mixing, using a volume of solution corresponding to the pore volume of the support sample. After impregnation, the sample was dried at room temperature for 2 h, before being transferred to a drying oven to dry at 60 °C in air overnight. Subsequently, the samples were calcined in a vertical tube furnace by heating them at a rate of 2 °C min^–1^ to 250 °C in a 100 mL min^–1^ flow of Ar for 4 h. Co/CB catalysts prepared by incipient wetness impregnation are designated by the ending “–IWI”.

### 2.7 Ex Situ Characterization

Nitrogen physisorption measurements were carried out using a Micromeritics Tristar 3020 analyzer at −196 °C following overnight degassing of the samples at 200 °C and 0.01 Torr. Specific surface areas were determined using the BET method. Raman spectroscopy was performed with a Horiba Jobin Yvon LabRAM HR800 Raman microscope using a HeNe laser (λ = 633 nm). For each sample, at least five spectra were collected from different locations and analyzed using a curve-fitting procedure as proposed by Mallet-Ladeira et al.^73^ Detailed information regarding the determination of carbon *d*-spacing and crystallite size can be found in the Supporting Information. For X-ray photoelectron spectroscopy (XPS), a Kratos Analytical Axis Ultra DLD spectrometer was employed, using monochromatic Al Kα irradiation (1486.6 eV) and operating the anode at 10 kV with an aperture of 700 × 300 μm. Information concerning the deconvolution of X-ray photoelectron spectra can be found in the Supporting Information. Temperature-programmed desorption (TPD) was conducted using a Netzsch STA449 F3 thermogravimetric balance coupled to an online mass spectrometer (Netzsch Aeolos) calibrated using CaC_2_O_4_·H_2_O as a standard. Measurements were conducted in a flow of 50 mL min^–1^ of Ar, heating the sample to 1400 °C at a rate of 10 °C min^–1^. To assess the oxygen chemisorption capacity of the supports, O_2_ was preadsorbed at 300 °C for 1 h in a flow of 50 mL min^–1^ of synthetic air, before cooling to 50 °C and starting the TPD run in 50 mL min^–1^ Ar, with a heating rate of 10 °C min^–1^ to 1400 °C. Temperature-programmed oxidation (TPO) was carried out in the same instrument, heating the carbon sample at 5 °C min^–1^ in a flow of 100 mL min^–1^ synthetic air to 1000 °C. H_2_O and EtOH sorption isotherms were measured at 25 °C, using a gravimetric sorption analyzer DVS Resolution by Surface Measurement Systems and N_2_ as carrier gas. Cobalt loading of Co/CB catalysts was determined by microwave digestion in *aqua regia* (Berghof SpeedWave XPERT) followed by analysis using microwave plasma-atomic emission spectroscopy (MP-AES; Agilent 4210). Temperature-programmed reduction (TPR) was performed using an Altamira Benchcat analyzer. Approximately 25 mg of Co/CB catalyst was heated in a flow of 20 mL min^–1^ 7 vol % H_2_ in Ar at 5 °C min^–1^ to 800 °C, while monitoring the off-gas H_2_ concentration using a thermal conductivity detector (TCD). Scanning transmission electron microscopy (STEM) and high-resolution scanning electron microscopy (SEM) were conducted on a Hitachi SU9000 microscope operating at 30 kV. For STEM/SEM, samples were ultrasonically dispersed in *n*-hexane and drop-cast onto holey carbon-coated copper grids. Spent catalysts were washed thoroughly with *n*-hexane prior to redispersion and grid deposition. Cobalt particle size distributions were determined using ImageJ by analyzing 300–500 particles. Cobalt particle diameters were corrected for 3 nm cobalt oxide surface layer, using the relation *d*(Co^0^) = 0.75 *d*(CoO_*x*_) as proposed by Eschemann et al.^38^

### 2.8 In Situ Characterization

Combined *in situ* X-ray absorption spectroscopy (XAS) and X-ray powder diffraction (XRD) experiments were performed at beamline BM31 of the Swiss-Norwegian Beamlines (SNBL) at the European Synchrotron Radiation Facility (ESRF) in Grenoble, France. Tubular quartz capillary reactors (1.5 mm inner diameter, 0.02 mm wall thickness) were loaded with 5–10 mg of Co/CB catalyst to form a 1 cm bed, fixed in place with quartz wool plugs at both ends. The reactor was placed inside a custom-designed resistance heater^74^ for temperature control and connected to a gas distribution system. Online off-gas analysis was carried out via mass spectrometry (Pfeiffer Omnistar). XRD data were collected using a Pilatus3 2 M detector (Dectris) at a wavelength of λ = 0.24486 Å. A NIST 660a LaB_6_ standard was used to account for instrumental broadening, perform wavelength calibration, and correct detector distance. XAS was conducted at the Co K-edge (7709 eV) in transmission mode. Reduction experiments were carried out in a flow of 0.5 mL min^–1^ of 25 vol % H_2_ in He, selected to match the space velocity used for catalyst pretreatment prior to FTS. Samples were heated from 50 to 350 °C at 3 °C min^–1^, then held at 350 °C for 1–6 h, until no further changes were observed in the XANES spectra. Throughout the reduction, XANES spectra and XRD patterns were recorded. EXAFS and XRD data were additionally collected at 50 °C before and after reduction, without intermediate exposure to air. EXAFS standards included a Co^0^ hcp foil, CoO, and Co_3_O_4_ powders, all measured ex situ in transmission mode. Additional *ex situ* EXAFS data were collected for Co nanoparticles dispersed in toluene. *Ex situ* XRD reference patterns were recorded for the plain carbon supports (CB and CB-1900). XANES and EXAFS data processing was carried out using the Athena and Artemis programs from the Demeter software suite.^75^ XANES spectra were normalized and analyzed via linear combination fitting (LCF) over a range from −20 eV to +40 eV relative to the Co K-edge, using CoO, Co_3_O_4_, and fully reduced Co/CB-NP (for Co^0^) as reference materials. EXAFS fitting was performed on reduced catalyst samples by fitting *k* ^2^-weighted data in *R*-space (1 < *R* < 3 Å), limited to first-shell contributions. The number of floating parameters was chosen in accordance with the Nyquist criterion.^76^

Rietveld refinement of in situ XRD patterns was conducted using TOPAS 5.0 (Bruker). Instrumental parameters and the emission profile were determined using the NIST 660a LaB_6_ standard. To account for the support contribution in catalyst patterns, *ex situ* XRD data of the plain supports were fitted using a series of Split Pearson VII profiles, which were subsequently included in all refinements as a fixed background pattern. For Co/CB catalyst samples, only the scaling factor of the support background was refined. Crystalline phases were refined using structural models from database entries: Co hcp (COD-9008492), Co fcc (PDF-00-015-0806), CoO (COD-1541642), and Co_3_O_4_ (COD-9005896). Crystallite size-induced broadening was modeled with Lorentzian functions, while strain broadening was excluded. Additional refined parameters included a fifth-order Chebyshev background, sample displacement, zero error, and cylindrical 2θ correction. As reported in prior literature,^33^ due to the presence of a hcp–fcc intergrowth phase in the reduced catalysts, a reliable fit was only achieved by including preferred orientation refinements for the Co hcp (100) and (001) directions using the March–Dollase model.

### 2.9 Fischer–Tropsch Synthesis

Fischer–Tropsch synthesis runs were conducted in a stainless-steel tubular reactor with an internal diameter of 4 mm. For each experiment, 55–125 mg of Co/CB catalyst was diluted with 220–500 mg of SiC in a 1:4 g g^– 1^ ratio and fixed between two glass wool plugs. A type K thermocouple was inserted directly into the catalyst bed to monitor and control the reactor temperature. Catalyst reduction was performed *in situ* at atmospheric pressure by heating to 350 °C at a rate of 1 °C min^–1^ in a flow of 10 mL min^–1^ of 25 vol % H_2_ in Ar, maintained for 8 h. Following reduction, the reactor was cooled to 190 °C under 7.5 mL min^–1^ Ar and pressurized to 20 bar using the same Ar flow. Once 20 bar was reached, syngas was introduced at a total flow rate of 5 mL min^–1^, with a H_2_:CO ratio of 2.1 and 10 vol % Ar included as an internal standard. After complete replacement of Ar with syngas, the reactor was heated from 190 to 220 °C at 0.1 °C min^–1^ and held at 220 °C for 80 h. At the end of each reaction run, spent catalysts were passivated at room temperature and atmospheric pressure by flowing 10 mL min^–1^ of 1 vol % O_2_ in Ar. Product analysis was performed using online gas chromatography. A GC-MS system (Agilent GC7890B-MSD5977A) was employed, equipped with a flame ionization detector (FID) for quantifying C_2_–C_5_ hydrocarbons and a thermal conductivity detector (TCD) for analyzing CH_4_, CO, H_2_, and Ar. Carbon-based selectivities for C_1_–C_5_ products were calculated using the equation *S*_C_1_C_4__ = V̇_Cn_·*n*·(V̇_CO,in_ – V̇_CO,out_)^−1^, while the C_5+_ selectivity was calculated by *S*_C_5+__ = 1 – *S*_C_1_–C_4__.

## 3 Results and Discussion

### 3.1 Carbon Black Catalyst Supports with and without Surface Chemisorption Sites

Since it is not (yet) feasible to selectively introduce and characterize individual carbon surface defects on industrially relevant catalyst supports,^65,77^ this study focuses on their collective property as chemisorption sites for heteroatoms such as H, O, or Co. To compare the effects of these sites, two carbon catalyst supports were prepared that differ in the presence or absence of surface chemisorption sites, while retaining similar texture and morphology. Carbon black was selected as the base material due to its nonporous, nongraphitizing nature. These properties are advantageous because the high-temperature treatments required to anneal/remove surface chemisorption sites do not significantly alter the texture or morphology. Unlike materials with intrinsic porosity,^78^ the primary particles of carbon black do not collapse during thermal annealing, and its small crystallite size and disordered structure resist graphitization and thus major structural changes even at temperatures up to 2000 °C.^79^ In this context, carbon black was purified by acid leaching and divided into two parts, of which one was annealed at 1900 °C. Both the annealed (CB-1900) and nonannealed sample (CB) were subsequently treated at 950 °C in hydrogen, aiming to remove any electronegative surface species and to establish predominantly nonpolar surfaces. For CB-1900 no electronegative surface species were expected to remain after annealing at 1900 °C; the additional H_2_ treatment was therefore applied solely to ensure a comparable thermal history between the two materials and to account for any unforeseen effects of the H_2_ treatment. The resulting catalyst supports were expected to feature similar texture and morphology, but differ in terms of presence or absence of chemisorption sites, with the available chemisorption sites on CB covered by H.

N_2_ physisorption measurements of CB and CB-1900 indicated a similar texture, with closely matching isotherms and only slight deviations in low-pressure N_2_ uptake (*p*/*p*_0_ < 0.05, Figure 1a). These differences in uptake at low relative pressures—most likely due to variations in surface roughness or the closing of narrow interparticle voids during annealing—were also reflected in the BET surface areas, which nevertheless remained in a comparable range of 117 m^2^ g^–1^ for CB and 85 m^2^ g^–1^ for CB-1900. SEM and STEM imaging confirmed a similar morphology for both materials, revealing the characteristic hierarchical structure of carbon black with primary particles of 20–40 nm in diameter forming aggregates several hundred nanometers in size, which in turn assemble into loose agglomerates (Figures 1b,c and S1). STEM micrographs of CB display a turbostratic, onion-like arrangement of graphene layers within the primary particles, while for CB-1900 also 5–10 nm domains of higher crystallinity were observed, visible as dark spots in Figure 1c. This nanostructural transformation upon annealing at 1900 °C is further supported by XRD patterns (Figure S2). CB shows broad, diffuse reflections typical of largely amorphous carbon, which become significantly sharper after heat treatment, indicating increased local ordering. Similarly, Raman spectra of CB are characterized by broad D- and G-bands with negligible second-order features, whereas annealing at 1900 °C results in a notable narrowing of the D- and G-band fwhm’s and the emergence of a well-defined second-order D-band (Figure 1d). Despite these structural changes, annealing at 1900 °C does not lead to graphitization of the carbon black, indicated by an increase in the Raman *I*_D_/*I*_G_ ratio from 1.27 ± 0.05 for CB to 1.64 ± 0.07 for CB-1900 (Figure S3). In this context, the sp^3^/sp^2^ carbon ratio as determined from the XPS C 1s contribution changes only slightly from 0.27 for CB to 0.26 for CB-1900, indicating that high-temperature annealing does not strongly alter the carbon framework by graphitization but effectively removes volatile heteroatoms, replacing former C–X bonds with C–C bonds (Figures S4-S7). This conclusion is further supported by quantitative analysis of the Raman spectra and XRD patterns, showing that while the interlayer spacing (*d*-spacing) between graphene planes decreases from 0.365 nm in CB to 0.345 nm in CB-1900, the average crystallite size increases only mildly upon high temperature annealing (Figure 1e). In this context, the in-plane crystallite size (*L*_a_) grows from 5.6 nm for CB to 7.7 nm for CB-1900 while the stacking height of graphitic domains (*L*_c_) increases from 1.7 to 4.9 nm upon annealing at 1900 °C. Despite the modest increase in crystallite size, the oxidation stability, as probed by temperature-programmed oxidation (TPO, Figures 1f, S8), increased significantly after heat treatment, with the onset shifting by over 100 °C, from 598 °C for CB to 708 °C for CB-1900. Since it is well established that the carbon gasification rate is largely governed by the so-called “active surface area,” i.e., the quantity of surface sites susceptible to oxygen chemisorption,^80–82^ this result provides a first indication that, although texture, morphology, and crystallite size remain largely comparable, the heat treatment at 1900 °C effectively anneals reactive defect sites, rendering them unavailable for oxygen chemisorption.

**Figure 1:**
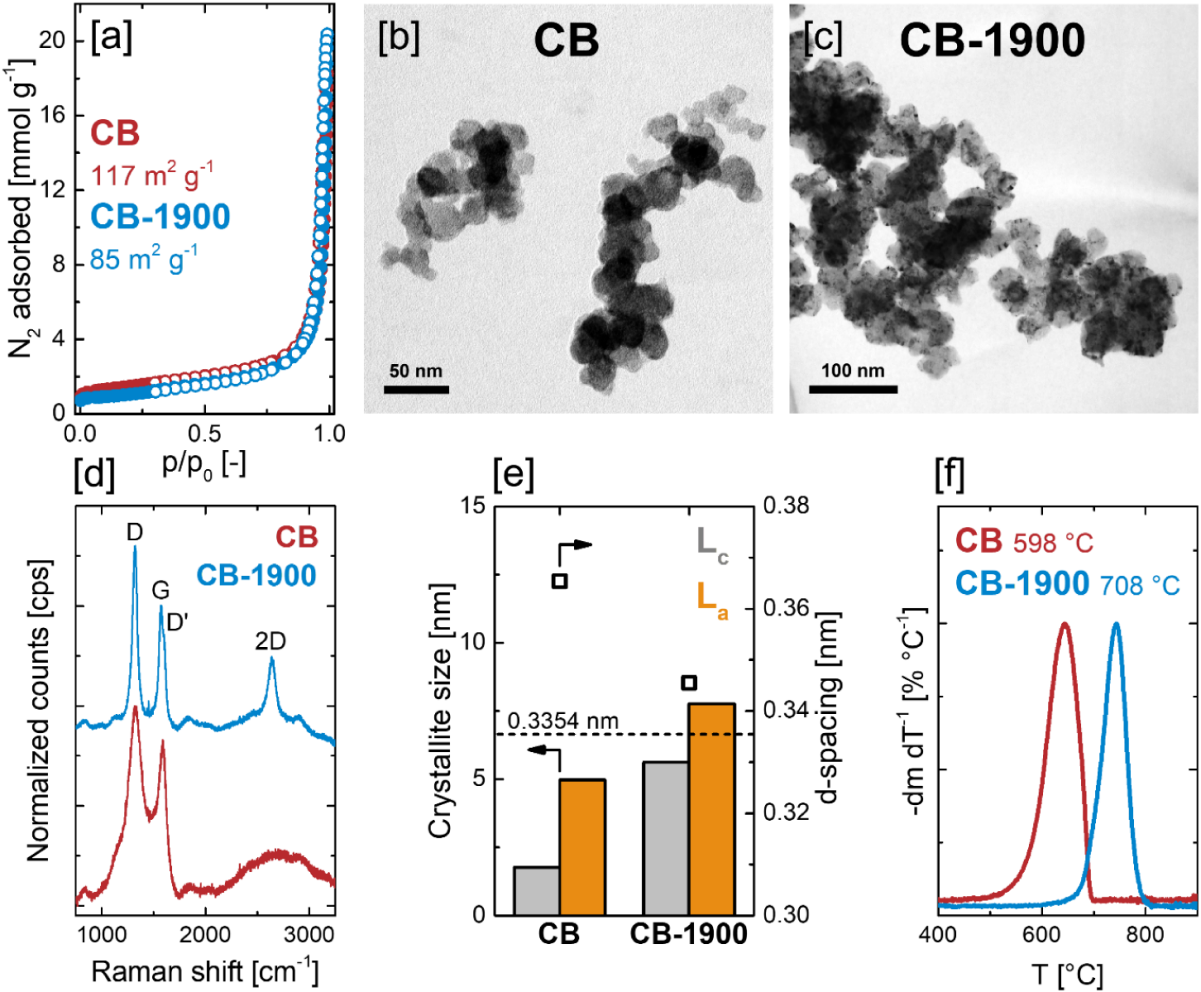
Characterization of the pristine supports. [a] N_2_ physisorption isotherms, [b, c] brightfield STEM micrographs and [d] Raman spectra of CB and CB-1900. [e] In-plane crystallite size (*L*_a_), stacking height (*L*_c_) and interlayer distance (*d*-spacing) for CB and CB-1900, obtained by quantitative analysis of Raman spectra and XRD patterns. [f] Temperature-programmed oxidation of CB and CB-1900 in synthetic air with corresponding onset temperatures.

To characterize the quantity of chemisorption sites on the pristine catalyst supports, temperature-programmed desorption (TPD) experiments were conducted up to 1400 °C, monitoring the release of CO and CO_2_ as indicators of chemisorbed O species, and H_2_ as a marker for chemisorbed H (Figure 2a,b; see also Figure S9a for corresponding mass loss curves). The TPD results reveal a strong difference between CB and CB-1900, with CB exhibiting a high total emission of surface species dominated by H_2_, indicating a surface rich in chemisorption sites (1986 μmol g^–1^) of which 95% are saturated by H. In contrast, CB-1900 shows only minimal emissions of CO_*x*_ and H_2_, reflecting a surface largely devoid of chemisorption sites (74 μmol g^–1^). In this context, it is important to note that the maximum desorption temperature of the TPD setup (1400 °C) does not enable full desorption of hydrogen species from CB. As such, the actual difference in chemisorption site density between CB and CB-1900 is probably even more pronounced than indicated by these measurements.^83^

**Figure 2:**
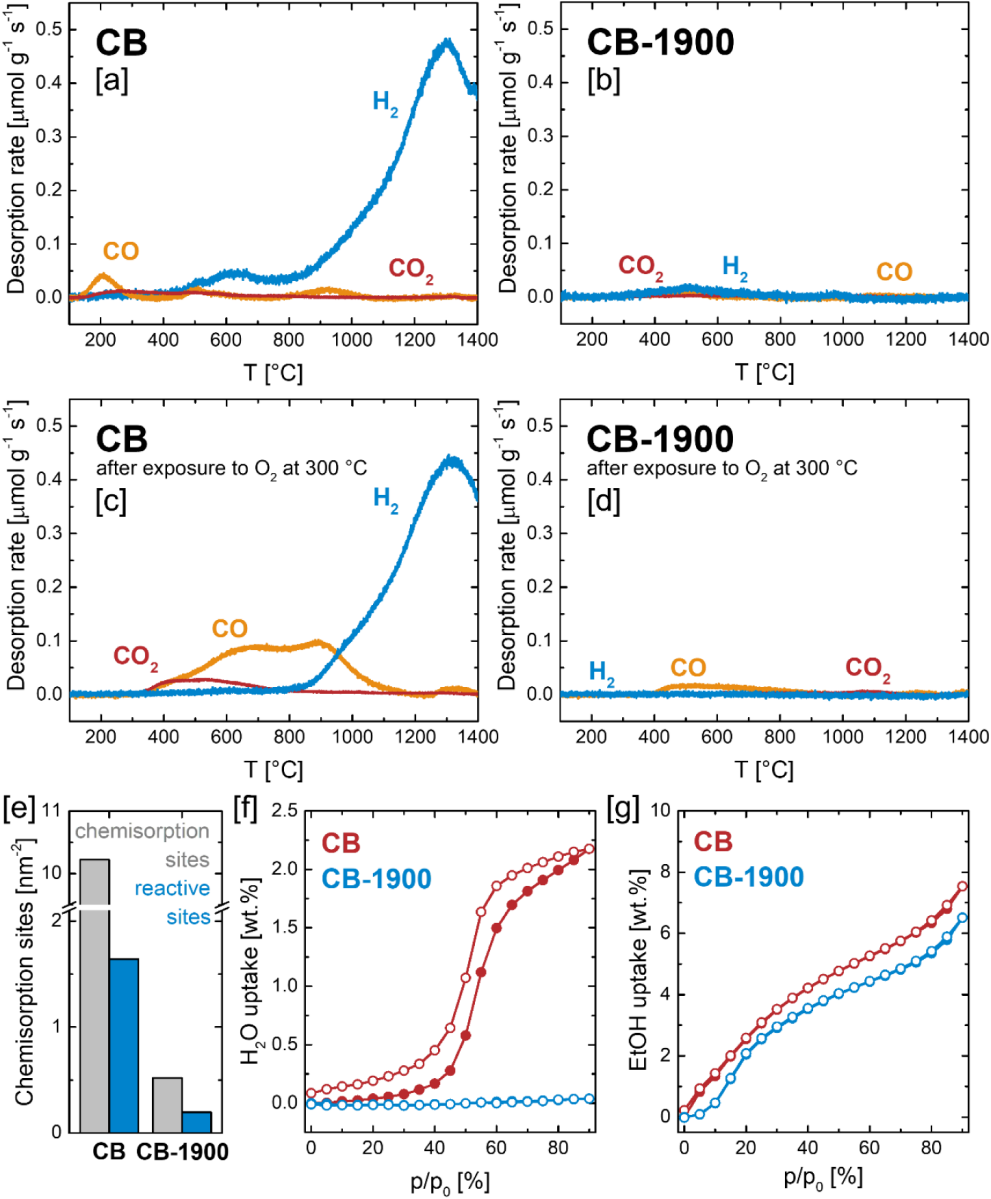
Temperature-programmed desorption of pristine [a] CB and [b] CB-1900 (35 mg carbon black, 50 mL min^–1^ Ar, 10 °C min^–1^ to 1400 °C). Temperature-programmed desorption of [c] CB and [d] CB-1900 after preadsorption of O_2_ in synthetic air at 300 °C for 1 h. [e] Total surface density of chemisorption sites and density of chemisorption sites susceptible to oxygen chemisorption of CB and CB-1900. Vapor adsorption isotherms at 25 °C of CB and CB-1900 using [f] H_2_O and [g] EtOH as molecular probes.

To further assess the reactivity of the surface chemisorption sites, an O_2_ preadsorption step was performed at 300 °C prior to TPD (Figure 2c,d; see Figure S9b for the corresponding mass loss curves). This temperature was selected to ensure fast equilibration of O_2_ chemisorption while remaining low enough to prevent carbon gasification, as guided by the TPO results (Figure 1f). For CB, the O_2_ pretreatment led to a substantial increase in CO and CO_2_ emissions during TPD, confirming significant oxygen chemisorption. This increase was accompanied by a corresponding decrease in H_2_ emission, while the total quantity of chemisorption sites remained unchanged. Quantification suggested that approximately 16% (319 μmol g^–1^) of the initial H-occupied sites on CB are reactive toward O chemisorption. A similar, though much weaker, response was observed for CB-1900. Following O_2_ preadsorption, CO and CO_2_ emissions during TPD increased slightly, indicating limited but detectable oxygen chemisorption. Again, the total number of chemisorption sites remained nearly constant, implying that H-occupied sites on CB-1900 are also reactive toward O chemisorption. In this case, the number of reactive sites was determined to be 38 μmol g^–1^. When normalized to the specific surface area, CB exhibits a total chemisorption site density of 10.2 nm^–2^, compared to 0.5 nm^–2^ for CB-1900 - a difference by a factor of approximately 20, implying that high-temperature annealing at 1900 °C removes around 95% of the chemisorption-active surface sites (Figure 2e). Considering the “reactive” fraction of these sites, i.e., those available for oxygen chemisorption, the difference is slightly smaller, with site densities for CB and CB-1900 determined to be 1.6 nm^–2^ and 0.2 nm^–2^, respectively, resulting in CB featuring an 8-fold higher surface density of reactive sites than CB-1900.

To further investigate the surface characteristics of both catalyst supports, H_2_O and ethanol (EtOH) vapor sorption isotherms were recorded at 25 °C (Figure 2f,g). In this context, H_2_O adsorption on carbon materials is governed predominantly by polar interactions, such as hydrogen bonding, which require the presence of primary adsorption sites carrying localized (partial) charges. These primary sites serve as initial anchors for water molecules, which in turn can form hydrogen bonds with additional water molecules, facilitating the nucleation and growth of water surface clusters at higher partial pressures.^84,85^ The H_2_O sorption isotherms reveal pronounced differences between CB and CB-1900. CB exhibits very low but measurable H_2_O uptake at low relative pressures (p/p_0_ < 0.4), indicating the presence of a limited number of primary adsorption sites capable of initiating water adsorption. This initial uptake suggests that, despite its highly hydrophobic nature, CB retains a sufficient population of surface sites with partial charges to support localized polar interactions with water and in consequence significant H_2_O uptake at higher partial pressures. Despite similar textural properties (Figure 1a), CB-1900 shows negligible H_2_O adsorption across the entire pressure range, indicating an absence of such primary adsorption sites. This lack of initial interaction prevents subsequent water cluster formation and pore filling, highlighting the strongly deactivated and nonpolar character of the surface of CB-1900 following high-temperature annealing. In contrast to H_2_O, vapor sorption isotherms using EtOH as a molecular probe revealed similar adsorption behavior for both CB and CB-1900 (Figure 2g). The isotherms display the characteristic regions of mono- and multilayer adsorption as the EtOH partial pressure increases, consistent with physisorption driven by nonspecific dispersion interactions.^86,87^ The total EtOH uptake at *p*/*p*_0_ = 0.9 falls into a comparable range with 7.5 wt % for CB and 6.5 wt % for CB-1900, especially if the difference in SSA as determined by N_2_ physisorption is taken into account (117 m^2^ g^–1^ for CB and 85 m^2^ g^–1^ for CB-1900). From a practical standpoint, these findings suggest a similar overall affinity of EtOH for both materials, indicating that ethanol is a suitable solvent for catalyst synthesis via incipient wetness impregnation for both CB and CB-1900. This comparable sorption behavior ensures consistent wetting and drying dynamics during Co­(NO_3_)_2_ infiltration for both supports, effectively minimizing the influence of carbon/solvent interactions as a variable affecting catalyst dispersion during IWI and allowing to directly investigate the role of carbon surface defects on catalyst preparation, activation and performance.

### 3.2 Pristine Co/CB Catalysts

To achieve cobalt loadings of approximately 6 wt %, cobalt nanoparticles were deposited onto the carbon black supports using two different methods (Table 1). To this end, catalysts were prepared by incipient wetness impregnation of Co­(NO_3_)_2_ in ethanol followed by calcination (250 °C, Ar), and wet impregnation of colloidal CoO nanoparticles with a narrow size distribution and an average diameter of 7.4 ± 0.7 nm as determined by STEM imaging (Figure 3a,b). Colloidal CoO NP’s were synthesized via thermal decomposition of Co_2_(CO)_8_ in the presence of oleic acid, followed by air exposure at room temperature. In the following, catalysts prepared via colloidal nanoparticle deposition are denoted with the suffix -NP, while those prepared by incipient wetness impregnation are labeled -IWI. Unless otherwise noted, all reported Co particle sizes refer to metallic cobalt, corrected for a 3 nm cobalt oxide shell using the procedure described by Eschemann et al.^38^

**Figure 3:**
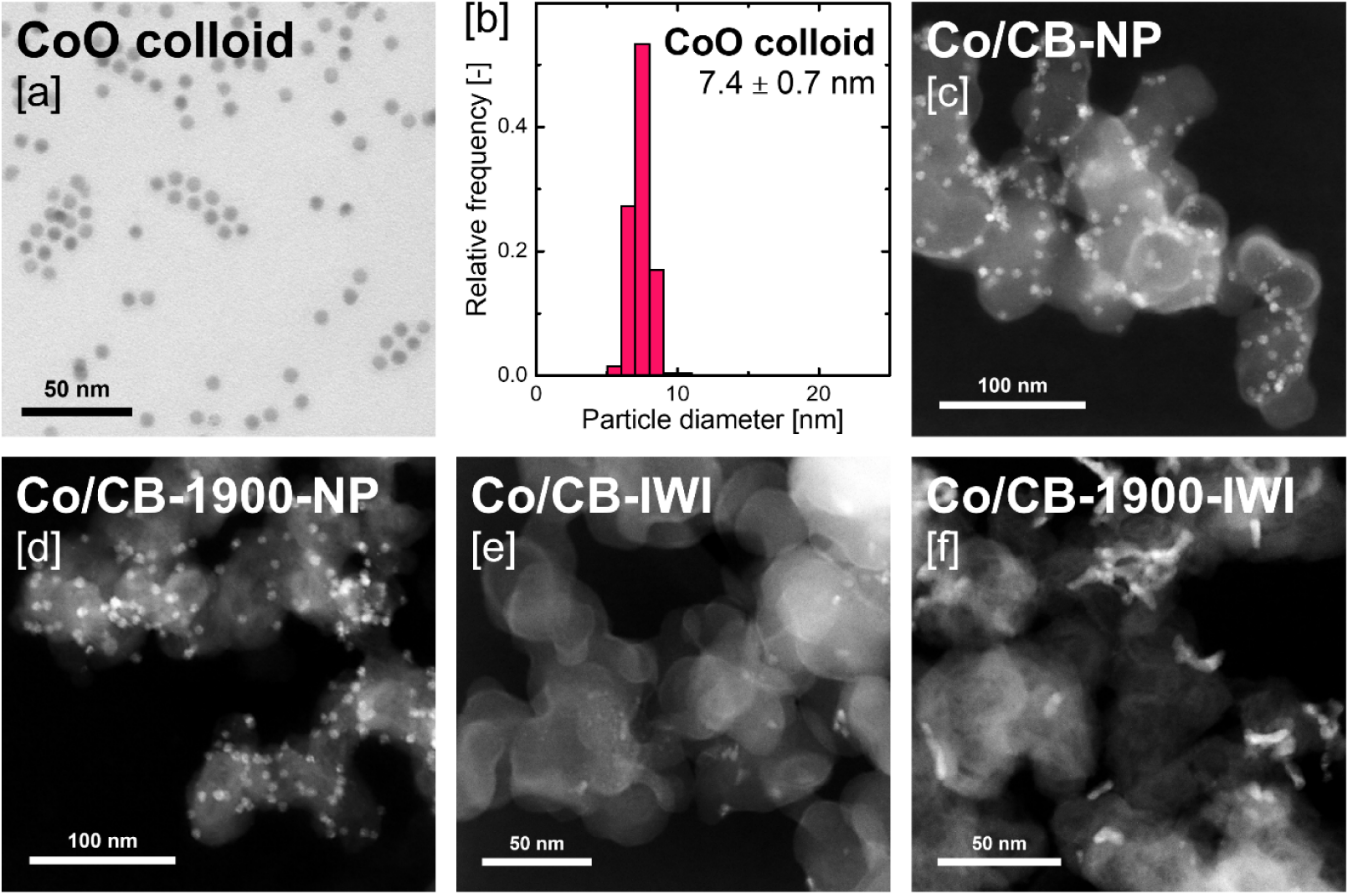
[a] Brightfield-STEM image and [b] the corresponding Co particle size distribution of the colloidal cobalt nanoparticles. HAADF-STEM micrographs of pristine [c] Co/CB-NP, [d] Co/CB-1900-NP, [e] Co/CB-IWI, and [f] Co/CB-1900-IWI.

**Table 1:** Properties of Pristine Catalysts

Sample	Co loading^a^ [wt.%]	*D* (CoO_*x*_)^b^ [nm]	*D* (Co^0^)^b^ [nm]	Co_3_O_4_ ^c^ [at.%]	CoO^c^ [at.%]	Co^0^ ^c^ [at.%]	CoO crystallite size^d^ [nm]	Co_3_O_4_ crystallite size^d^ [nm]
Co NP colloid	-	7.4 ± 0.7	5.6 ± 0.7	15	83	2	-	-
Co/CB-NP	7.0	7.2 ± 0.7	5.7 ± 0.7	23	74	3	3.5	-
Co/CB-1900-NP	6.9	7.0 ± 0.6	5.5 ± 0.6	15	81	4	3.7	-
Co/CB-IWI	5.6	4.1 ± 2.0	3.2 ± 1.7	95	5	-	2.3	22.4
Co/CB-1900-IWI	4.6	6.6 ± 1.9	5.2 ± 1.8	81	18	-	2.1	3.9

a As determined by MP-AES.

b Average Co particle diameter obtained by STEM imaging. The Co^0^ diameter is determined by correcting for a 3 nm oxide layer.

c Determined by XANES LCF analysis.

d Determined by Rietveld analysis of XRD patterns.

STEM analysis of the as-prepared catalysts revealed a homogeneous cobalt distribution across both Co/CB-NP and Co/CB-1900-NP, with no observable aggregation (Figure 3c,d). The mean Co^0^ particle sizes of 5.7 ± 0.7 nm and 5.5 ± 0.6 nm, respectively, closely matched the size of the original colloidal nanoparticles of 5.7 ± 0.7 nm (Table 1). Similarly, the IWI-prepared samples showed a largely homogeneous Co distribution, however, average cobalt particle sizes varied significantly between the two supports (Figures 3e,f and S10). Co/CB-IWI exhibited a markedly smaller average Co^0^ particle diameter (3.2 ± 1.7 nm), while Co/CB-1900-IWI showed a larger mean particle size (5.2 ± 1.8 nm) comparable to the samples prepared with colloidal Co nanoparticles, albeit with a wider size distribution. Given identical impregnation procedures and similar solvent/support affinities, the differences in Co particle size of the IWI samples are most likely a consequence of the different chemisorption site densities of the supports. Despite their nonpolar character (being overwhelmingly saturated by H), chemisorption sites may serve as adsorption and nucleation centers for Co during impregnation/calcination, with the higher site density of CB giving rise to smaller Co nanoparticles as compared to CB-1900. XANES-LCF analysis of the pristine catalysts revealed clear differences in cobalt oxidation states depending on the preparation method (Table 1, Figure S11). The NP-based samples were dominated by CoO (74–83%), with minor contributions from Co_3_O_4_ (15–23%) and a negligible fraction of metallic Co^0^ (2–4%). In contrast, IWI followed by calcination in inert atmosphere yielded Co_3_O_4_-rich (81–95%) Co nanoparticles, balanced by smaller amounts of CoO with no detectable traces of metallic Co. XPS analysis of the Co 2p_3/2_ region showed consistent binding energies between 780.3 and 780.5 eV for all catalyst materials (Figure S12, for Raman analysis of the pristine catalysts see Figure S13 and sidenote S2). Considering the binding energies for CoO (780.7 eV) and Co_3_O_4_ (780.1 eV),^88^ these results indicate that the Co nanoparticle surface in all samples consisted of a mix of CoO and Co_3_O_4_. Rietveld refinement of XRD patterns yielded phase compositions largely comparable to those determined by XANES-LCF for all samples (Figures S14, S15). Average CoO crystallite sizes were determined to be 3.5 and 3.7 nm for Co/CB-NP and Co/CB-1900-NP, respectively, which is significantly smaller than the mean particle sizes in the oxidic state as observed by STEM (∼7 nm, Table 1). This finding indicates the presence of polycrystalline Co nanoparticles, which is consistent with literature reports.^33^ A similar trend was observed for Co/CB-1900-IWI, where CoO (2.1 nm) and Co_3_O_4_ (3.9 nm) crystallite sizes were also notably smaller than the STEM-determined particle diameter (6.6 nm in the oxidic state), again suggesting polycrystallinity. A clear deviation from this trend was observed in Co/CB-IWI. While the CoO crystallite size (2.3 nm) remained smaller than the average particle diameter (4.1 nm in the oxidic state), the Co_3_O_4_ crystallite size determined by Rietveld refinement was surprisingly large at 22.4 nm. This discrepancy points to the presence of at least some larger Co_3_O_4_ particles, which were also observable via STEM imaging, albeit at a very low abundance (see STEM overview images in Figure S10).

### 3.3 Co Nanoparticle Growth during Catalyst Reduction

Upon catalyst reduction (1 °C min^–1^ to 350 °C, 8 h hold at 350 °C, 25 vol.% H_2_ in Ar), significant cobalt nanoparticle growth was observed, with the extent of sintering dependent on both the support material and the synthesis method (Figure 4, see Figure S16 and Side note S1 for information on catalyst reducibility). Utilization of STEM imaging after reduction and passivation showed Co particles on Co/CB-NP increased in size by 67%, growing from 5.7 to 9.5 nm (Figures S17–S18). In contrast, Co/CB-1900-NP exhibited substantially greater sintering, with particle diameters increasing by 111% (from 5.5 to 11.6 nm). A similar trend was observed for the IWI-derived catalysts. Co particles on Co/CB-IWI grew modestly by 13%, from 3.2 to 3.6 nm, while Co/CB-1900-IWI showed a more pronounced increase of 54%, with particle sizes rising from 5.2 to 8.0 nm. These results imply that the defect-rich surface of CB exerts a stabilizing effect, more effectively inhibiting cobalt nanoparticle growth than the defect-poor surface of CB-1900. It is also evident that, beyond the influence of the support, the synthesis method plays a role. The lower degree of particle growth observed in the IWI samples, compared to their colloidal counterparts, points to a stronger Co-support interaction in these systems. This enhanced interaction is likely rooted in the calcination step, with processes such as nitrate decomposition, cobalt nucleation and nanoparticle growth possibly promoting a preliminary stabilization of cobalt species which is absent in the colloidal approach.

**Figure 4:**
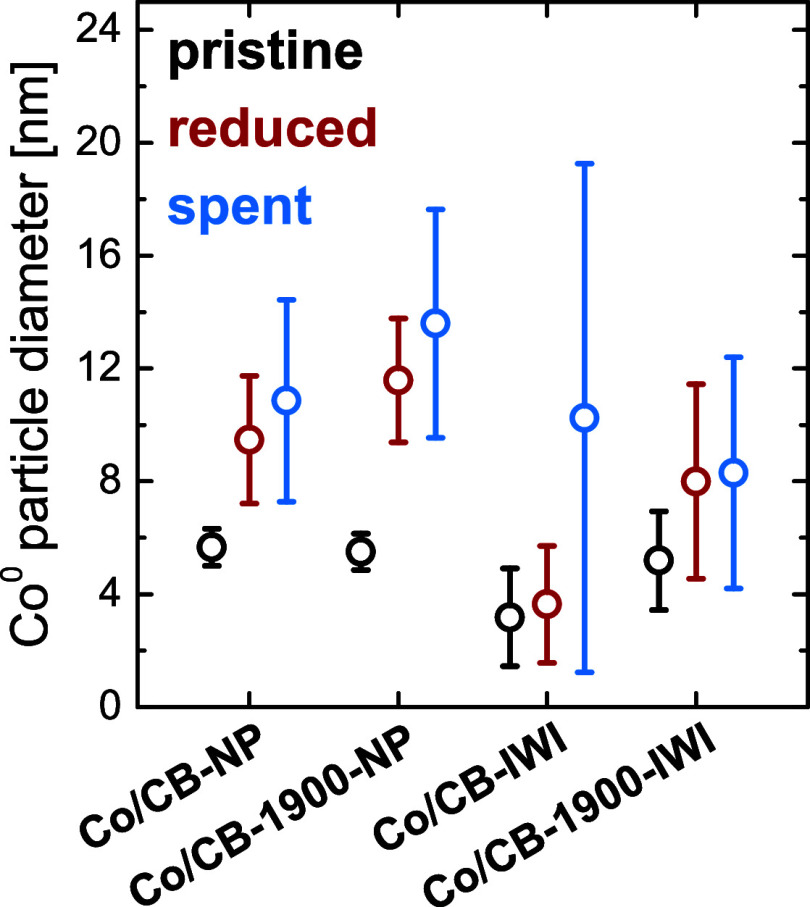
Average Co^0^ particle diameters of the Co/C catalysts in the pristine state, after reduction/passivation and after 80 h FTS/passivation. See Figure S18 for Co particle size distributions of the pristine, reduced and spent catalysts.

While it seems evident that the defect density of the carbon support surface significantly influences the extent of cobalt nanoparticle sintering during catalyst reduction, the specific growth pathways remain to be elucidated. To address this, a combination of high-resolution SEM and STEM imaging was employed, focusing on the reduced and passivated samples prepared using colloidal Co nanoparticles. A key advantage of this approach lies in the use of high-resolution secondary electron imaging via HR-SEM, which provides surface sensitivity and a large depth of field. This is particularly important for distinguishing true nanoparticle interactions from apparent overlaps that may result from the 2D projection inherent to STEM imaging. Equally important is the focus on samples prepared with colloidal Co NP’s, as their regular spherical morphology allows identification of coalescence events with greater confidence, while their narrow size distribution, specifically, the absence of particles <4 nm in both the original colloid and the pristine catalysts, serves as a reliable baseline to detect Co NP shrinking. Using this approach, nanoparticle coalescence emerged as the dominant sintering pathway for both Co/CB-NP and Co/CB-1900-NP, as numerous coalescence events could be identified for both defect-rich CB and defect-poor CB-1900 (Figures 5, S19). In contrast, Ostwald ripening—as indicated by a subset of shrinking Co nanoparticles^89^—seemed negligible, as Co nanoparticles smaller than the threshold of the original Co colloid could not be found in any meaningful quantity on either support (see Co particle size distributions, Figure S18). Considering the influence of the catalyst support on cobalt nanoparticle growth, a quantitative estimate of the average number of pristine Co nanoparticles required to coalesce in order to reach the observed postreduction particle size yields a clear difference between the two systems. For Co/CB-NP, assuming spherical geometry and growth from 5.7 to 9.5 nm, approximately 4.6 pristine nanoparticles must merge. In contrast, for Co/CB-1900-NP, more than twice as many, e.g., 9.4 nanoparticles, must coalesce to achieve the final average diameter of 11.6 nm starting from pristine Co NP’s of 5.5 nm. STEM imaging indicated that Co nanoparticles were well dispersed on both pristine Co/CB-NP and Co/CB-1900-NP with no evidence of larger clusters/agglomerates (Figure 3), implying that coalescence during reduction requires substantial lateral migration of nanoparticles across the support surface. The fact that, on average, more than twice the number of Co nanoparticles coalesce on CB-1900 than on CB during reduction thus indicates that surface defects on CB likely act as anchoring points, restricting nanoparticle mobility and thereby limiting the extent of sintering.

**Figure 5:**
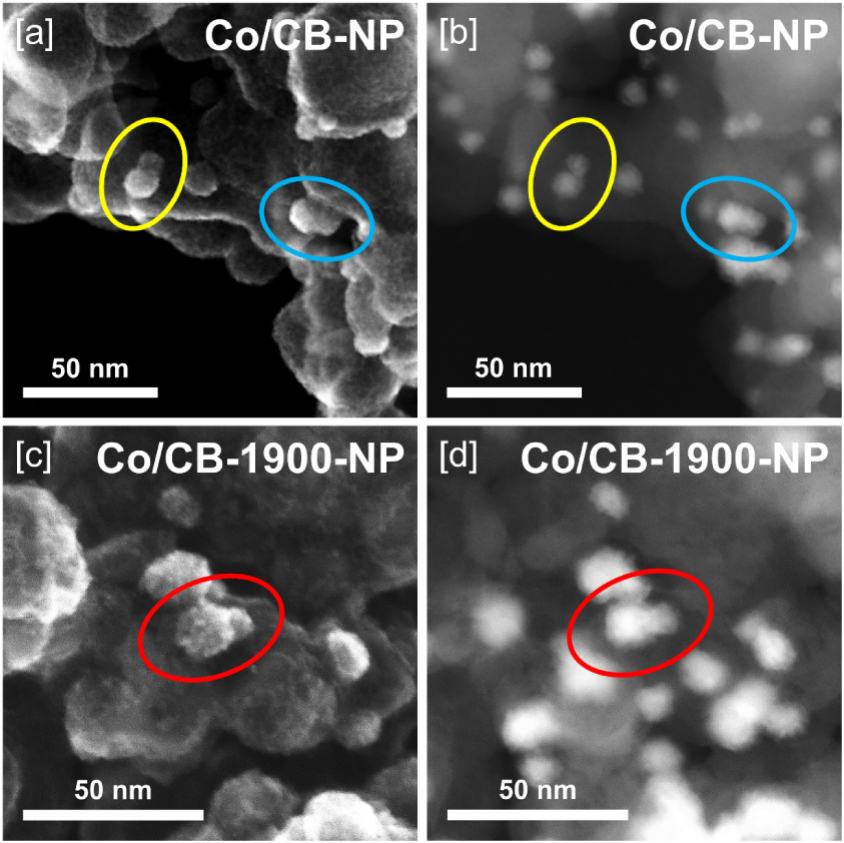
Combined high-resolution SEM/HAADF-STEM imaging at identical locations for reduced and passivated [a, b] Co/CB-NP and [c, d] Co/CB-1900-NP.

### 3.4 Co Nanoparticle Anchoring during Catalyst Reduction

Despite predominantly nonpolar surfaces, cobalt nanoparticles display distinct differences in anchoring behavior on CB and CB-1900, raising the fundamental question of how nanoparticle anchoring occurs on carbon supports in the absence of polar interactions that are conventionally associated with metal–support anchoring. In this context, catalyst reduction (3 °C min^–1^ to 350 °C, 2–6 h hold at 350 °C, 25 vol.% H_2_ in He) was followed by combined in situ XANES/XRD, aiming to simultaneously track changes in Co phase as well as Co crystallite size (Figures 6, S20–21). In addition, XPS and Raman spectroscopy was carried out before and after catalyst reduction, to evaluate changes in the carbon structure upon catalyst reduction (Figures S4-S7, S13 and S22, see Side note S2 in the Supporting Information for a detailed discussion of the Raman results). While crystallite size determination for the cobalt oxides (e.g., Co_3_O_4_ and CoO) was conducted via standard Rietveld refinement, catalyst reduction yielded a well-known Co fcc/hcp intergrowth face, which complicated direct crystallite size analysis via Rietveld refinement.,^31,33,90^ To circumvent this limitation, the full width at half-maximum (fwhm) of the overlapping Co^0^ (110)­hcp/(022)­fcc reflection at ∼11.2°/2θ was used as a relative measure of crystallite size (Figure S21). This reflex is known to be largely unaffected by the stacking disorder induced by fcc/hcp intergrowth^90^ and was thus previously identified as a viable relative descriptor for Co crystallite size with the fwhm being inversely proportional to Co crystallite size.^31,43^ In parallel, the off-gas composition during reduction was monitored by online mass spectrometry (Figure 6). H_2_O evolution (*m*/*z* 18) was tracked to follow the reduction process, while *m*/z 15 (CH_3_^+^) was used as a generic marker for hydrocarbon emission, intended for example to monitor ligand (e.g., oleic acid) decomposition in the samples prepared with colloidal Co NP. Additionally, CO_2_ evolution (*m*/z 44) was recorded.

**Figure 6:**
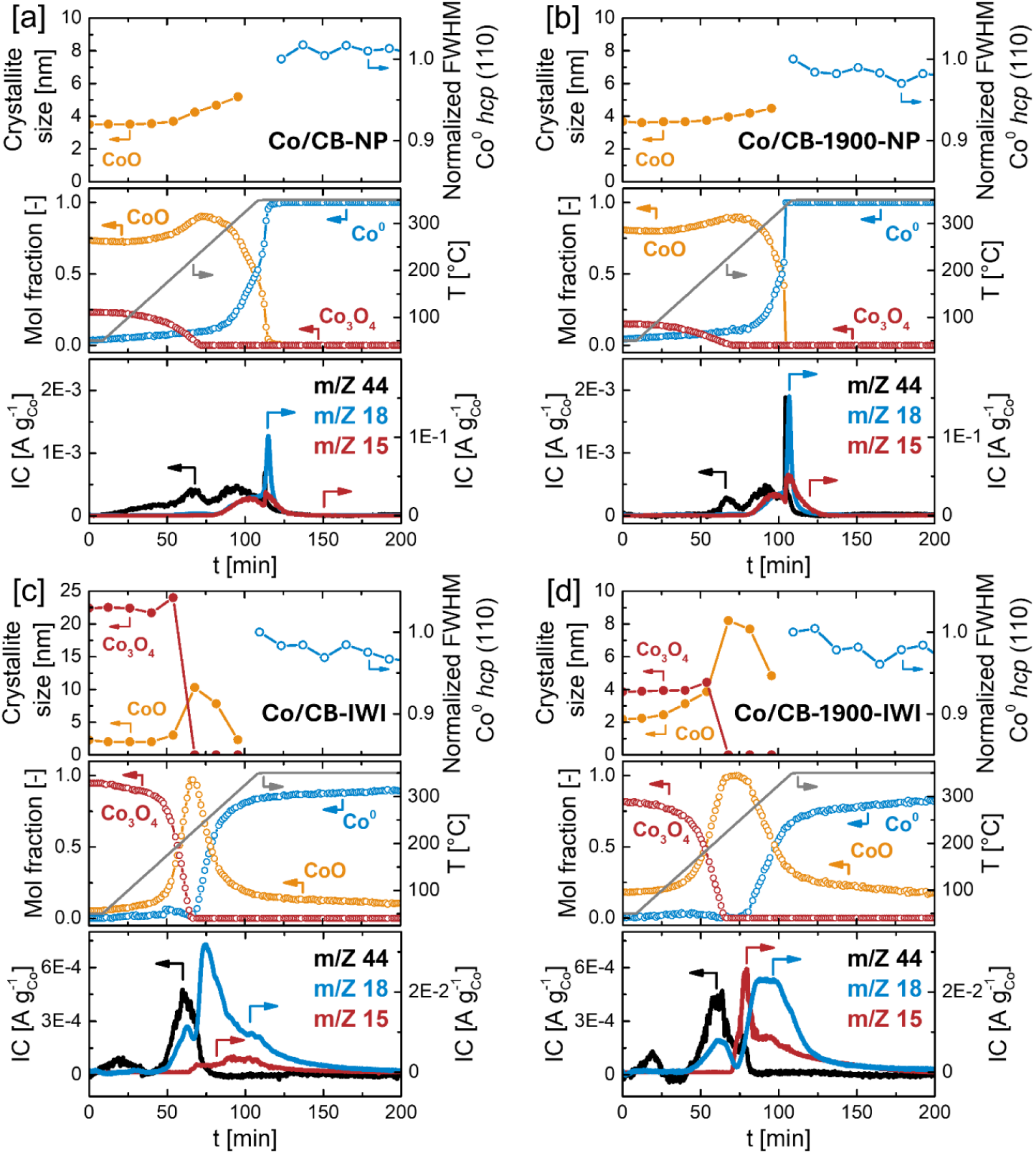
Combined analysis of Co crystallite sizes by in situ XRD, Co phase composition by in situ XANES and off-gas composition by online mass spectrometry during catalyst reduction with 25 vol.% H_2_ in He and a temperature ramp of 3 °C min^–1^ to 350 °C with subsequent hold at 350 °C for [a] Co/CB-NP, [b] Co/CB-1900-NP, [c] Co/CB-IWI and [d] Co/CB-1900-IWI.

In situ XANES-LCF analysis of the reduction experiments revealed the typical two-step reduction for all samples (Figure 6a–d). The first step, corresponding to the reduction of Co_3_O_4_ to CoO, was completed before 250 °C (within 75 min) for all materials. This was immediately followed by the subsequent reduction of CoO to metallic Co^0^. For the colloidal NP-based catalysts, this second reduction step proceeded rapidly and was completed with a degree of reduction (DOR) of 100% shortly after reaching 350 °C. In contrast, for the IWI-derived samples, the CoO to Co^0^ transformation continued over the full 6-h hold at 350 °C, ultimately leveling off at DOR’s of 92% for Co/CB-IWI and 90% for Co/CB-1900-IWI, with the remaining cobalt being present as CoO (Figure 6, S16b).

For both Co/CB-NP and Co/CB-1900-NP samples, an increase in CoO crystallite size was observed beginning around 175 °C (50 min), coinciding with the first reduction step from Co_3_O_4_ to CoO (Figure 6a,b). This initial growth was likely not due to sintering but rather reflected the phase transition within individual nanoparticles during which Co_3_O_4_ crystallites are shrinking and are replaced by growing CoO domains. However, even after the completion of this first reduction step and the onset of CoO reduction to metallic Co^0^ (starting at ∼250 °C, 75 min), the CoO crystallite size continued to increase in both samples. This continued growth is a clear indicator of sintering, as CoO domains would typically be expected to shrink during reduction to Co^0^. Following complete reduction (at 350 °C, *t* > 110 min), the fwhm of the Co^0^ hcp (110) reflection in Co/CB-NP (normalized to its value in the first diffractogram after complete reduction) remained stable, suggesting that Co nanoparticles have been effectively anchored to the CB surface. In contrast, Co/CB-1900-NP showed a slight decrease in the fwhm of the Co^0^ hcp (110) peak beyond the point of full reduction (*t* > 100 min), indicating continued growth of Co crystallites. As no additional phase transformation occurred beyond this point, this growth must be attributed to ongoing sintering, implying less efficient nanoparticle anchoring on CB-1900 as compared to CB. Taken together, these results suggest that Co nanoparticle sintering in both systems occurred predominantly within a relatively narrow window from approximately 50–110 min (175 to 350 °C), which coincided with intensive phase transformations (Co_3_O_4_ → CoO → Co^0^). After complete reduction, further particle growth was not observed in Co/CB-NP, indicating that effective anchoring has occurred during this time/temperature window. In contrast, the continued growth observed in Co/CB-1900-NP suggests that anchoring on this support was incomplete or less effective, allowing sintering to proceed after full reduction.

Off-gas analysis by mass spectrometry during the reduction of Co/CB-NP and Co/CB-1900-NP revealed H_2_O emission profiles (*m*/*z* 18) that closely mirror the corresponding H_2_-TPR profiles (Figure S16a, Side note S1), with the dominant water peak occurring between 265 – 350 °C (80–125 min), reflecting the rapid reduction of CoO to Co^0^ (Figure 6a,b). The *m*/*z* 15 signal of Co/CB-NP and Co/CB-1900-NP, indicative of hydrocarbon emission (e.g., CH_3_^+^), followed a similar qualitative trend, indicating the decomposition/desorption of oleic acid ligands upon formation of metallic Co^0^. This correlation between ligand decomposition and the emergence of Co^0^ aligns well with previous studies employing an identical colloidal Co nanoparticle system for FTS.^31,72^ The decomposition of oleic acid was further reflected in the CO_2_ emission profile (*m*/*z* 44), which showed a broad emission peak between 80 and 110 min, followed by a sharp CO_2_ spike at ∼115 min, coinciding with the highest rate of reduction of CoO to Co^0^.

Beyond the expected signals associated with cobalt reduction and ligand decomposition, a pronounced, low-temperature CO_2_ emission feature was observed around 67 min (∼225 °C) for both Co/CB-NP and Co/CB-1900-NP. While Co/CB-NP exhibited a broad, distinct emission peak, the corresponding signal in Co/CB-1900-NP was significantly weaker, corresponding to ∼34% of the CO_2_ emission of Co/CB-NP between 0 and 75 min. This early CO_2_ emission cannot be attributed to ligand decomposition as no concurrent hydrocarbon signal at *m*/*z* 15 is detected, nor does it match the desorption or decomposition of native surface oxides on the carbon supports (see TPD analysis of the pristine supports (Figure 2a,b)). Interestingly, this low-temperature CO_2_ release coincided with the first reduction step of Co_3_O_4_ to CoO, suggesting carbothermal reduction of Co_3_O_4_ as a possible source of CO_2_. In this context, oxygen would be transferred from the cobalt oxide phase to the carbon support, resulting in localized carbon gasification and the release of CO_*x*_ species. This oxygen transfer requires a chemically reactive carbon surface capable of oxygen chemisorption and depends as such on the availability of reactive chemisorption sites on the carbon surface. In this sense, the observed difference in CO_2_ emission between Co/CB-NP and Co/CB-1900-NP aligned well with the respective differences in the density chemisorption site reactive to O chemisorption of CB (1.6 nm^–2^) and CB-1900 (0.2 nm^–2^, Figure 2e), meaning that CB participated more actively in carbothermal reduction due to its greater capacity to accept oxygen at the Co/C interface.

In order to trace changes in the carbon support surface consistent with carbon gasification, the sp^3^/sp^2^ carbon ratio was extracted from the XPS C 1s contribution of the pristine supports, the Co-loaded materials as well as the reduced/passivated catalysts (Figure S4–S7). The loading of the nanoparticles lead to a clear increase in the sp^3^/sp^2^ ratios as compared to the pristine supports for both Co/CB-NP (0.27 to 0.34) and Co/CB-1900-NP (0.26 to 0.40). As direct chemical interactions between the carbon surface and the nanoparticles during wet impregnation are considered to be unlikely, this increase is attributed to the presence of oleic acid ligands rather than to changes in the carbon surface structure. Hence, upon catalyst reduction, the sp^3^/sp^2^ ratio decreased significantly for both Co/CB-NP (from 0.34 to 0.31) and Co/CB-1900-NP (from 0.40 to 0.25), in line with the decomposition of oleic acid ligands during reductive activation of Co/C catalysts.^31,72^ Notably, after reduction and ligand decomposition, the C 1s spectrum of Co/CB-1900-NP (as well as the sp^3^/sp^2^ ratio) becomes nearly identical to that of the pristine CB-1900 support, indicating that catalyst reduction does not induce detectable structural modifications in the defect-poor CB-1900 support. By contrast, even after ligand decomposition during reduction, the sp^3^/sp^2^ ratio of Co/CB-NP (0.31) remains substantially higher than that of pristine CB (0.27). This residual increase suggests that the carbon surface structure of CB changes during Co reduction, as would be expected if carbon gasification at the Co/C interface generated new defect sites.

Considering the IWI samples, in situ observation of cobalt sintering was found to be more challenging for both Co/CB-IWI and Co/CB-1900-IWI, despite significant cobalt particle growth during catalyst reduction (Co/CB-1900-IWI: 5.2 nm to 8.0 nm, Figures 4, 6c,d, S18). In both samples, changes in cobalt crystallite size corresponded to the simultaneously observed phase transformations, making it difficult to isolate sintering phenomena from the effects of Co phase change. In this sense, during the first reduction step (Co_3_O_4_ → CoO, 40–75 min, 140–250 °C), Co_3_O_4_ domains shrank while CoO domains grew for both Co/CB-IWI as well as Co/CB-1900-IWI. Similarly, in the second reduction step (CoO → Co^0^, from ∼75 min, >250 °C), CoO domains shrank as expected, while the subsequent isothermal hold at 350 °C afforded a gradually increasing Co^0^ content which was accompanied by a decrease in the fwhm of the Co^0^ hcp (110) reflection, indicative of crystallite growth. While sintering analysis via in situ XRD was challenging for Co/CB-IWI—due to both minimal Co particle growth during reduction (from 3.2 to 3.6 nm, see Figure 4, S18) and the presence of a minor fraction of larger (>20 nm) Co particles—there is evidence that sintering in Co/CB-1900-IWI takes place within a similar time frame and temperature window as observed for the colloidal Co nanoparticle systems. In this context, CoO crystallite size in Co/CB-1900-IWI reached a maximum of 8.2 nm at 68 min (230 °C), which was already significantly larger than the pristine average Co particle diameter (6.6 nm in the oxidic state, with Co_3_O_4_ as the dominant phase, see Table 1). This increase in CoO crystallite size beyond the pristine average particle diameter strongly indicates sintering, as generally Co particle shrinking would be expected at this stage due to the reduction of Co_3_O_4_ to CoO.

Considering off-gas analysis, the water emission profiles for both Co/CB-IWI and Co/CB-1900-IWI reflected the Co reduction steps observed by in situ XANES and H_2_-TPR well, featuring a smaller H_2_O release for the Co_3_O_4_-to-CoO transition and a dominant emission peak for the CoO-to-Co^0^ reduction (see Figure 6c,d, S16a,b and Side note S1). Similar to the samples prepared with colloidal Co NP, low-temperature CO_2_ emission maxima in the 200–220 °C range—attributed to carbothermal reduction of Co_3_O_4_—were prominently featured for both Co/CB-IWI and Co/CB-1900-IWI. Consistent with the absence of ligand decomposition in the IWI-derived catalysts, no significant CO_2_ emission occurred above 250 °C, which also indicates that carbothermal reduction does not contribute to the second step of Co reduction (CoO → Co^0^). Interestingly, for both Co/CB-IWI and Co/CB-1900-IWI, hydrocarbon emission (*m*/*z* 15, CH_3_^+^) is detectable at temperatures as low as 230 °C, coinciding with the onset of CoO reduction and the emergence of metallic Co^0^. Since ligand decomposition as a source of hydrocarbon emission can be ruled out in the IWI samples, this hydrocarbon signal most likely originated from methane (CH_4_), formed via carbon support methanation (i.e., hydrogasification) at the Co/C interface. As the onset of CH_4_ emission coincides with the detection of the first traces of Co^0^, this process is most likely facilitated by hydrogen dissociation on the emerging Co^0^ phase and subsequent spillover of atomic hydrogen onto the carbon support. Similar to carbon gasification with oxygen, it is expected that surfaces sites reactive toward H chemisorption are necessary for carbon methanation.^91,92^ While this effect may also occur in the colloidal Co NP systems, its detection is obscured by overlapping signals from ligand decomposition. For the IWI samples, the CO_2_ and CH_4_ emissions resulting from carbon gasification did not directly reflect the differences in surface chemisorption site density between CB and CB-1900, likely as a consequence of the calcination step already modifying the Co/C interface. While CO_2_ emissions were nearly identical for Co/CB-IWI and Co/CB-1900-IWI, the CH_4_ emission from Co/CB-IWI accounted for only 28% of that from Co/CB-1900-IWI. This unexpected trend is likely a consequence of the preceding calcination step (250 °C, Ar), which altered the Co/C interface and complicates direct comparison with the colloidal systems, where Co nanoparticles were deposited onto pristine carbon surfaces. As evidenced by minimal Co particle growth during reduction (from 3.2 to 3.6 nm, +13%, Figure 4, S18), the Co phase of Co/CB-IWI appeared to be stabilized already prior to reduction, suggesting a significant influence of the calcination step.

The significance of the calcination step is further underlined by comparison of the XPS-derived sp^3^/sp^2^ carbon ratio of the supports, loaded/calcined catalysts and reduced/passivated catalysts (Figure S4-S7). In this context, the sp^3^/sp^2^ ratio increased by 11% for the defect-rich Co/CB-IWI sample and by 7% for the defect-poor Co/CB-1900-IWI material upon Co loading and calcination, indicating that catalyst calcination introduced new defect sites on the surfaces of both carbon supports. The extent of this surface modification during calcination seems to be likewise influenced by the initial density of chemisorption sites on the carbon supports, with defect-rich Co/CB-IWI experiencing larger changes in sp^3^/sp^2^ ratio than defect-poor Co/CB-1900-IWI. Subsequently, during reduction of Co/CB-IWI, the sp^3^/sp^2^ ratio remained essentially unchanged, indicating that the Co/C interface over CB was already stabilized during the calcination step and underwent only limited additional structural evolution during Co reduction (for Raman spectroscopy, see Figures S13, S22 and Side note S2). In contrast, the sp^3^/sp^2^ ratio of Co/CB-1900-IWI increased substantially (by 74%, from 0.28 after Co loading/calcination to 0.45 after reduction/passivation) during reduction, pointing to significant carbon surface modification in the defect-poor CB-1900 support, which correlates with the high emission of CO_2_ and CH_4_ during reduction of CB-1900-IWI. In this context, the preceding calcination step appeared to stabilize the Co phase for Co/CB-IWI, whereas for Co/CB-1900-IWI it acted as a “break-in” step that introduced defects into the initially defect-poor carbon surface, which are then available to participate in significant carbon (hydro-)­gasification during the subsequent reduction step.

### 3.5 Reduced Catalysts and Fischer–Tropsch Synthesis

In addition to STEM imaging conducted after reduction and passivation (Figures 4, S17–18), the reduced Co/CB catalysts were further characterized without air exposure using in situ EXAFS and in situ XRD. A summary of the results is presented in Table 2, while the data as well as a brief discussion of the EXAFS and XRD analyses is available in the Supporting Information, Figures S23-27, Tables S1-S2, Side Notes S3 and S4.

**Table 2:** Characterization of the Reduced Catalysts

Sample	*D* (Co^0^)^a^ [nm]	DOR^b^ [%]	CN^c^	R^d^ [Å]	Co^0^ hcp/fcc^e^
Co/CB-NP	9.5 ± 2.3	100	10.7	2.49	3.0
Co/CB-1900-NP	11.6 ± 2.2	100	10.8	2.49	12.9
Co/CB-IWI	3.6 ± 2.1	92	10.8	2.49	2.4
Co/CB-1900-IWI	8.0 ± 3.4	90	10.0	2.49	4.7

a Average Co particle diameter after reduction/passivation, obtained by STEM imaging. The Co^0^ diameter is determined by correcting for a 3 nm oxide layer.

b Degree of reduction, as determined by *in situ* XANES LCF analysis after up to 6 h at 350 °C.

c Co^0^–Co^0^ coordination number of the reduced catalysts (without air exposure), as determined by *in situ* EXAFS first shell fitting.

d Co^0^–Co^0^ scattering path length of the reduced catalysts (without air exposure), as determined by *in situ* EXAFS first shell fitting.

e Ratio of the Co^0^ hcp phase to the Co^0^ fcc phase of the reduced catalysts (without air exposure), as determined by Rietveld refinement of *in situ* XRD patterns.

Fischer–Tropsch synthesis was conducted after catalyst reduction (1 °C min^–1^ to 350 °C, 8 h hold) for a total of 80 h time on stream at 220 °C, 20 bar, and an H_2_:CO ratio of 2.1 (Table 3; Figures 7a, S28). By varying the weight hourly space velocity (WHSV) between 2.4 and 5.5 L g^1^_cat_ h^–1^, the initial CO conversion of all tested catalysts was adjusted to fall within a range of 20–30% (Figure S28a). Initial catalyst activity in terms of cobalt time yield (CTY)—defined as the rate of CO consumption normalized to the Co mass—revealed clear performance differences among the Co/CB catalysts. Co/CB-NP exhibited the highest initial CTY of 6.6 × 10^–5^ mol_CO_ g^–1^_Co_ s^–1^, which was 27% higher than that of Co/CB-1900-NP (4.8 × 10^–5^ mol_CO_ g^–1^_Co_ s^–1^). Interestingly, this trend was reversed for the IWI-prepared catalysts: Co/CB-1900-IWI showed a 44% higher initial CTY (6.4 × 10^–5^ mol_CO_ g^–1^_Co_ s^–1^) compared to Co/CB-IWI (3.6 × 10^–5^ mol_CO_ g^–1^_Co_ s^–1^, Table 3).

**Table 3:** FTS Activity and Selectivity of Co/CB Catalysts

Sample	*X* ^a^ [%]	CTY^a^ ^b^	STY^a^ ^c^ [10^–3^ s^–1^]	*S* CH_4_ ^a^, [%]	*S* C_2‑4_ ^a^, [%]	*S* C_5+_ ^a^, [%]	*X* ^d^ [%]	STY^d^ ^c^ [10^–3^ s^–1^]
Co/CB-NP	24	6.6	40	15	12	73	12	24
Co/CB-1900-NP	28	4.8	36	17	16	67	15	22
Co/CB-IWI	24	3.6	9	10	4	86	6	6
Co/CB-1900-IWI	19	6.4	33	8	3	89	10	18

a Initial, reported at 0–2 h TOS.

b Cobalt time yield, reported as 10^–5^ mol_CO_ g^–1^_Co_ s^–1^.

c Site time yield, based on average Co^0^ particle diameters as determined by STEM imaging, assuming spherical particles.

d Finally, reported at 80 h TOS.

**Figure 7:**
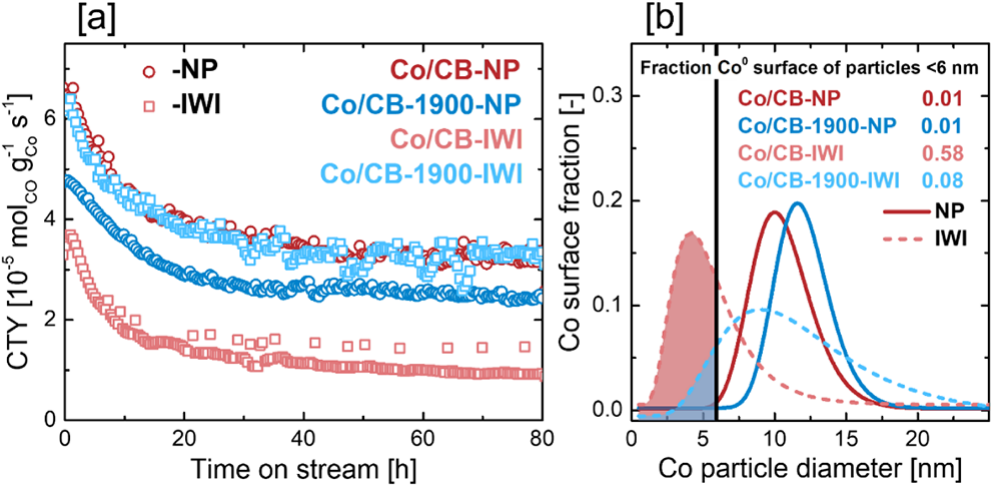
[a] Cobalt mass-based FTS activity of the Co/C catalysts over 80 h time on stream at 220 °C, 20 bar, H_2_:CO 2.1. [b] Log normal fits of the surface-normalized Co nanoparticle size distributions of the reduced/passivated Co/C catalysts. Co nanoparticles <6–8 nm are expected to show low intrinsic FTS activity due to structure sensitivity.

In terms of intrinsic activity, the site time yields (STY) of Co/CB-NP (40 × 10^–3^ s^–1^) and Co/CB-1900-NP (36 × 10^–3^ s^–1^) were found to be very similar, suggesting that the higher Co mass-based activity of Co/CB-NP is mainly a consequence of lower Co particle growth during reduction (Table 3). For the IWI samples, the intrinsic activity of Co/CB-1900-IWI (33 × 10^–3^ s^–1^) was comparable to that of the samples prepared with colloidal Co nanoparticles, however, Co/CB-IWI showed a significantly lower STY of only 9 × 10^–3^ s^–1^. The low STY of Co/CB-IWI may be assigned to the well-known structure sensitivity of Co-based FTS catalysts, where intrinsic activity decreases sharply for cobalt nanoparticles below a threshold size of 6–8 nm.^12^ Due to the high sintering resistance of Co/CB-IWI during reduction, a significant fraction of its Co nanoparticle population remains below this critical size threshold, leading to poor FTS activity (Figure 7b). In contrast, sintering during the reduction of Co/CB-1900-IWI enables most Co particles to grow beyond this threshold, resulting in substantially higher intrinsic activity. The remaining activity gap between Co/CB-1900-IWI (33 × 10^–3^ s^–1^) and the colloidal NP-based catalysts (36–40 × 10^–3^ s^–1^) may be attributed to a residual fraction of sub-6 nm Co particles, as well as to a lower degree of reduction (Table 2). Observed variations in the Co^0^ hcp/fcc ratio are difficult to interpret with regard to catalyst activity, as all catalysts are dominated by a structural complex hcp/fcc intergrowth phase, which introduces significant uncertainty to the phase ratios extracted by Rietveld refinement (see Side note S3 for further explanation).^28,90^ Product selectivity was found to be strongly influenced by the synthesis method (Table 3, Figure S28b). Catalysts prepared by IWI exhibited high C_5+_ selectivity in the range of 86–89%, whereas catalysts prepared with colloidal Co nanoparticles showed lower C_5+_ selectivities of 67–73%. This difference is likely related to variations in nanoparticle morphology and structural disorder in the resulting Co phases.^28^

Postreaction Co particle size analysis after FTS/passivation by STEM imaging revealed moderate particle growth for the catalysts based on colloidal Co NP’s (Figures 4, S18, S29). For Co/CB-NP, the average Co^0^ particle diameter increased by 15% (from 9.5 nm after reduction to 10.9 nm) after 80 h TOS, while Co/CB-1900-NP showed a similar growth of 17% (from 11.6 to 13.6 nm). Assuming that particle migration/coalescence remains the dominant sintering mechanism under FTS conditions, this translates to an average of 1.5 and 1.6 coalescence events for Co/CB-NP and Co/CB-1900-NP, respectively. In this context, Co particle growth is surprisingly moderate over 80 h time on stream, particularly when compared to the reduction step, during which 4.6 and 9.5 coalescence events were estimated for Co/CB-NP and Co/CB-1900-NP, respectively. These observations indicate that Co nanoparticles on both catalyst supports are effectively immobilized/anchored after catalyst reduction, as Co mobility appears to remain low even during exposure to high partial pressures of H_2_, CO and H_2_O at 220 °C over longer periods on stream. A similar trend was observed for the IWI-derived Co/CB-1900-IWI catalyst. Following significant Co growth during reduction (+54%, from 5.2 to 8.0 nm), only minimal additional sintering occurred during FTS, with a further increase of just 4% (to 8.3 nm) over 80 h (Figures 4, S18, S29). In contrast, Co/CB-IWI represented a significant outlier: While reduction led to only minor particle growth (+13%, from 3.2 to 3.6 nm), exposure to FTS conditions resulted in a dramatic increase in Co particle size of + 183%, from 3.6 to 10.2 nm. This unusually high degree of sintering during FTS is consistent with the large fraction of Co particles smaller than 6–8 nm, which are known to be prone to reoxidation under reaction conditions,^7,19^ whereas reoxidized Co nanoparticles have been observed to exhibit high mobility on carbon supports, resulting in rapid sintering under FTS conditions.^93^ It should be noted that the size-threshold for reoxidation may dependent on the conversion level (e.g., the partial pressure of H_2_O). As relatively low conversion levels of 20–30% were used in this study, the threshold below which reoxidation becomes viable may be lower than 6–8 nm.

### 3.6 Overall Discussion

This study set out to explore the fundamental mechanisms of cobalt nanoparticle anchoring on carbon surfaces not modified with electronegative heteroatoms, to gain insight into the “baseline” interaction between metal nanoparticles and carbon supports. To this end, two carbon black model supports were selected: a defect-rich carbon black (CB) and a defect-poor counterpart (CB-1900). These materials were found to be broadly comparable in terms of texture, morphology, and crystallinity but differed significantly in surface defect density, with CB exhibiting 20 times more chemisorption sites than CB-1900 (10.2 nm^–2^ vs 0.5 nm^–2^, Figure 2).

Using preformed, colloidal Co NP for catalyst preparation, it was observed that Co sintering on both supports occurred predominantly during the catalyst reduction step, while particle growth during subsequent FTS was minimal (Figure 4). With combined HR-SEM and HAADF-STEM imaging indicating that sintering occurred mainly via particle migration, collision, and coalescence (Figures 5, S19), the difference in Co particle growth during reduction and subsequent FTS implied that anchoring—i.e., immobilization of Co nanoparticles on the carbon surface—took place primarily during reduction for both supports. However, although the general trends in Co sintering behavior were consistent across both supports, the extent of sintering differed markedly: Co particle growth during reduction and FTS was significantly lower on the defect-rich CB than on CB-1900, indicating that chemisorption sites, which were far more abundant on CB, play a key role in anchoring Co nanoparticles and limiting their mobility.

Combined in situ XRD/XAS measurements, with mass spectrometry monitoring of the off-gas, showed that for both Co/CB-NP and Co/CB-1900-NP, Co nanoparticle growth was confined to a specific temperature and time window during catalyst reduction (Figure 6). Co nanoparticle growth began above ∼200 °C, coinciding with the Co_3_O_4_ → CoO transition, and largely ceased once the conversion of CoO to Co^0^ was completed. Within this window of Co particle growth, CO_2_ emission maxima were detected at around 225 °C for both Co/CB-NP and Co/CB-1900-NP, which were not consistent with ligand decomposition or the desorption of native carbon surface oxides. Interestingly, the CO_2_ emission maxima coincided with the first reduction step of Co_3_O_4_ to CoO and was thus attributed to carbothermal reduction of Co_3_O_4_. In this context, O species are transferred from the Co_3_O_4_ phase to defect sites susceptible to O chemisorption on the carbon support and are subsequently released as CO_2_, whereas the density of those reactive chemisorption sites at the Co/C interface being an important factor of influence. The significant difference in the density of surface sites reactive toward oxygen chemisorption between CB and CB-1900 (1.6 nm^–2^ vs 0.2 nm^–2^, Figure 2c–e), was clearly reflected in the extent of observed CO_2_ emission. In this sense, the significantly higher CO_2_ release over Co/CB-NP clearly indicated that defect-rich CB participates to a much greater extent in the carbothermal reduction of Co_3_O_4_ than defect-poor CB-1900.

We hypothesize that this participation in carbothermal reduction plays a direct role in anchoring Co nanoparticles on carbon surfaces. The carbothermal reduction of CoO_*x*_ species leads to localized carbon gasification at the Co/C interface. It is established in the literature that carbon gasification produces highly reactive, unsaturated surface sites (radicals, carbenes/carbynes) commonly referred to as “dangling bonds”, which arise from the breakage of covalent C–C and C–O bonds during the desorption of CO and CO_2_ surface complexes.^63,64,94,95^ We hypothesize that these freshly generated reactive sites, formed directly at the Co/C interface during the reduction of Co_3_O_4_, act as strong anchoring points for cobalt nanoparticles. In this context, cobalt anchoring at such sites may for example be rationalized by the ability of Co to form stable Co–carbene coordination complexes, a binding motif broadly exploited in Co complexes applied in homogeneous catalysis.^96^ Accordingly, the higher density of oxygen chemisorption sites on CB enabled more extensive carbon gasification compared to CB-1900, resulting in the formation of a larger number of reactive binding sites during the Co_3_O_4_ reduction step. This, in turn, promoted more effective anchoring of Co nanoparticles on Co/CB-NP, reduced their mobility, and ultimately lead to lower Co particle growth. Analysis of the XPS-derived sp^3^/sp^2^ carbon ratio supports this hypothesis—while a significant increase of the sp^3^/sp^2^ ratio suggested the formation of new defects for Co/CB-NP upon catalyst reduction, no changes could be detected for Co/CB-1900-NP.

Considering the catalysts prepared by incipient wetness impregnation, Co/CB-1900-IWI showed Co sintering behavior comparable to its colloidal counterpart, with significant sintering during reduction and negligible particle growth during FTS suggesting that anchoring again occurred during the reduction step (Figure 4). In situ XRD/XAS analysis indicated a similar sintering window as in the colloidal systems, and CO_2_ emission during the Co_3_O_4_ → CoO transition indicated that carbothermal reduction also played a role for the IWI-derived catalysts (Figure 6). In this context, the absence of ligand decomposition in IWI samples enabled clear differentiation between the two reduction steps. CO_2_ emissions were confined to the first step of Co reduction, while the second (CoO → Co^0^) proceeded without detectable CO_2_ release, implying that carbothermal reduction is only active in the first step under the applied conditions. This aligns with previous literature reports, indicating that full carbothermal reduction of Co/C catalysts typically requires higher temperatures in excess of 480 °C.^50,97^ In addition to carbon gasification by oxygen, hydrocarbon emission was detected starting around ∼230 °C for the IWI samples. In absence of ligands, this hydrocarbon release was attributed to carbon methanation (hydrogasification) at the Co/C interface. These emissions coincided with the first appearance of Co^0^ and are hypothesized to originate from dissociative hydrogen adsorption on Co followed by spillover of atomic hydrogen to the carbon surface. Due to the mechanistic similarities to carbon gasification by oxygen,^92,98^ we hypothesize that carbon methanation also produces highly reactive, unsaturated surface sites^92^ at the Co/C interface, which may contribute to cobalt nanoparticle anchoring. While this effect may occur in colloidal systems as well, it was masked by overlapping signals from ligand decomposition.

In general, the sintering behavior across IWI samples mirrored that observed in colloidal Co NP systems. Carbon surface defect density again correlated with Co nanoparticle growth, with Co/CB-IWI experiencing significantly less sintering than Co/CB-1900-IWI during reduction (Figure 4). However, unlike the colloidal samples, the IWI catalysts underwent a calcination step (250 °C, Ar), which appeared to modify the Co/C interface prior to reduction. In particular, Co/CB-IWI showed near-complete suppression of particle growth during reduction, suggesting that the Co phase was stabilized even before reduction began. Even though significant CO_2_ and CH_4_ emission was detected for both Co/CB-IWI and Co/CB-1900-IWI, suggesting that carbothermal reduction and carbon hydrogasification took place in both cases, the amount of carbon gasification did not reflect the difference in initial carbon surface chemisorption site density (Figure 6c,d). In this context, lower amounts of carbon (hydro-)­gasification were observed for Co/CB-IWI compared to Co/CB-1900-IWI, even though pristine CB offers a much higher amount of reactive chemisorption sites. XPS analysis suggested that the preceding calcination step was the reason for this unexpected behavior, as introduced additional defects on both supports. In consequence, the calcination step stabilized the Co phase for Co/CB-IWI, whereas for Co/CB-1900-IWI it acted as a “break-in” step that introduced defects into the initially defect-poor carbon surface, which are then available to participate in significant carbon (hydro-)­gasification during the subsequent reduction step.

Ultimately, this premature stabilization of the Co phase on Co/CB-IWI proved to be detrimental for FTS performance. Due to the absence of sintering during catalyst reduction, a large fraction of the Co nanoparticle population remained below the critical 6–8 nm size threshold (Figures 7b, S18), resulting in lower intrinsic activity compared to the other catalysts which experienced substantial Co sintering during reduction. Moreover, smaller Co nanoparticles (<6 nm) are prone to reoxidation during FTS, which is connected with rapid sintering under FTS conditions over carbon supports.,^7,19,93^ Despite being the most stable material during catalyst reduction, Co/CB-IWI thus fell victim to the intricacies of structure sensitivity, being least stable under reaction conditions with the average Co particle diameter growing by 183% over 80 h FTS (Figure 4). Nevertheless, CB remains a promising support for stabilizing Co nanoparticles in our view, provided that Co particle sizes exceed ∼6 nm after calcination, for instance through higher Co loadings.

In terms of carbon support design, our findings indicate that the “base line” interaction of carbon supports and cobalt nanoparticles (e.g., in absence of functionalization with electronegative heteroatoms) is primarily governed by the surface density of chemisorption sites, that facilitate anchoring via gasification-mediated mechanisms. In this sense, carbon supports with higher defect densities are more effective at stabilizing metal nanoparticles against sintering. From a practical standpoint, the density of surface chemisorption sites of a potential carbon support can be quantified through high-temperature TPD. However, simpler temperature-programmed oxidation measurements may also serve as effective relative descriptors for the ability of a carbon material to stabilize metal nanoparticles. The rate and temperature of carbon gasification by oxygen during TPO depend on the density of chemisorption sites susceptible toward O chemisorption,^80–82^ which, in turn, correlates with the ability of the carbon support to anchor Co nanoparticles. For supports that are otherwise similar in specific surface area, morphology, and crystallinity, the TPO rate maximum or onset (here: 598 °C for CB vs 708 °C for CB-1900) may thus serve as a relative descriptor of the ability of a carbon support to stabilize Co nanoparticles.

## 4 Conclusion

In this work, we attempted to establish a “baseline” for the interaction of cobalt nanoparticles with carbon supports in absence of any support functionalization with electronegative heteroatoms. Utilizing high temperature annealing we synthesized two carbon black model supports, one defect-rich and one defect-poor, that share texture, morphology, and crystallinity but differ 20-fold in surface defect density (10.2 vs 0.5 nm^–2^). Cobalt catalysts were then prepared using both incipient wetness impregnation and size-controlled colloidal Co nanoparticle deposition, enabling a direct comparison of two widely used synthesis strategies on well-defined supports.

Leveraging the uniform spherical morphology of the colloidal Co NPs, combined high-resolution SEM and HAADF-STEM imaging revealed that sintering occurred predominantly during the reduction step via nanoparticle migration, collision, and coalescence, with minimal further growth under Fischer–Tropsch synthesis conditions. This observation indicates that anchoring takes place during reduction, effectively immobilizing Co NPs before FTS.

In situ XANES/XRD combined with online mass spectrometry showed that Co phase transformations during catalyst reduction coincided with pronounced CO_2_ and CH_4_ evolution, attributed to carbothermal reduction and hydrogasification at the Co/C interface. Notably, the extent of carbon gasification appeared to scale with carbon defect density, reflecting the role of surface defects acting as intermittent chemisorption sites for O and H before release of CO_2_ and CH_4_. Overall, the defect-rich support delivered superior stabilization resulting in reduced sintering and, in most cases, higher FTS activity. In terms of FTS activity, one notable exception was the IWI catalyst supported on the defect-rich CB, which “overstabilized” Co NPs below the critical 6–8 nm threshold, resulting in diminished performance due to structure sensitivity.

We hypothesize that Co NP stabilization is directly connected to localized carbon gasification, generating reactive, unsaturated “dangling bonds” at the Co/C interface, which serve as in situ formed anchoring points for metallic Co NPs. Since this proposed gasification-mediated anchoring mechanism relies on the availability of chemisorption sites on the carbon surface, the carbon defect density is elevated to a practical descriptor of a support’s capability to anchor Co NP. Easily measured by TPD or TPO, defect density can therefore guide the selection and design of carbon supports. Optimizing defect site density may thus offers a route to stabilize Co nanoparticles on carbon supports without the sacrifice in FTS activity often associated with highly interacting catalyst supports.

## Supplementary Material



## Notes

Procedure for the determination of carbon interlayer spacing and crystallite size; further characterization of the carbon black supports: SEM imaging, XRD patterns, analysis of Raman spectra, TPO mass loss curves, TPD mass loss curves, XPS; further characterization of the pristine catalysts: further HAADF-STEM imaging, Co particle size distributions, XPS, Raman spectroscopy, EXAFS and XRD analysis including Rietveld refinement; characterization of catalyst reducibility via H_2_-TPR and in situ XANES, as well as discussion of those results; in situ XRD analysis during catalyst reduction; further analysis of the reduced catalysts: HR-SEM and HAADF-STEM imaging, ex situ XPS and Raman spectroscopy, in situ XRD including Rietveld refinement and discussion, in situ EXAFS with first-shell fitting and discussion; further information on Fischer–Tropsch synthesis: CO conversion and C_5+_ selectivity over 80 h TOS. HAADF-STEM imaging of spent catalysts (PDF)
