# Supplementary material for: Shaping and Stabilizing the Active Phase: The Role of Carbon Surface Defects in Carbon-Supported Co Fischer–Tropsch Synthesis Catalysts
Source: ACS Catal. 2025 Dec 19;16(1):446–63. doi: 10.1021/acscatal.5c06572 (PMC12772119; doi:10.1021/acscatal.5c06572)
Supplement: Supplementary file 1 [file cs5c06572_si_001.pdf]

Shaping and Stabilizing the Active Phase: The Role of Carbon Surface Defects in Carbon-Supported Co Fischer-Tropsch Synthesis Catalysts

Supplementary Information

Felix Herold[a,*], Mei Ju A. Goemans[a], Pierre Cautaerts[b], Bastian J. M. Etzold[b], Magnus Rønning[a,*]

[a]Norwegian University of Science and Technology, Department of Chemical Engineering, Trondheim 7491, Norway

[b]Friedrich-Alexander-Universität Erlangen-Nürnberg, Institute for Power-to-X Technologies, 90762 Fürth, Germany

**Corresponding Authors*: F. Herold, felix.herold@ntnu.no; M. Rønning, magnus.ronning@ntnu.no

1. EXPERIMENTAL

1.1 Determination of carbon interlayer spacing and crystallite size

Carbon interlayer spacing (d-spacing) was extracted from XRD patterns. In this context, the position of the intensity maximum of the (002) reflection was used to calculate the d-spacing via the Bragg equation. The crystallite size perpendicular to the graphene planes (e. g. the

stacking height) L_c was determined using the Scherrer equation, utilizing the full width at half maximum (FWHM) of the (002) reflection as well as a shape factor K_c of 0.89.

$$L_c = \frac{K_c \cdot \lambda}{FWHM \cdot \cos(\theta)}$$

The in-plane crystallite size L_a was extracted from Raman spectroscopy, using the half-width at half maximum of the G-band to employ a correlation proposed by Mallet-Ladeira for crystallite sizes below 10 nm[1]:

$$HWHM_G = \Gamma_G[cm^{-1}] = (68 \pm 4) - (5.2 \pm 0.5)L_a$$

1.2 X-ray photoelectron spectroscopy

A Kratos Analytical Axis Ultra DLD spectrometer was employed, using monochromatic Al Kα irradiation (1486.6 eV) operating the anode at 10 kV with an aperture of 700 x 300 µm. For deconvolution of the C 1s region, pseudo-Voigt profiles were utilized and Shirley background subtraction was performed prior fitting. Deconvolution of the C1s region was performed adapting procedures of Kundu *et al.* [2] and Lesiak *et al.* [3], assuming the contribution of sp^2-carbon at 284.5 ± 0.1 eV, sp^3-carbon at 285.2 ± 0.1 eV, carbon bound to oxygen by a single bond (C-O) at 286.0 ± 0.1 eV, carbon bound to oxygen by a double bond (C=O) at 287.3 ± 0.1 eV and the carboxylic acid derivates (O-C=O) at 289.1 ± 0.1 eV. The $\pi \rightarrow \pi^*$ transition satellite that occurs around 290.5 eV was found to be negligible in all samples. The full width at half maximum (FWHM) was restricted to 0.8 ± 0.2 eV. Shape factors (ratio of Lorentzian/Gaussian functions) were kept equal for all functions during the fit and were usually close to 0.5.

2. RESULTS

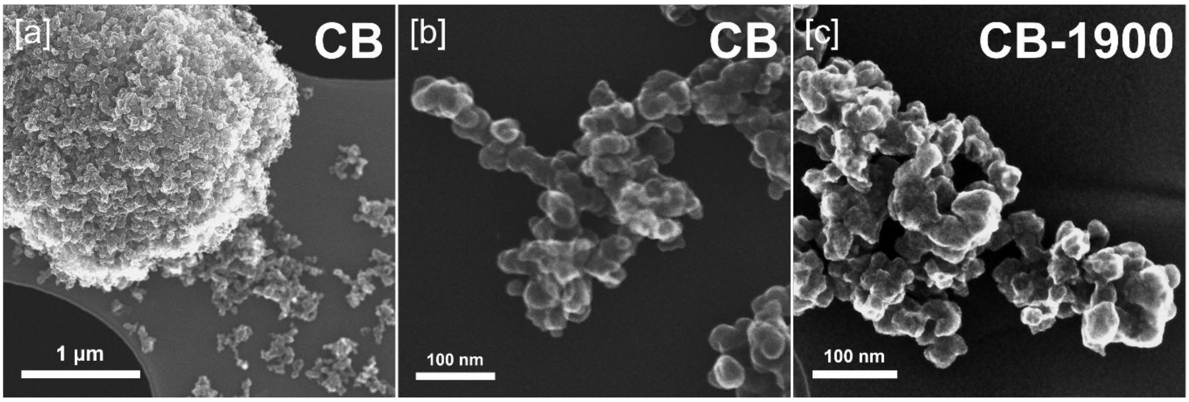

Figure S1. SEM micrographs of [a, b] CB and [c] CB-1900.

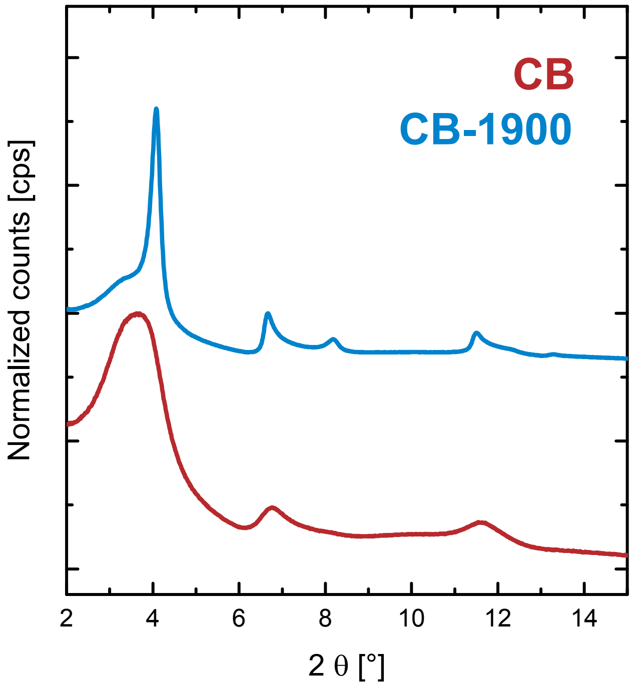

Figure S2. XRD patterns of CB and CB-1900.

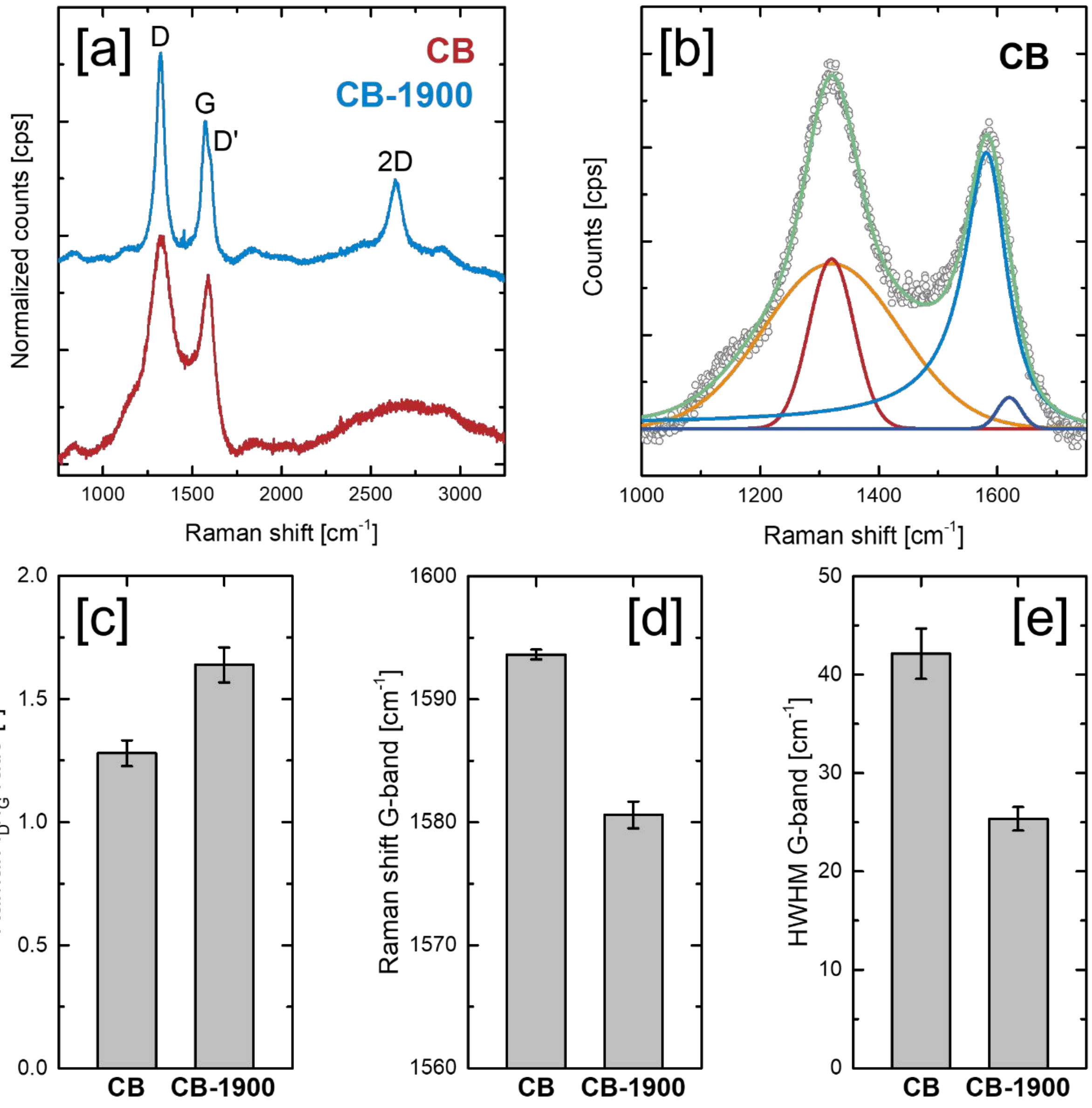

Figure S3. [a] Representative Raman spectra of CB and CB-1900. [b] Example for fitting of the Raman spectra following a procedure of Mallet-Ladeira et al.[1] Quantitative analysis of Raman spectra, in terms of [c] Raman I_D/I_G ratio, [d] Raman shift of the G-band and [e] half-width at half maximum (HWHM) of the G-band.

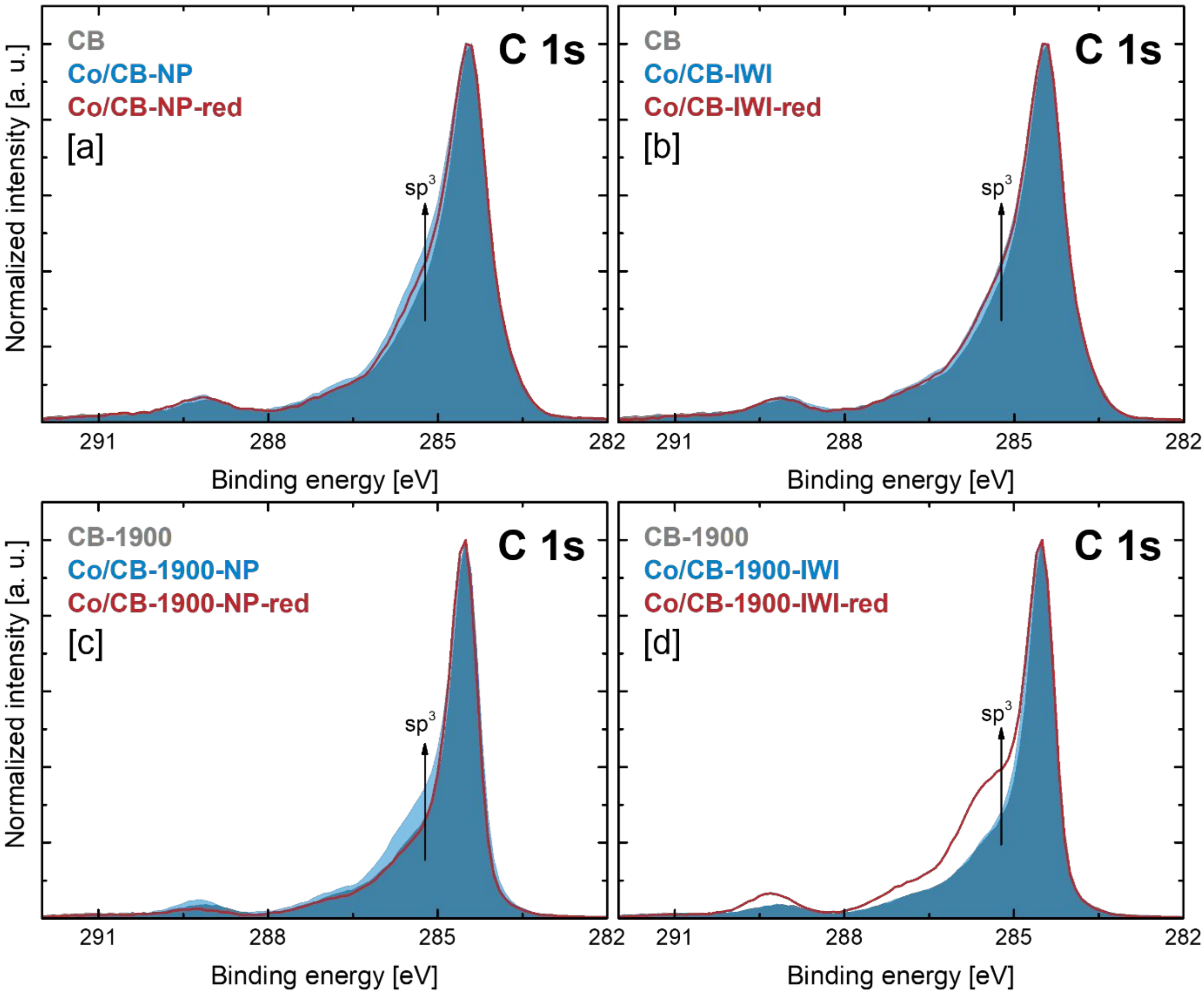

Figure S4. Baseline corrected and normalized XPS C 1s spectra for [a] Co/CB-NP, [b] Co/CB-IWI, [c] Co/CB-1900-NP and [d] Co/CB-1900-IWI, comparing the pristine supports with the materials after cobalt loading and after reduction/passivation.

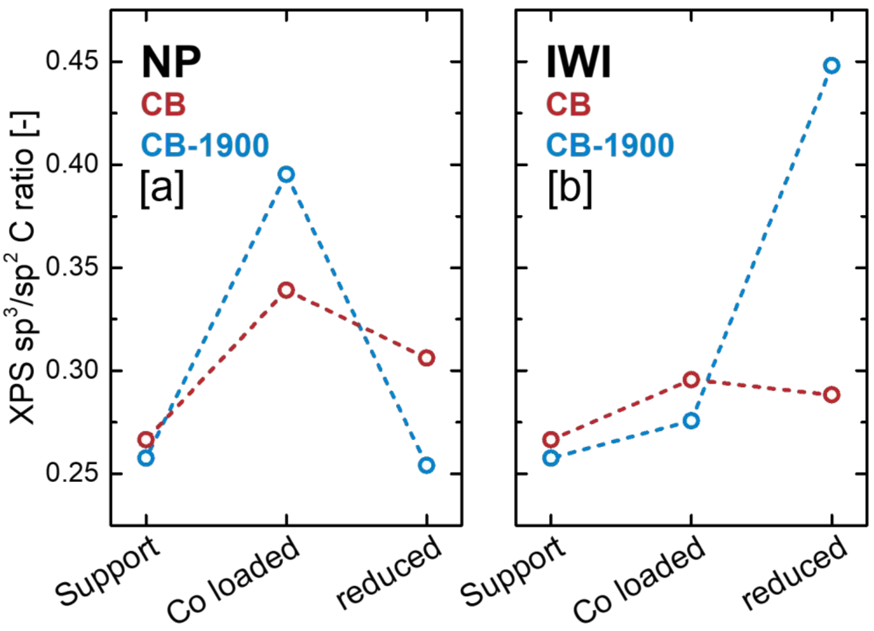

Figure S5. Carbon sp^3/sp^2 ratios extracted from the XPS C 1s contribution for [a] Co/CB-NP and Co/CB-1900-NP as well as [b] Co/CB-IWI and Co/CB-1900-IWI, comparing the pristine supports with the materials after cobalt loading and after reduction/passivation.

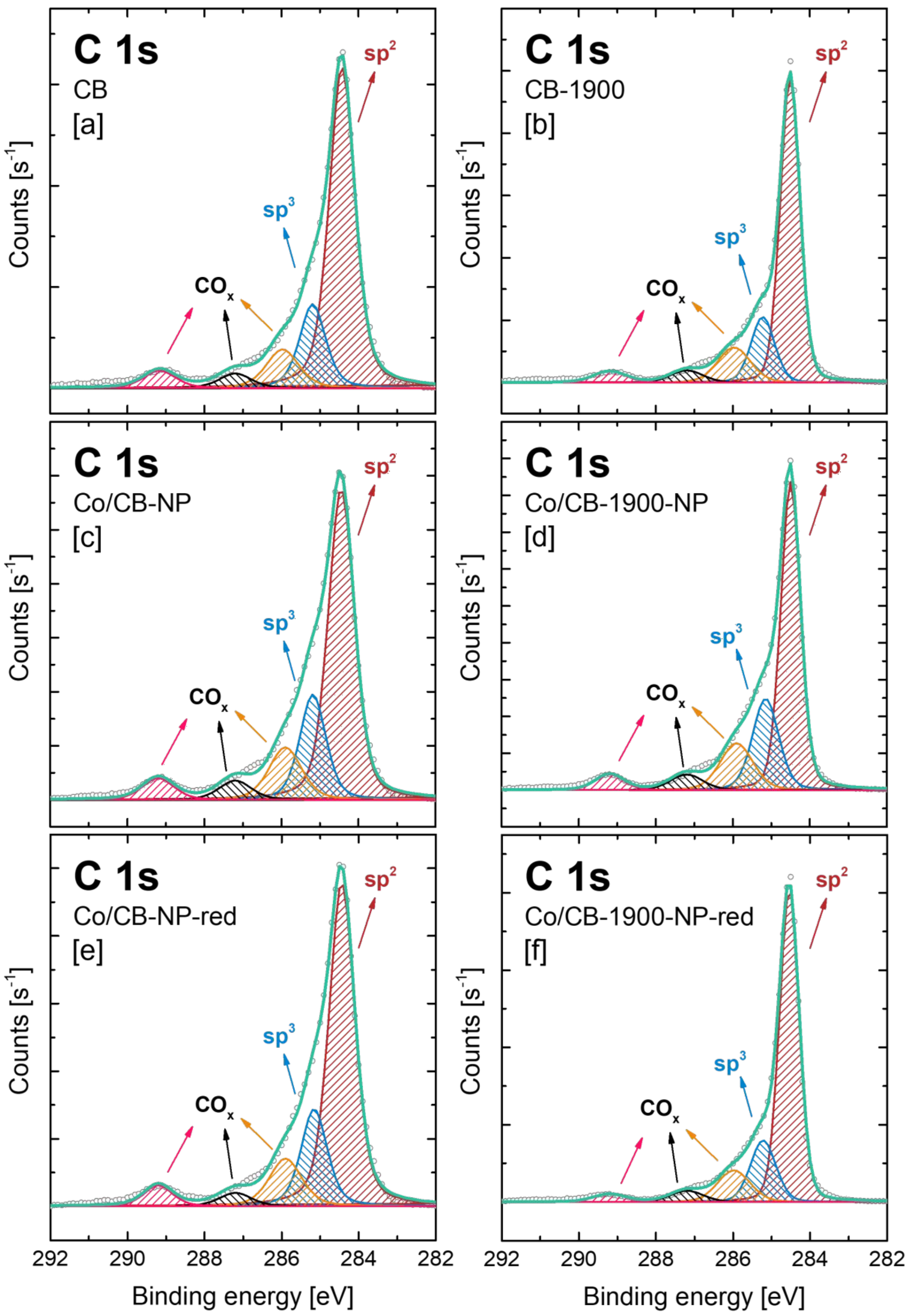

Figure S6: Analysis of the XPS C 1s contribution of [a] CB, [b] CB-1900, [c] Co/CB-NP, [d] Co/CB-1900-NP as well as [e] Co/CB-NP and [f] Co/CB-1900-NP after reduction and passivation.

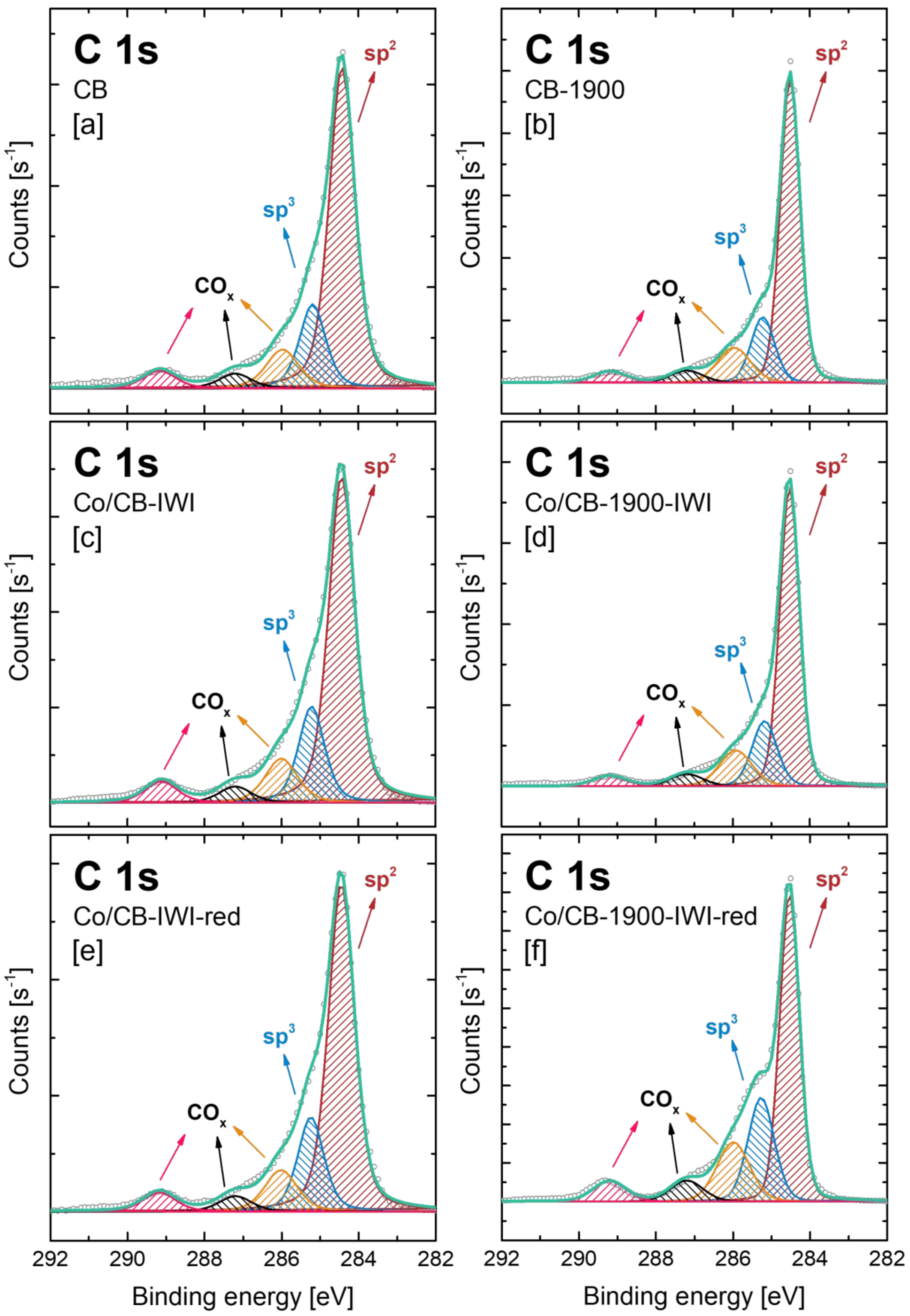

Figure S7: Analysis of the XPS C 1s contribution of [a] CB, [b] CB-1900, [c] Co/CB-IWI, [d] Co/CB-1900-IWI as well as [e] Co/CB-IWI and [f] Co/CB-1900-IWI after reduction and passivation.

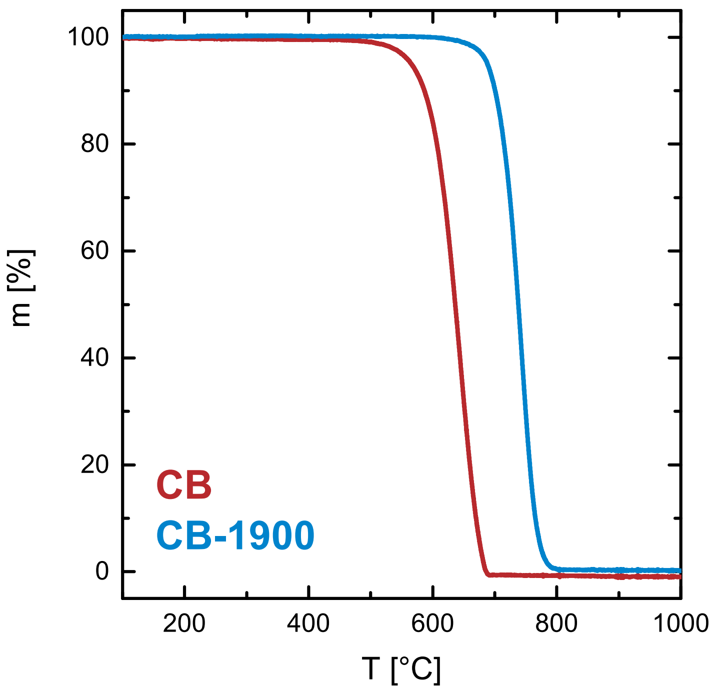

Figure S8. Mass loss curves of the supports during TPO experiments in synthetic air (10 mg carbon black,100 mL min^{-1} synthetic air, 5 °C min^{-1} to 1000 °C.)

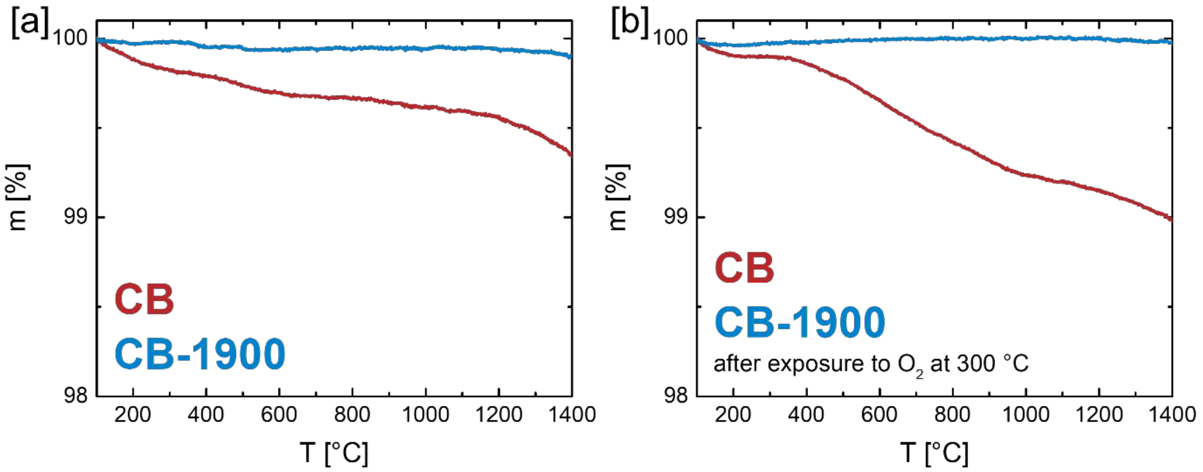

Figure S9. [a] Mass loss curves of the supports during TPD experiments in Ar (35 mg carbon black, 50 mL min^{-1} Ar, 10 °C min^{-1} to 1400 °C). [b] Mass loss curves of the supports during TPD experiments in Ar after pre-exposure to O_2 at 300 °C for 1 h (35 mg carbon black, 50 mL min^{-1} Ar, 10 °C min^{-1} to 1400 °C).

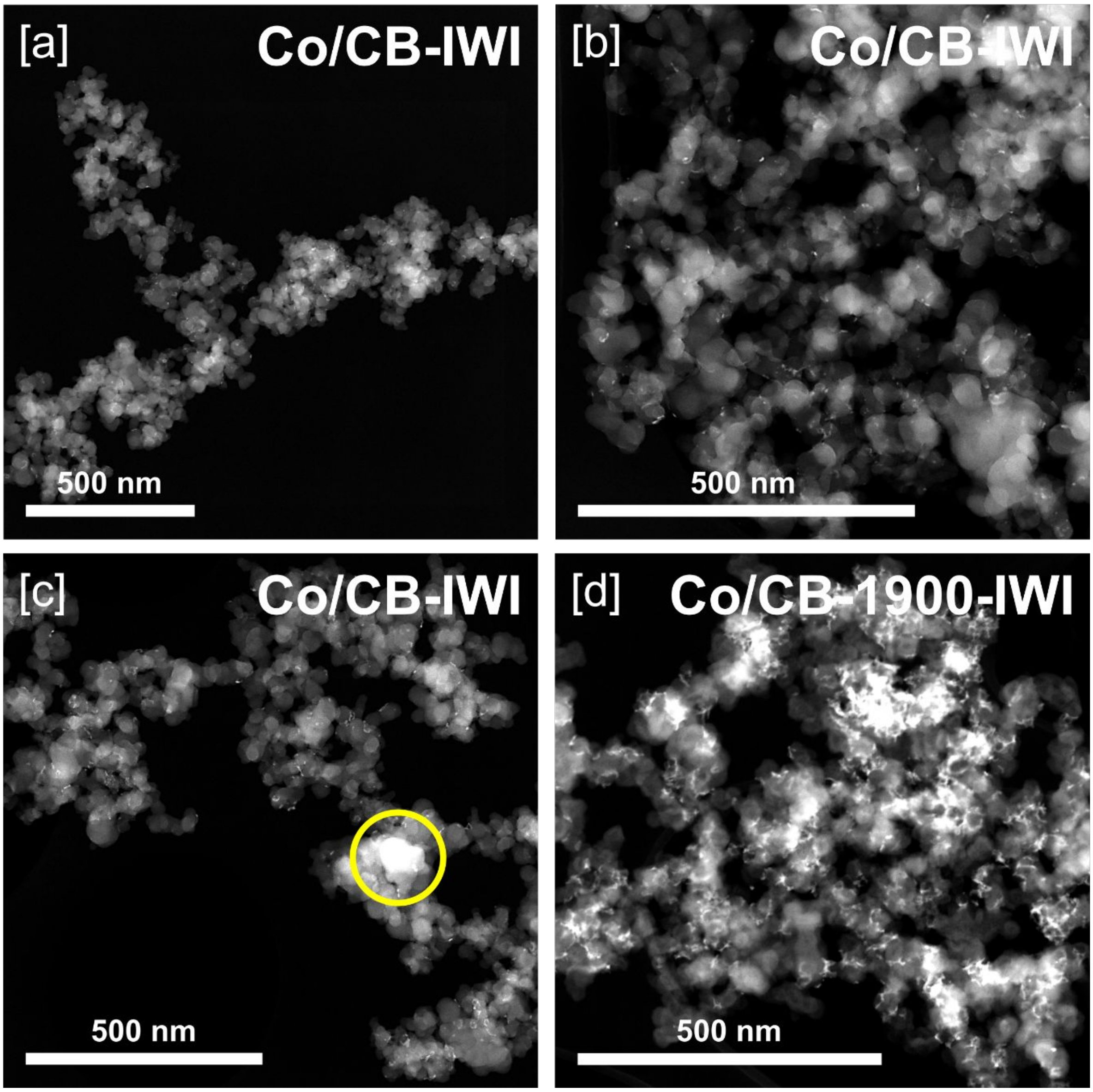

Figure S10. Low magnification HAADF-STEM overview micrographs of [a-c] Co/CB-IWI and [d] Co/CB-1900-IWI. The yellow circle in [c] marks a larger Co particle found in low abundance on Co/CB-IWI.

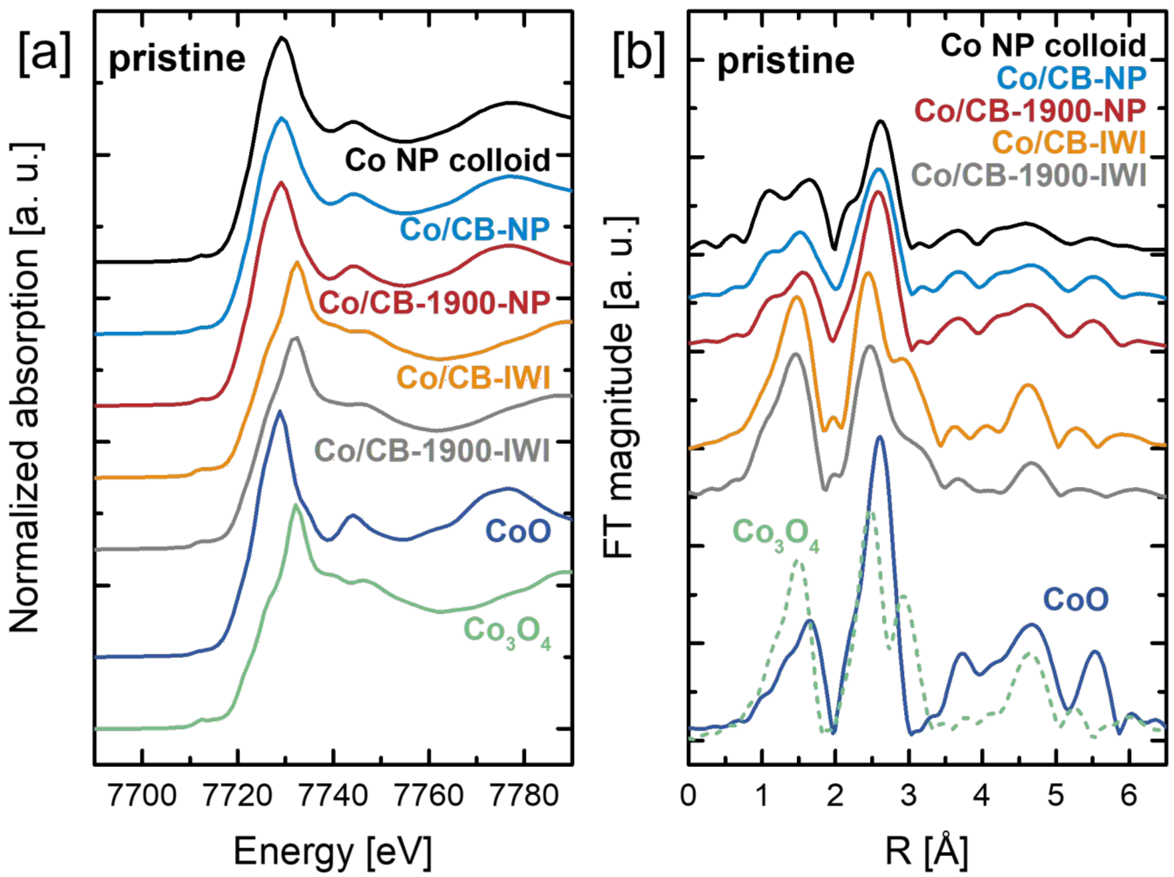

Figure S11. [a] Normalized Co K-edge XANES spectra of the Co colloid in toluene and the pristine catalysts compared to bulk Co_3O_4 and CoO standards and [b] the corresponding k^2 weighted EXAFS spectra in R space.

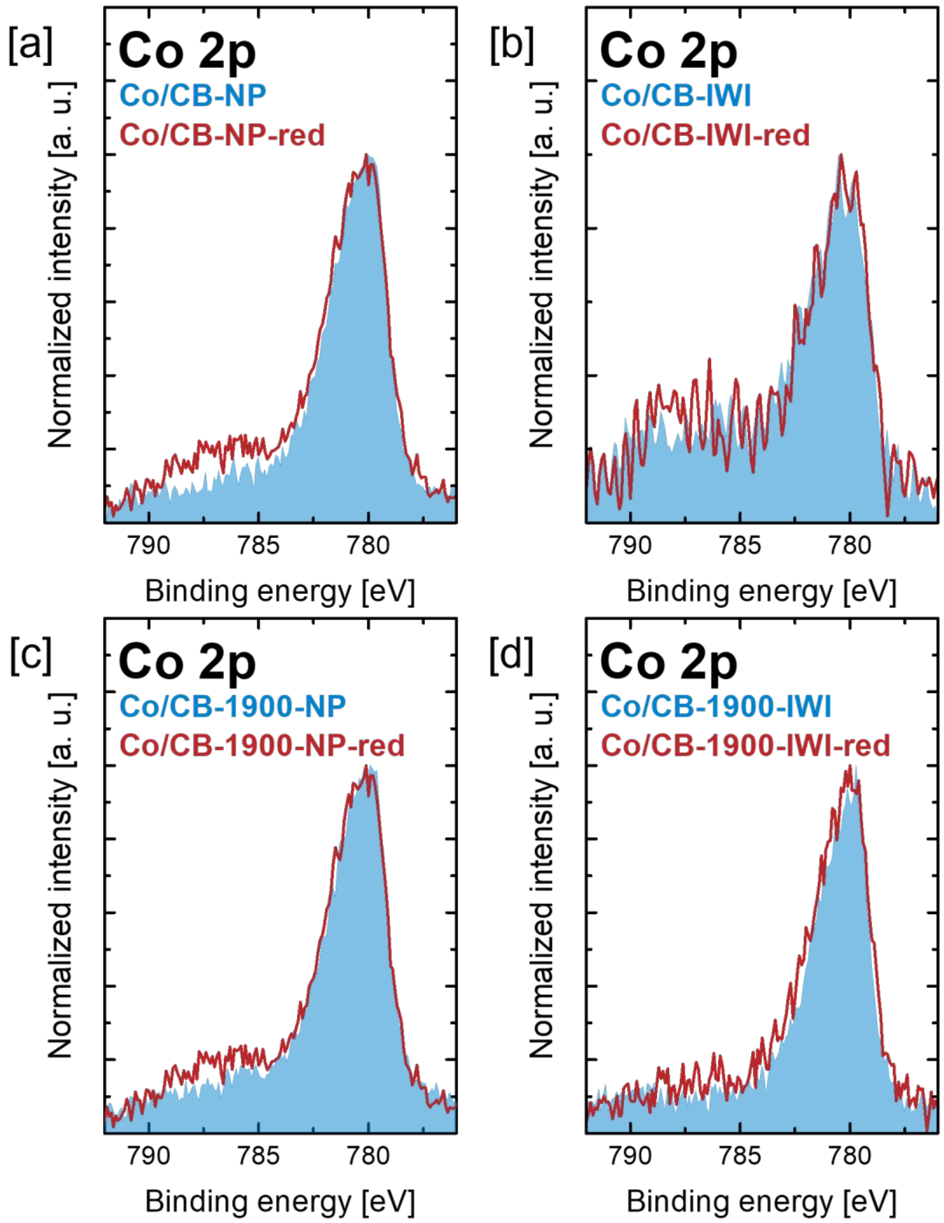

Figure S12. Baseline corrected and normalized XPS Co $2p_{3/2}$ spectra for [a] Co/CB-NP, [b] Co/CB-IWI, [c] Co/CB-1900-NP and [d] Co/CB-1900-IWI after cobalt loading and after reduction/passivation.

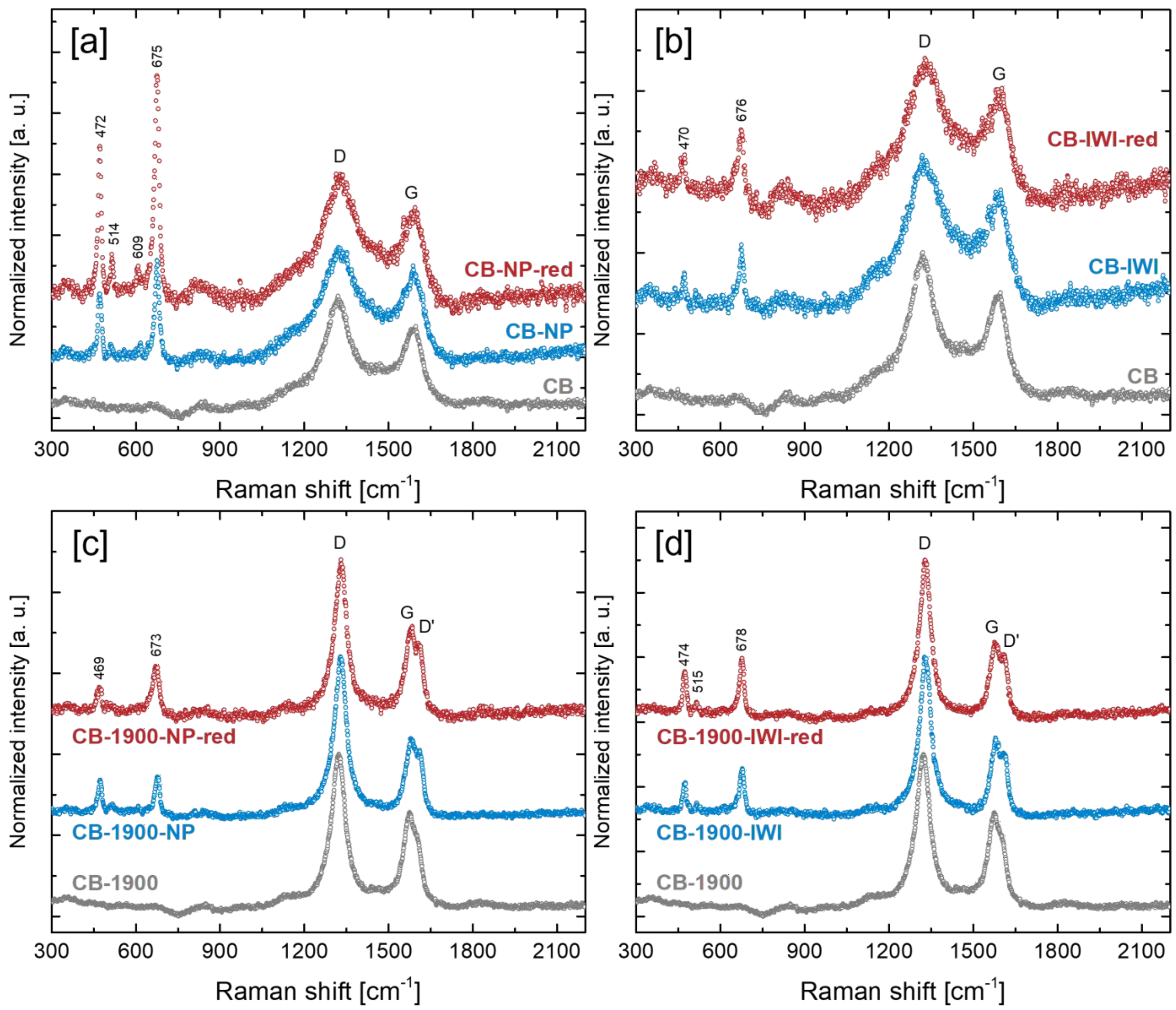

Figure S13. Comparison of representative Raman spectra of the supports, the pristine catalysts after cobalt loading and the catalysts after reduction/passivation.

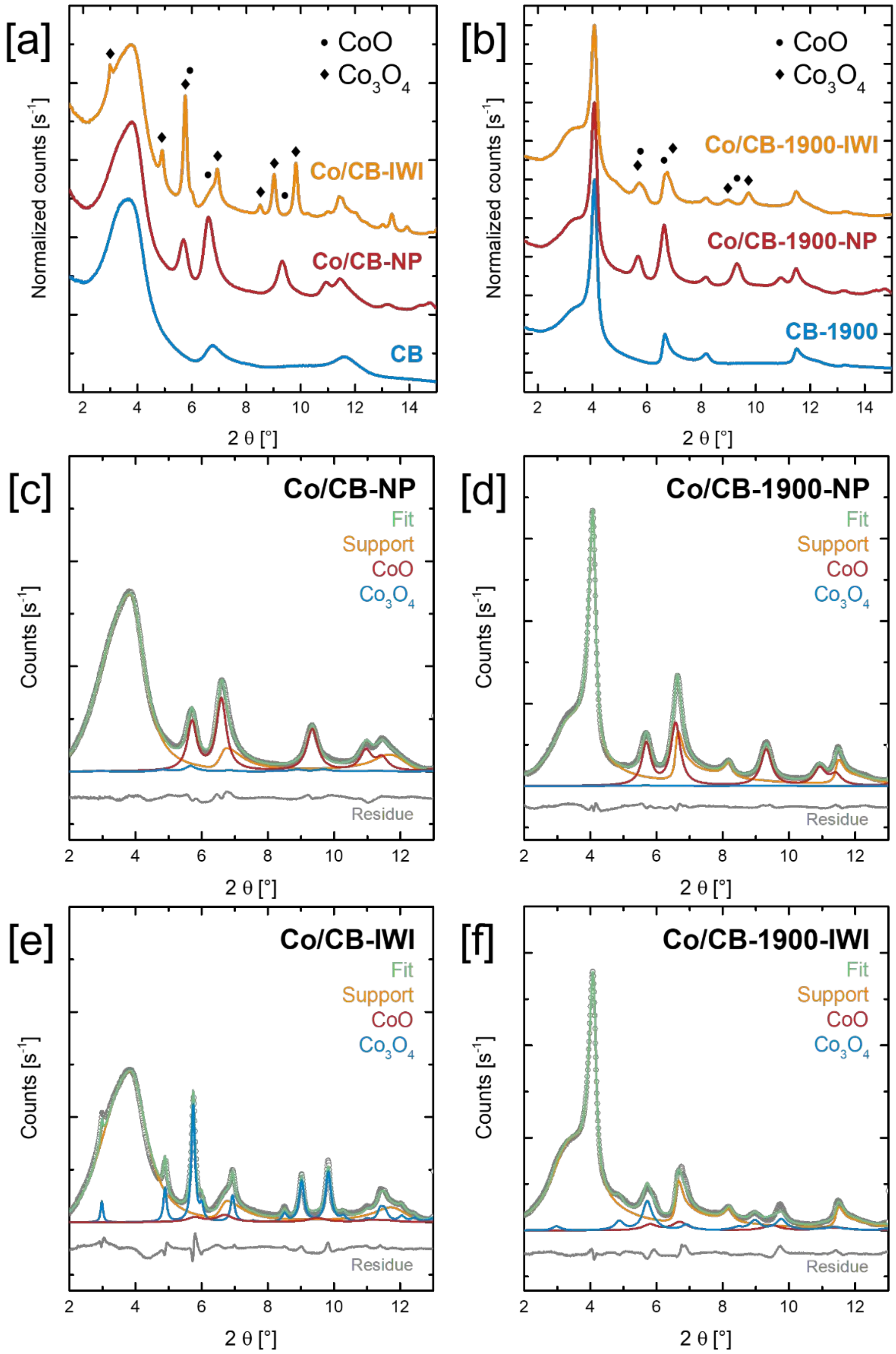

Figure S14. [a, b] XRD patterns of pristine Co/CB and Co/CB-1900 catalysts, and the corresponding Rietveld refinements of [c] Co/CB-NP, [d] Co/CB-1900-NP, [e] Co/CB-IWI and [f] Co/CB-1900-IWI.

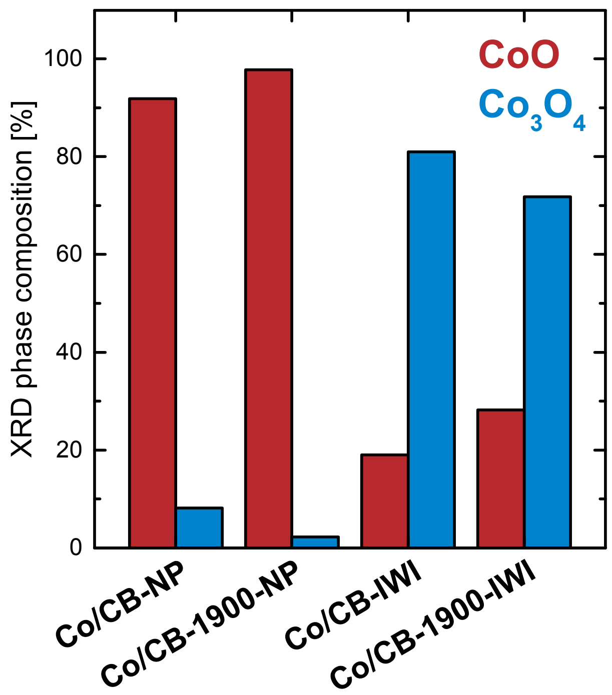

Figure S15. Co phase composition of the pristine catalysts as obtained by Rietveld refinement.

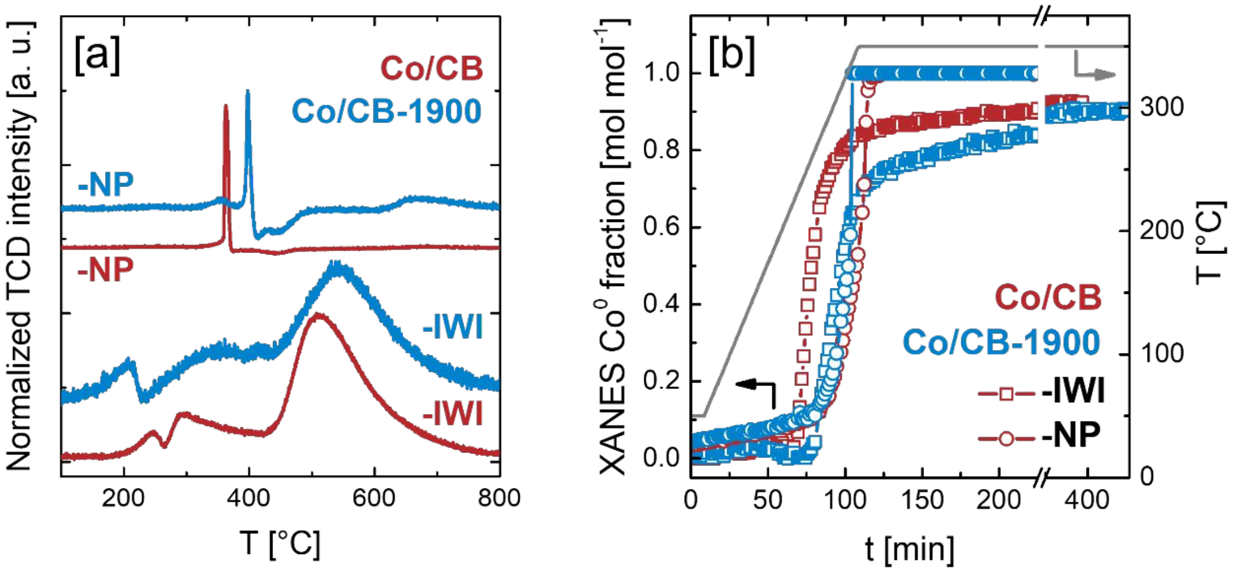

Figure S16. [a] H_2-TPR profiles of the Co/C catalysts, heating the catalyst sample in 7 vol.% H_2 in Ar at 5 °C min^{-1} to 800 °C. [b] In situ XANES-LCF derived evolution of the Co^0 fraction of the Co/C catalysts during reduction in 25 vol.% H_2 in He, 3 °C min^{-1}to 350 °C, up to 6 h hold at 350 °C.

Side note S1: Catalyst reducibility

H_2-TPR analysis revealed differences in hydrogen uptake behavior between the samples prepared with colloidal nanoparticles and those synthesized via IWI, while variations between the two carbon supports were comparatively minor. The TPR profiles of Co/CB-NP and Co/CB-1900-NP each featured a single, sharp H_2 uptake peak, characteristic of the reduction of a largely uniform cobalt phase, predominantly composed of CoO (Figure S16a).[4] Reduction rate maxima temperatures varied to some extent, between 362 °C for Co/CB-NP and 398 °C for Co/CB-1900-NP. In contrast, the catalysts prepared by IWI displayed more complex H_2-TPR profiles, consistent with their higher Co_3O_4 content. A low-temperature hydrogen uptake between 150 – 250 °C could be attributed to the reduction of Co_3O_4 to CoO, while a reduction feature between 250 and 400 °C is typically assigned to the conversion of CoO to Co^0.[5,6] Dominant H_2 uptake features > 430 °C correspond most likely to Co^0-catalyzed carbon methanation.[4,5] Co/CB-IWI appeared easier to reduce, with the maxima of the broad H_2 uptake signals for the CoO → Co^0 transition located at ~295 °C, while Co/CB-1900-IWI showed its maximum reduction rate at ~350 °C. These findings were corroborated by in situ XANES experiments, mimicking the reduction procedure performed prior to FTS catalyst testing (3 °C min^{-1} ramp to 350 °C, up to 6 h hold, 25 vol.% H_2 in He). Co/CB-NP and Co/CB-1900 were fully reducible under these conditions, with a degree of reduction (DOR) of 100 % (Figure S16b). Catalysts prepared by incipient wetness impregnation levelled out at slightly lower DOR's of 92 % for Co/CB-IWI and 90 % for Co/CB-1900-IWI with the remaining Co being present as Co^{2+}, most likely in the form of CoO.

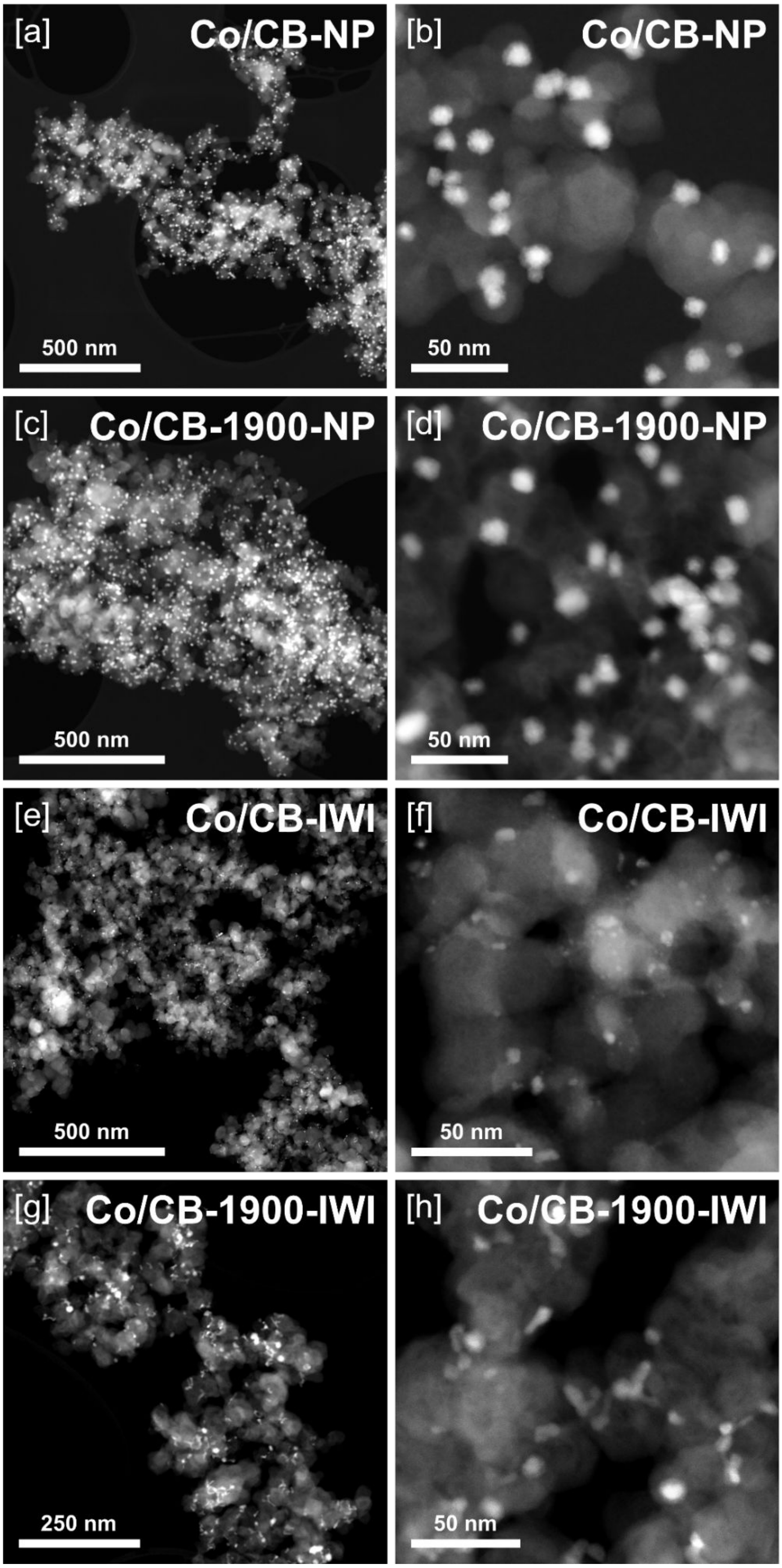

Figure S17. HAADF-STEM micrographs of the Co/C catalysts after reduction/passivation.

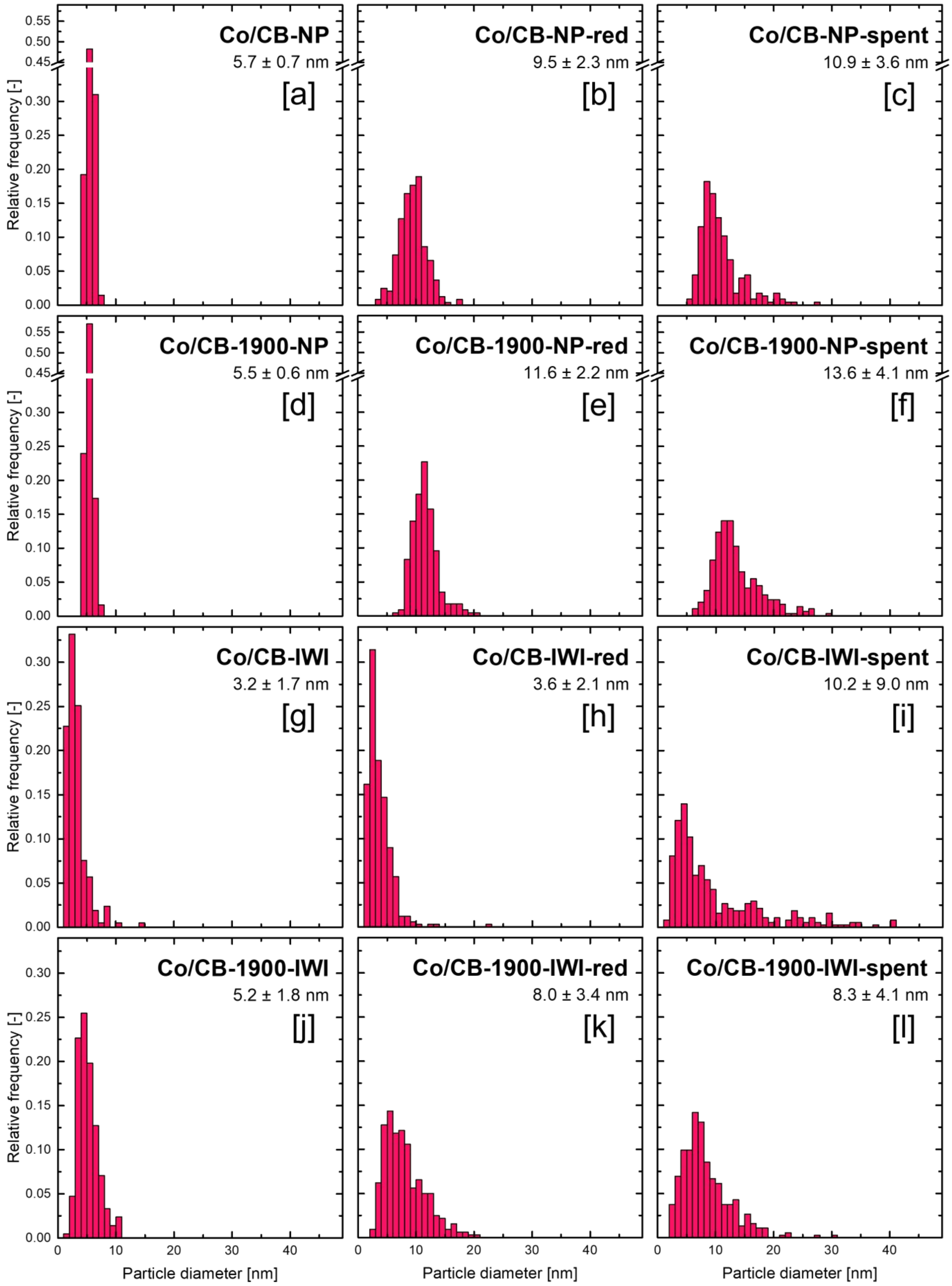

Figure S18. STEM imaging-derived Co^0 nanoparticle size distributions of [a, d, g, j] pristine, [b, e, h, k] reduced/passivated and [c, f, i, l] spent/passivated Co/C catalysts.

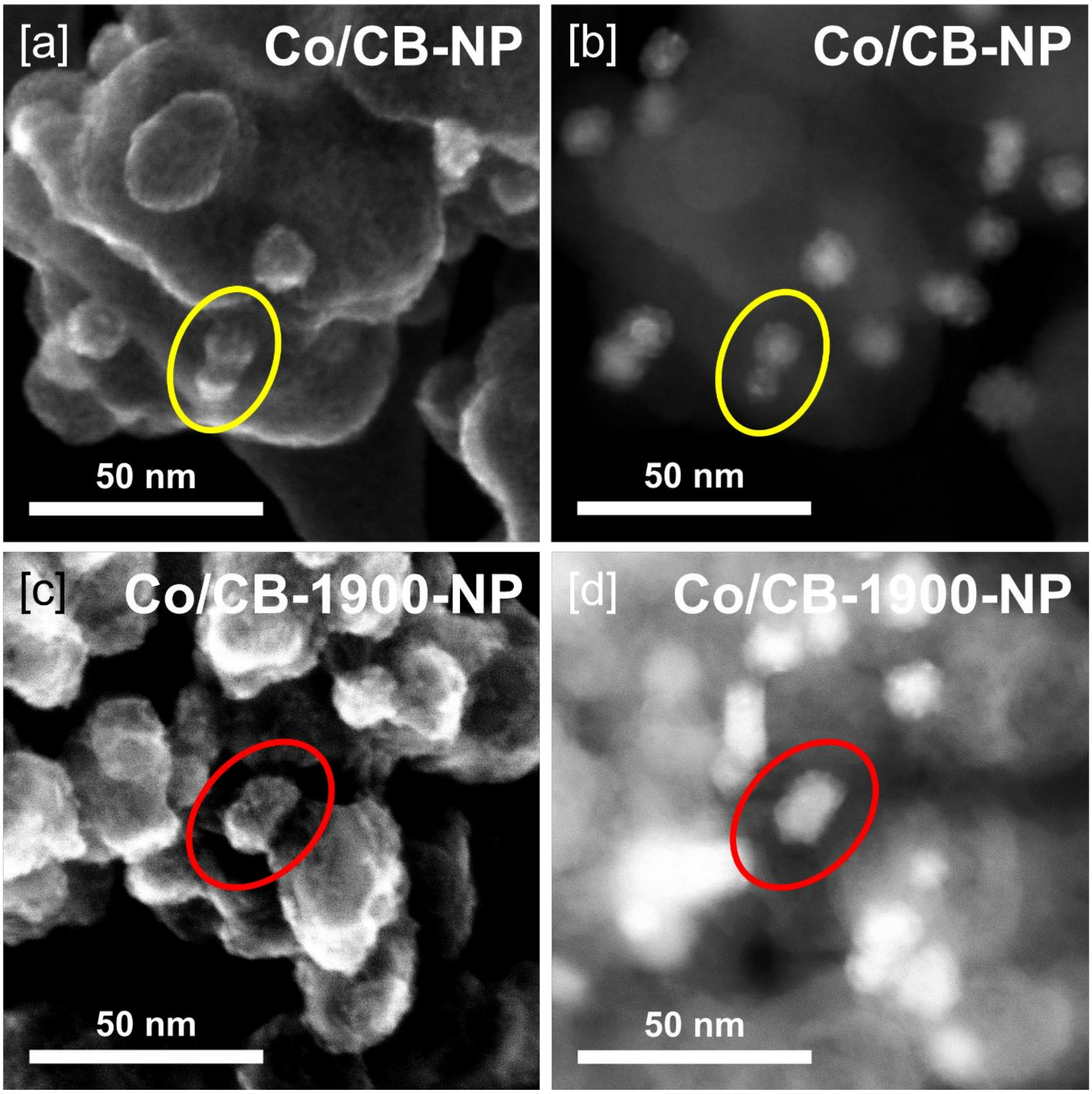

Figure S19. Combined high-resolution SEM/HAADF-STEM imaging at identical locations for reduced and passivated [a, b] Co/CB-NP and [c, d] Co/CB-1900-NP.

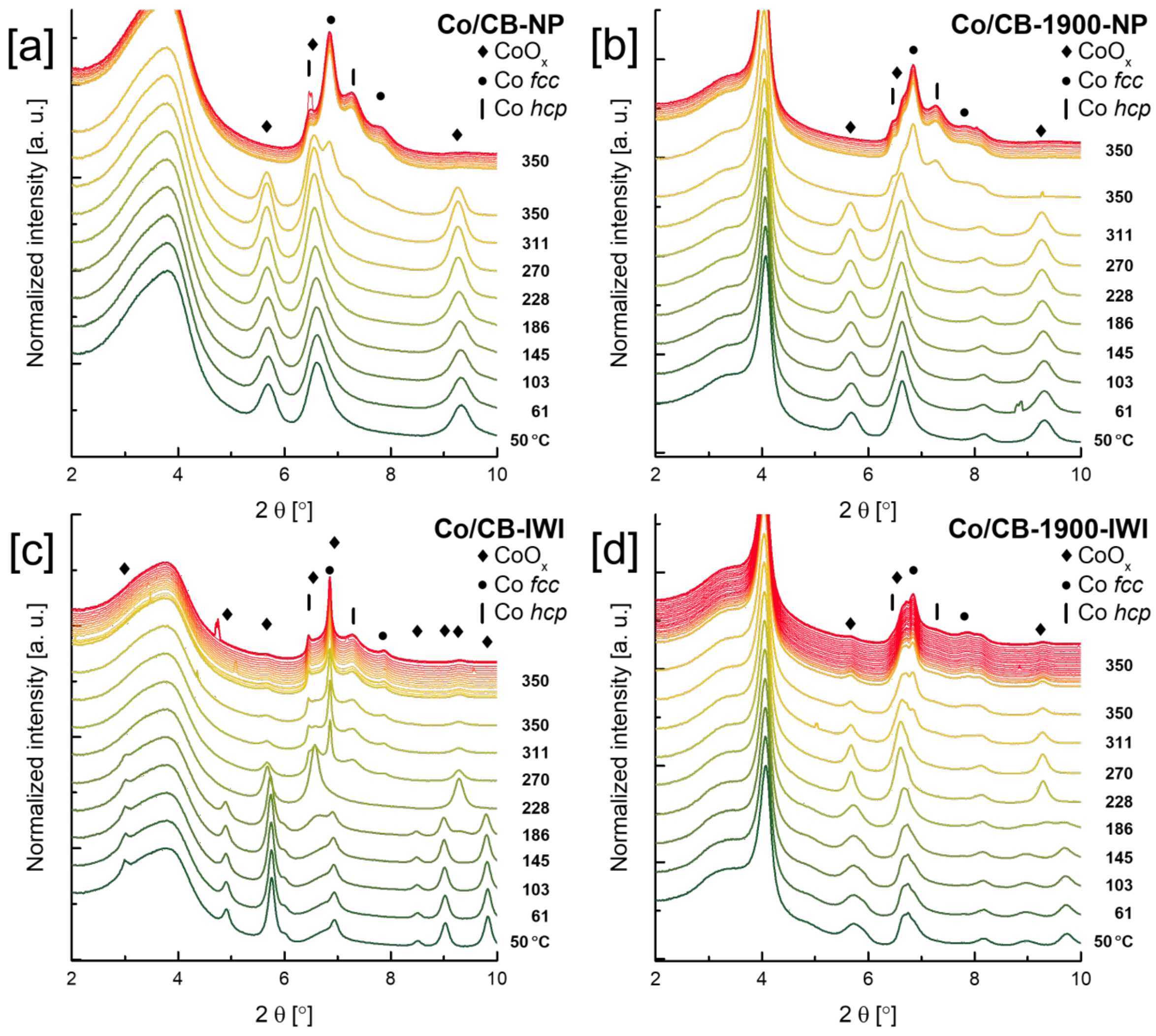

Figure S20. In situ XRD patterns during catalyst reduction of [a] Co/CB-NP, [b] Co/CB-1900-NP, [c] Co/CB-IWI and [d] Co/CB-1900-IWI, employing 25 vol.% H_2 in He, 3 °C min^{-1} to 350 °C, up to 6 h hold at 350 °C.

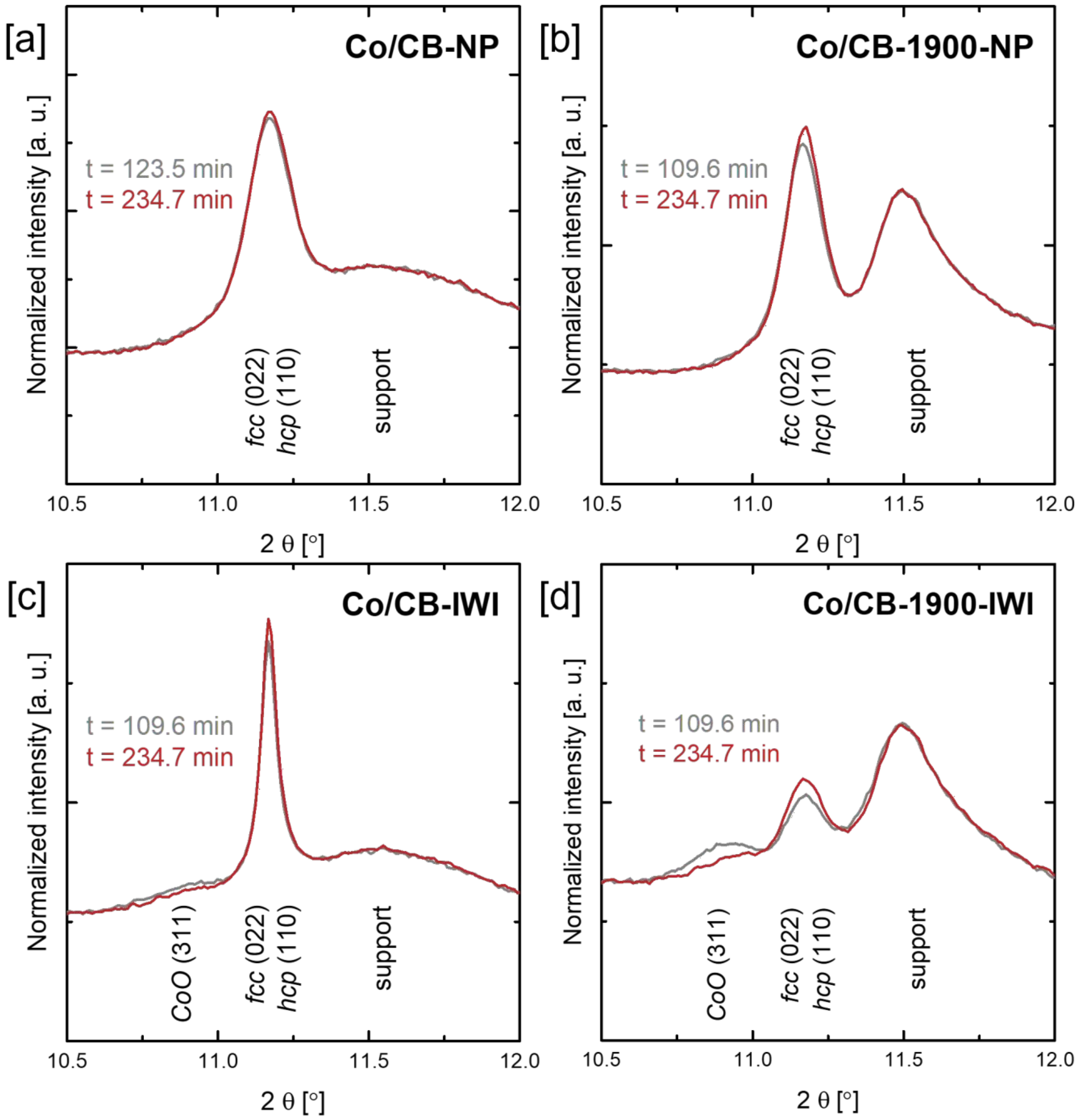

Figure S21. Comparison of the Co^0 fcc(022)/hcp(110) reflection of in situ XRD patterns of [a] Co/CB-NP, [b] Co/CB-1900-NP, [c] Co/CB-IWI and [d] Co/CB-1900-IWI, during catalyst reduction, utilized as a relative measure for Co^0 crystallite size in presence of a Co^0 hcp/fcc intergrowth phase.

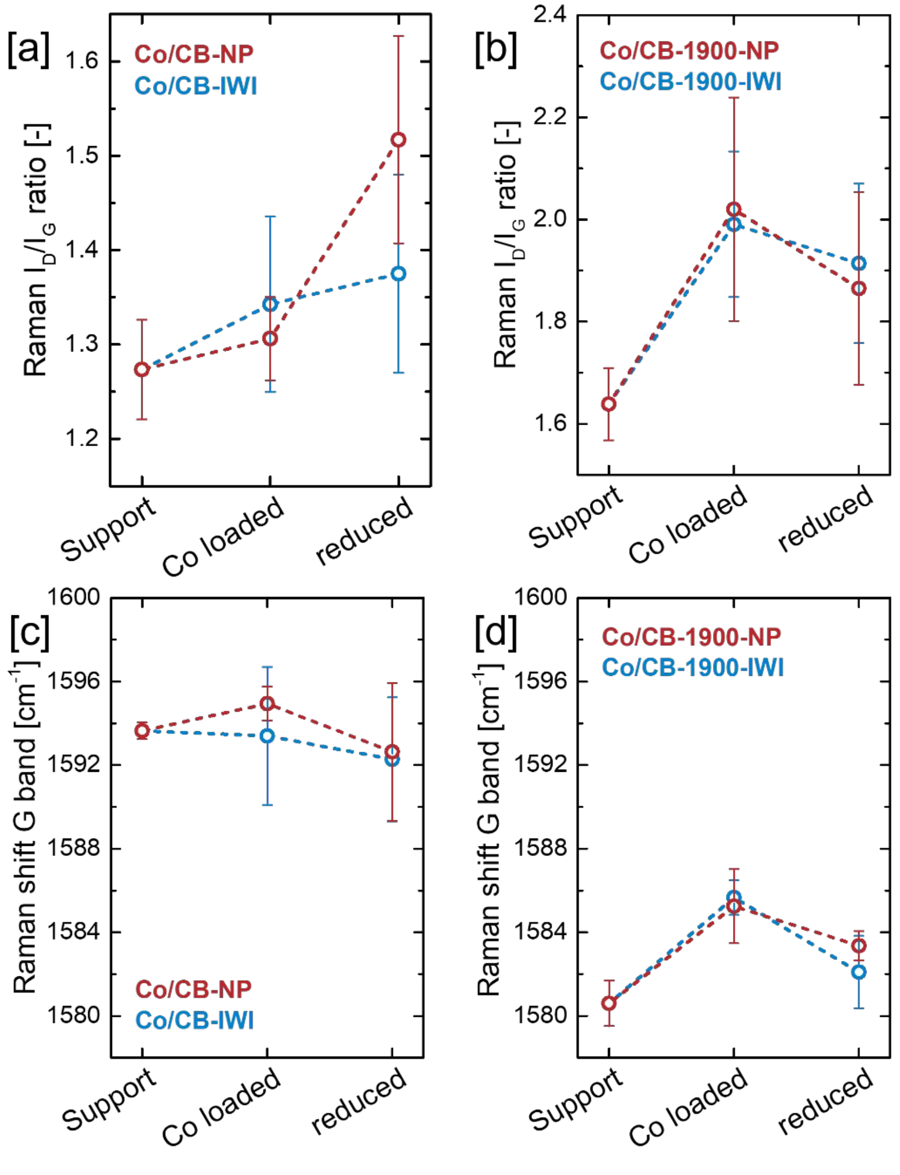

Figure S22. Comparison of the Raman ID/IG ratio of the pristine supports, the pristine catalysts after Co loading and the catalysts after reduction/passivation of [a] Co/CB-NP and Co/CB-IWI as well as [b] Co/CB-1900-NP and Co/CB-1900-IWI. Comparison of the Raman shift of the maximum of the G-band (1580 – 1600 cm^{-1}) of the pristine supports, the pristine catalysts after Co loading and the catalysts after reduction/passivation of [a] Co/CB-NP and Co/CB-IWI as well as [b] Co/CB-1900-NP and Co/CB-1900-IWI.

Side note S2: Raman spectroscopy

Upon introduction of Co, all samples, independent of preparation route and reduction/passivation state, show additional Raman features between 470 and 690 cm^{-1} (Figure S13). These bands can be assigned to vibrational modes of the spinel structure of Co_3O_4, namely the E_g (469 – 474 cm^{-1}), F_{2g} (514-515 cm^{-1} and 609 cm^{-1}) and A_{1g} (673 - 678 cm^{-1}) modes.[7] Modes attributable to CoO are not observed, however, it is known that CoO exhibits a lower Raman cross section as compared to Co_3O_4.[7] In this context, Raman spectroscopy confirms the presence of the Co_3O_4 phase in all samples, which is in agreement with XANES-LCF (Table 1) as well as the XPS results.

To follow the evolution of the carbon structure upon cobalt deposition, calcination (for the IWI samples), and reduction, we used the intensity ratio of the D and G bands (I_D/I_G), obtained from fitting the Raman spectra as described in Figure S3. This ratio is widely used in the carbon materials community as a descriptor of structural (dis-)order, as it is sensitive to changes in the amount of structural defects.[8] In terms of the I_D/I_G ratio, the catalysts supported on CB follow the same general trends observed in the sp^3/sp^2 ratios obtained from XPS (Figures S5 and S22a). After loading with colloidal Co nanoparticles, the I_D/I_G ratio changes only slightly (from 1.27 ± 0.05 to 1.30 ± 0.04), indicating that wet impregnation with colloidal Co does not induce measurable structural modification of the CB support. Upon reduction of Co/CB-NP, the I_D/I_G ratio increases to 1.52 ± 0.11, and the broader standard deviation indicates increased spatial variability within the sample, which is consistent with a decrease in structural order, for example caused by the formation of new defects. For Co/CB-IWI, the most significant change in the I_D/I_G ratio occurs during the calcination step, where both the I_D/I_G ratio and its standard deviation increase (from 1.27 ± 0.05 to 1.34 ± 0.09), indicating that cobalt loading via incipient wetness impregnation followed by calcination induces a loss of structural order and reduces sample homogeneity. During subsequent catalyst reduction, a further increase in I_D/I_G and in

the standard deviation is observed, which is albeit less pronounced (from 1.34 ± 0.09 to 1.38 ± 0.11). These observations generally align with the trends in the sp^3/sp^2 ratios obtained by XPS, albeit XPS features attributed to the oleic acid ligands are not reproduced due to the lower surface sensitivity of the confocal Raman microscopy applied here.

For the catalysts supported on CB-1900, the Raman results do not follow the trends indicated by the XPS data (Figures S5 and S22b). Upon cobalt loading, both Co/CB-1900-NP and Co/CB-1900-IWI exhibit a pronounced increase in the I_D/I_G ratio and a substantial increase in the associated standard deviation (from 1.64 ± 0.07 to 2.0 ± 0.22 for Co/CB-1900-NP and to 2.0 ± 0.14 for Co/CB-1900-IWI), suggesting a decrease in structural order together with reduced sample homogeneity. This behavior is not supported by the XPS results, which imply only minor changes in the sp^3/sp^2 ratios for both samples at this stage and therefore indicate that limited structural change of the CB-1900 support should occur during Co deposition. The presence of oleic acid ligands can be excluded as an explanation, because both Co/CB-1900-NP and Co/CB-1900-IWI show a nearly identical response, and Co/CB-1900-IWI does not contain surface ligands. Moreover, for Co/CB-NP, no comparable increase in I_D/I_G is observed relative to the pristine CB support (Figure S22a), which further indicates that ligand effects are unlikely to be responsible for the behavior observed on CB-1900. After catalyst reduction, the I_D/I_G ratios of Co/CB-1900-NP and Co/CB-1900-IWI decrease slightly. This trend is also unexpected because both the XPS results and the emission of CO_2 and CH_4 during reduction are consistent with an increase in structural disorder of the carbon surface.

In view of these observations, it appears likely that the changes in the Raman spectra of CB-1900 upon Co deposition are not primarily caused by modifications of the carbon structure. Instead, they may originate from electronic interactions between cobalt and the defect-poor carbon support. Charge transfer effects upon metal deposition on graphitic carbon materials are known in the literature[9,10] and have been reported to influence the I_D/I_G ratio[11] and, more

prominently, the frequency of the G-band (Figure S22c, d).[9,12,13] In the present case, both Co/CB-1900-NP and Co/CB-1900-IWI show an identical shift of the G-band from 1581 to 1585 cm^{-1} after Co deposition, regardless of the preparation route. This suggests that the shift arises from the presence of the metal rather than from structural changes of the carbon matrix. The shift to higher frequencies is consistent with charge transfer from cobalt to the CB-1900 support.[9] Upon reduction, the G-band shifts from 1585 to 1583 cm^{-1} for Co/CB-1900-NP and to 1582 cm^{-1} Co/CB-1900-IWI, indicating a reduction in charge transfer. This is in line with the decrease in Co/C interfacial contact area expected from the substantial nanoparticle growth observed for both materials during the reduction step (Figure 4).

In summary, the Raman data confirms the presence of Co_3O_4 in the pristine and reduced/passivated Co/C catalysts. The comparison of the Raman I_D/I_G ratio for the catalysts supported on CB before and after reduction implies formation of defects in the carbon support during catalyst calcination for the -IWI sample as well as for both -NP and -IWI samples during reduction. The changes in I_D/I_G ratio and Raman shift of the G-band for the catalysts supported on CB-1900 upon Co loading and catalyst reduction are not consistent with changes in the carbon structure, but may be explained by charge transfer effects between Co and the graphitic carbon support.

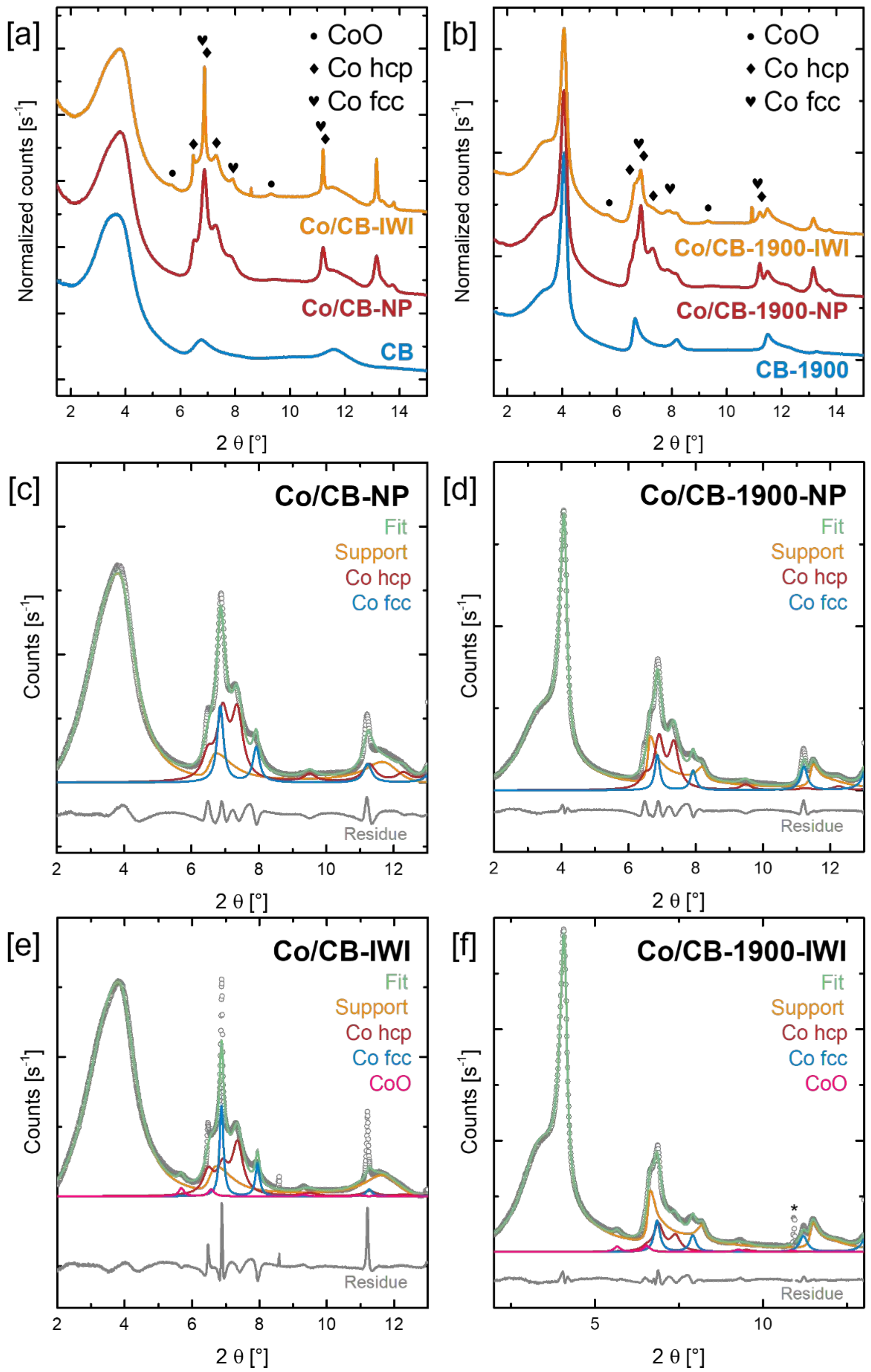

Figure S23. [a, b] In situ XRD patterns of reduced Co/CB and Co/CB-1900 catalysts, and the corresponding Rietveld refinements of [c] Co/CB-NP, [d] Co/CB-1900-NP, [e] Co/CB-IWI and [f] Co/CB-1900-IWI.

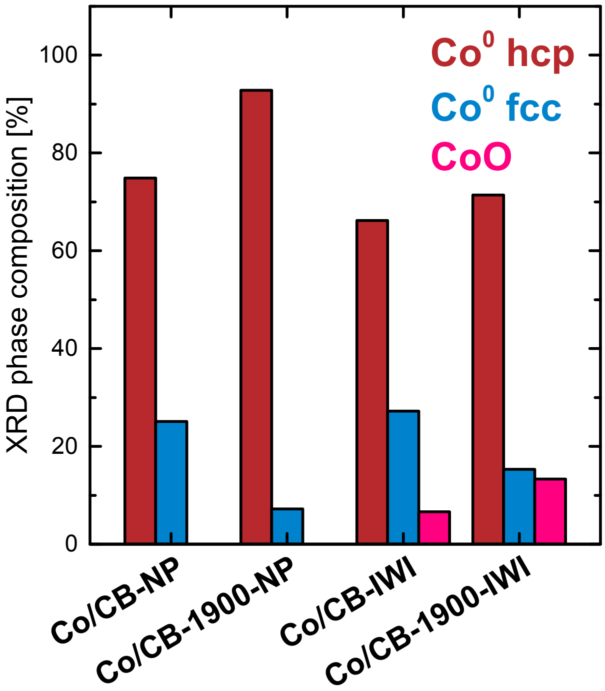

Figure S24. Co phase composition of the pristine catalysts as obtained by Rietveld refinement of in situ XRD patterns after catalyst reduction.

Table S1. Rietveld refinement of in situ XRD patterns after catalyst reduction.

**Sample**	**Co *hcp* [wt.%]**	**Co *fcc* [wt.%]**	**CoO [wt.%]**	**Co *hcp* crystallite size [nm]**	**Co *fcc* crystallite size [nm]**
**Co/CB-NP**	74.9	25.1	-	3.1	7.7
**Co/CB-1900-NP**	92.8	7.2	-	4.1	12.7
**Co/CB-IWI**	66.2	27.2	6.6	3.3	31.8
**Co/CB-1900-IWI**	71.4	15.3	13.3	3.9	8.9

Side note S3: Rietveld refinement of in situ XRD of reduced catalysts

In situ XRD analysis was performed directly after catalyst reduction, avoiding any exposure to air. For all catalysts, crystalline metallic cobalt was present in both the hexagonal close-packed (hcp) and face-centered cubic (fcc) phases, while residual CoO was detected in Co/CB-IWI and Co/CB-1900-IWI (Figures S23-24, Table S1). Rietveld refinement assuming a physical mixture of the Co hcp and fcc phases proved inadequate for all catalysts, likely due to the presence of a Co hcp/fcc intergrowth structure, which is regularly encountered in reduced supported cobalt oxide nanoparticles [4,14–16]. In support of this hypothesis, the diffraction patterns obtained matched well with simulated XRD profiles of 5 – 10 nm Co nanoparticles featuring hcp/fcc intergrowth, as modelled by Sławiński et al.[14] To improve the quality of the fit, a methodology introduced by van Deelen et al. was adapted, which was developed for a closely related Co/C catalyst system.[4] This approach involves modelling preferred orientation for Co hcp crystallites. While physically questionable, this assumption enables more accurate reproduction of the XRD patterns of the intergrowth structure, albeit at the cost of increased uncertainty in the refinement outputs. Despite this, the phase compositions derived from Rietveld refinement were in reasonable agreement with those obtained from in situ XANES-LCF analysis. In this context, Rietveld refinement yielded CoO fractions of ~7 % and ~13 % for Co/CB-IWI and Co/CB-1900-IWI (Figure S24, Table S1), respectively, while XANES analysis afforded residual CoO fractions of 8 % and 10 %, respectively (Figure S16b, Table 2). With respect to crystallite size, Co hcp domains were mostly smaller than the average particle sizes determined by STEM. This deviation is likely an artifact resulting from the preferred orientation assumption applied during refinement, as also noted by van Deelen et al.[4] In contrast, Co fcc crystallite sizes were generally consistent with the STEM-derived particle sizes. For Co/CB-IWI in particular, the larger Co fcc crystallite size aligns with the presence of a

small population of larger cobalt particles, as observed in STEM imaging (Figure S18, Table S1).

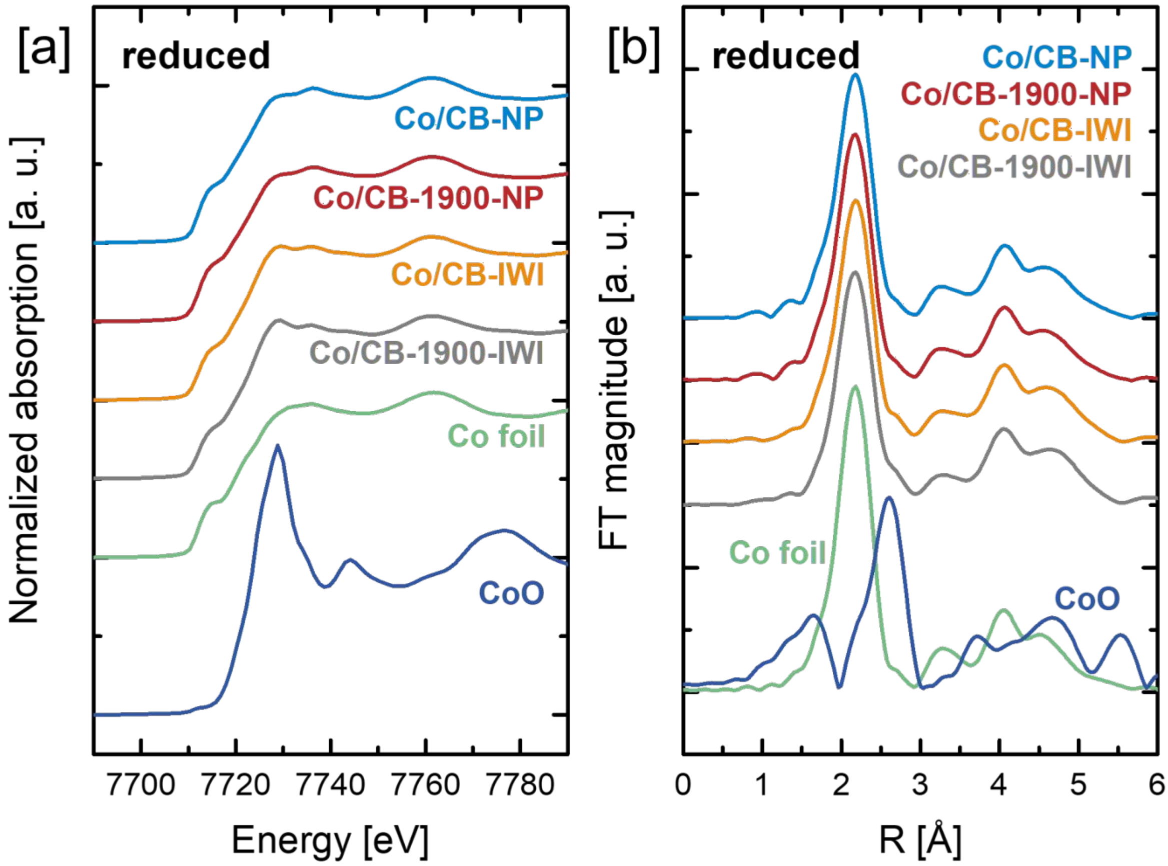

Figure S25. [a] Normalized in situ Co K-edge XANES spectra of the reduced catalysts compared to bulk Co^0 and CoO standards and [b] the corresponding k^2 weighted in situ EXAFS spectra in R space.

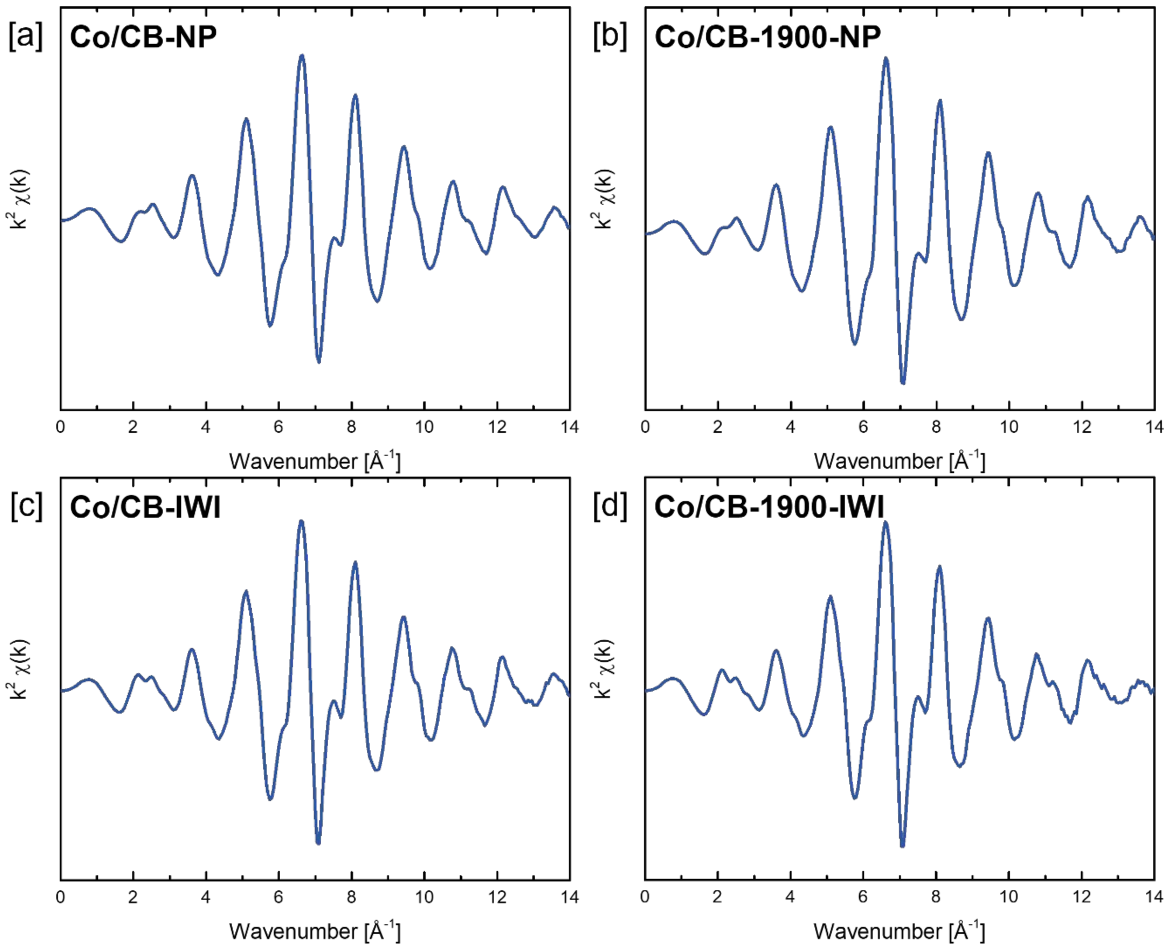

Figure S26. k^2 weighted in situ EXAFS spectra of reduced [a] Co/CB-NP, [b] Co/CB-1900-NP, [c] Co/CB-IWI, [d] Co/CB-1900-IWI.

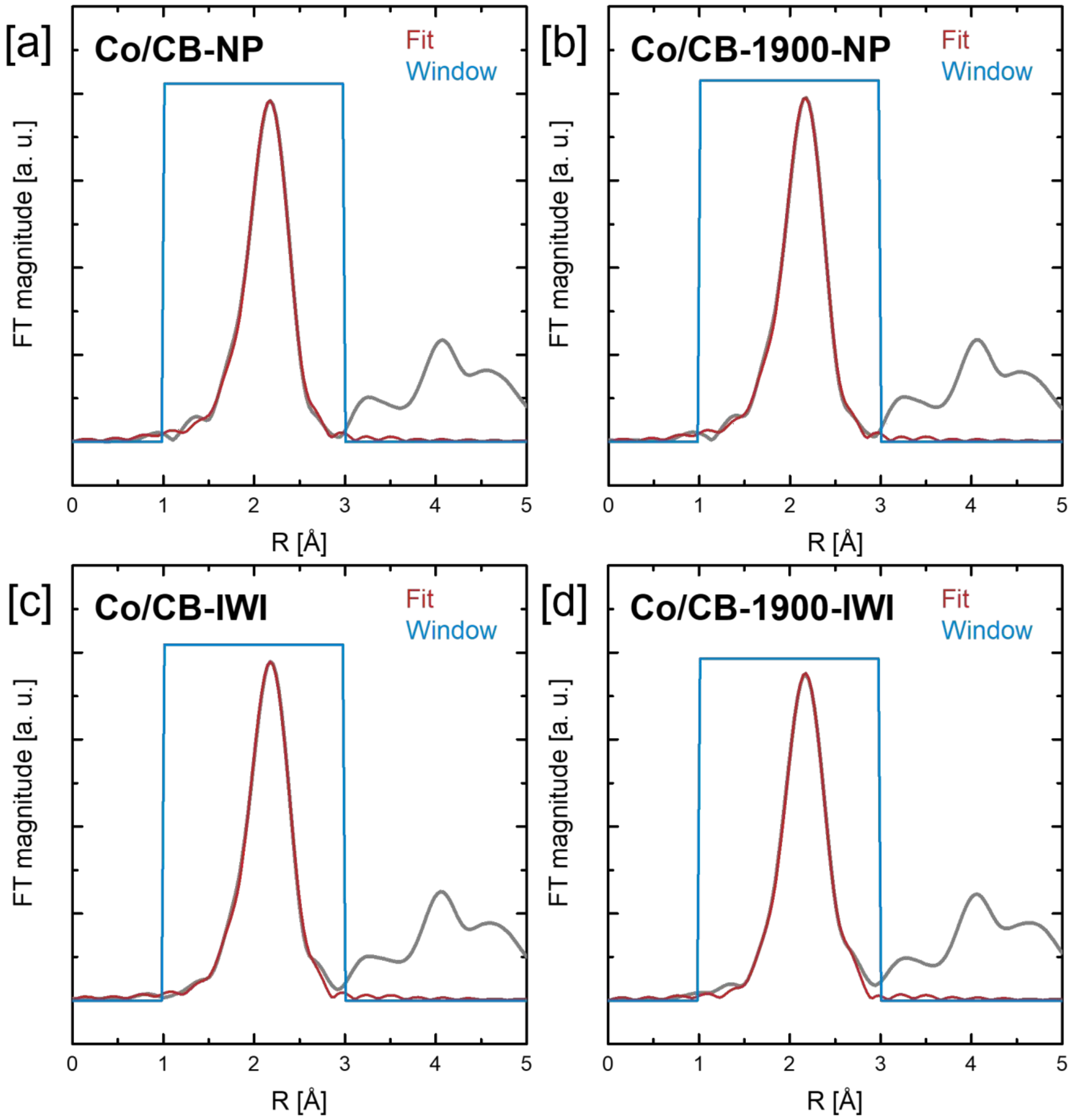

Figure S27. First shell fitting of k^2 weighted in situ EXAFS data in R space of reduced [a] Co/CB-NP, [b] Co/CB-1900-NP, [c] Co/CB-IWI, [d] Co/CB-1900-IWI.

Table S2. First and second shell fitting parameters of in situ EXAFS data measured after catalyst reduction. Please note that for the incipient wetness impregnated samples, fractional coordination numbers were

Sample	Phase	Scattering path	E_0 [eV]	CN	R [Å]	σ^2 [Å]	R-factor
**Co/CB-NP**	Co^0	Co-Co	6.8	10.7	2.49	0.007	0.004
**Co/CB-1900-NP**	Co^0	Co-Co	6.0	10.8	2.49	0.007	0.003
	Co^0	Co-Co		10.8	2.49	0.007	
**Co/CB-IWI**	CoO	Co-O	6.7	0.4	2.06	0.017	0.003
	CoO	Co-Co		0.9	2.91	0.017	
	Co^0	Co-Co		10.0	2.49	0.007	
**Co/CB-1900-IWI**	CoO	Co-O	5.9	0.6	2.09	0.005	0.003
	CoO	Co-Co		1.1	2.96	0.008	

Side note S4: In situ EXAFS analysis

To further investigate the local structure of the cobalt phase in the reduced catalysts, in situ EXAFS measurements were conducted without any exposure to air (Figures S25-27). Analysis of the first coordination shell revealed no significant deviation in bond length compared to a bulk Co hcp reference (2.49 Å), remaining well within the expected measurement and analysis uncertainty of ±0.02 Å (Table S2). Similarly, the extracted first-shell Co^0–Co^0 coordination numbers (CN) were broadly consistent with the bulk reference value of 12, considering the typical ±10 % uncertainty associated with EXAFS coordination number analysis (Table S2). An exception to this trend was observed for Co/CB-IWI, where the measured CN of 10.8 appeared inconsistent with the average particle size determined by STEM (3.6 ± 2 nm, Figure S17), as particle sizes <5 nm are expected to be reflected by decreasing first-shell coordination numbers.[17] This discrepancy is likely the consequence of the presence of a small population of larger cobalt particles, as observed by STEM imaging (Figure S18).

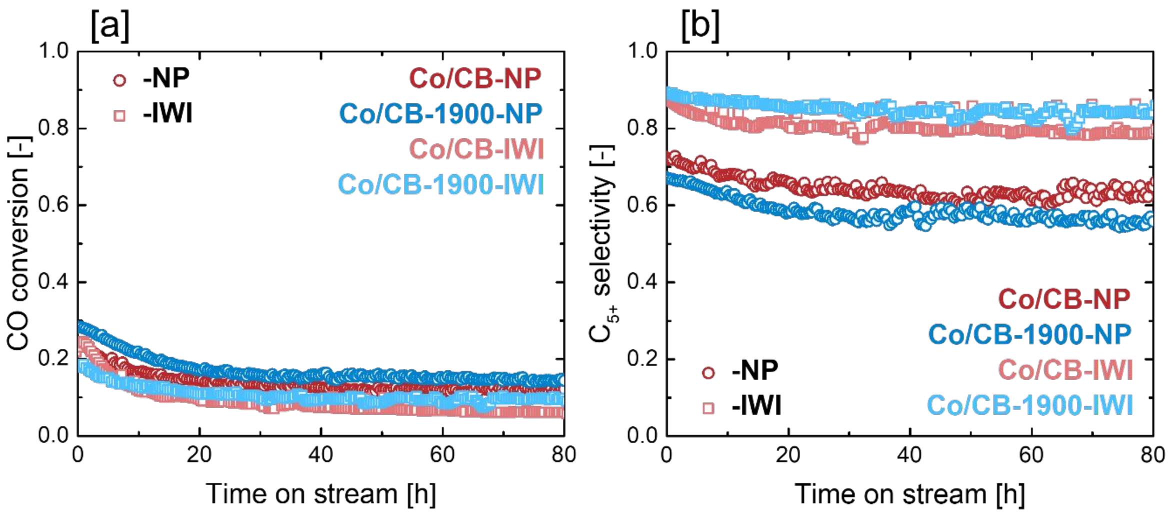

Figure S28. [a] CO conversion and [b] C_{5+} selectivity of Co/C catalysts over 80 h time on stream at 220 °C, 20 bar, H_2:CO 2.1.

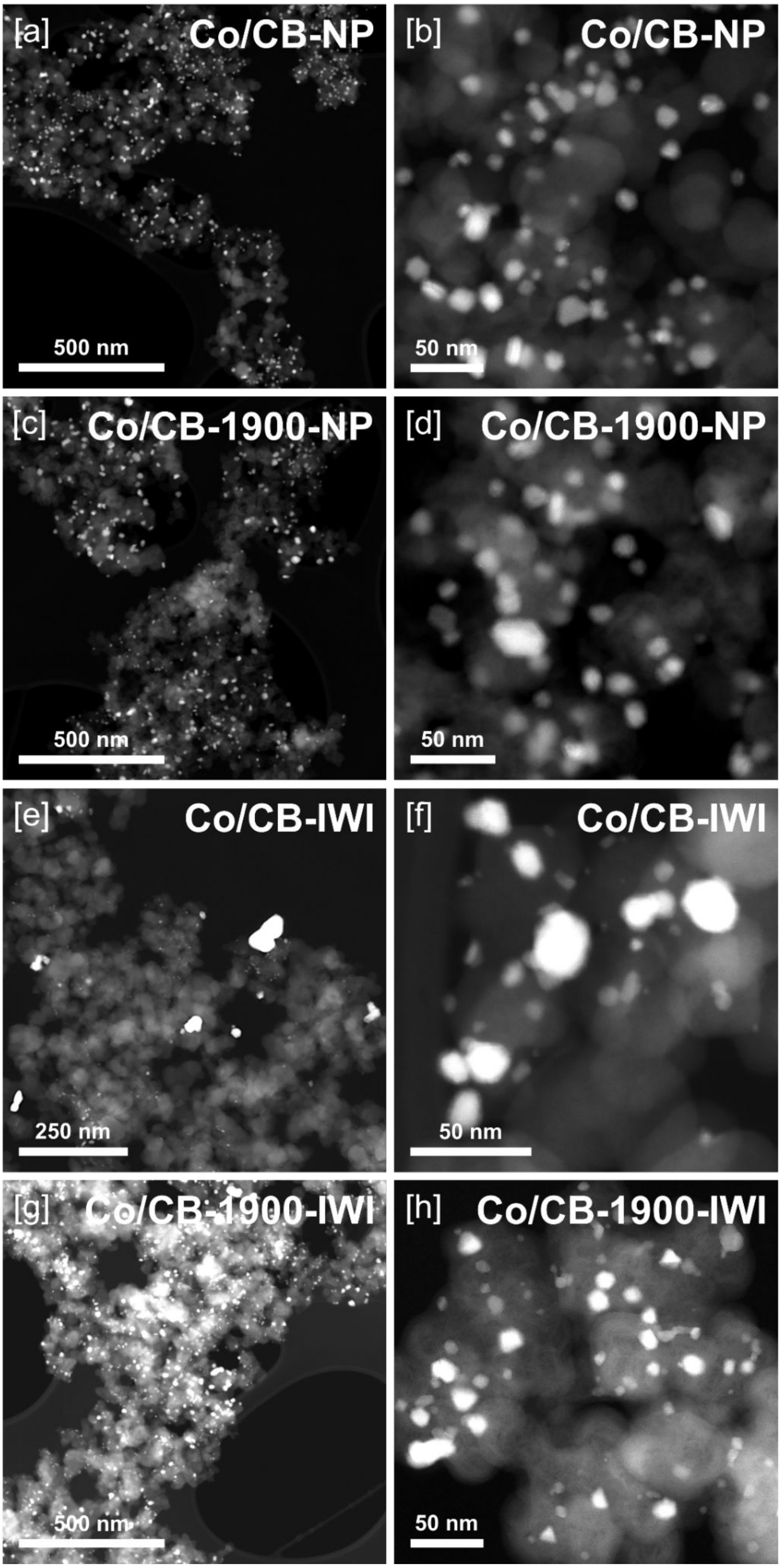

Figure S29. HAADF-STEM micrographs of the Co/C catalysts after 80 h FTS/passivation.

REFERENCES

[1] P. Mallet-Ladeira, P. Puech, C. Toulouse, M. Cazayous, N. Ratel-Ramond, P. Weisbecker, G.L. Vignoles, M. Monthioux, A Raman study to obtain crystallite size of carbon materials, Carbon 80 (2014) 629–639. https://doi.org/10.1016/j.carbon.2014.09.006.

[2] S. Kundu, Y. Wang, W. Xia, M. Muhler, Thermal Stability and Reducibility of Oxygen-Containing Functional Groups on Multiwalled Carbon Nanotube Surfaces, J. Phys. Chem. C 112 (2008) 16869–16878. https://doi.org/10.1021/jp804413a.

[3] B. Lesiak, L. Kövér, J. Tóth, J. Zemek, P. Jiricek, A. Kromka, N. Rangam, C sp2/sp3 hybridisations in carbon nanomaterials – XPS and (X)AES study, Appl. Surf. Sci. 452 (2018) 223–231. https://doi.org/10.1016/j.apsusc.2018.04.269.

[4] T.W. van Deelen, H. Yoshida, R. Oord, J. Zečević, B.M. Weckhuysen, K.P. de Jong, Cobalt nanocrystals on carbon nanotubes in the Fischer-Tropsch synthesis, Appl. Catal., A 593 (2020) 117441. https://doi.org/10.1016/j.apcata.2020.117441.

[5] T. Fu, Y. Jiang, J. Lv, Z. Li, Effect of carbon support on Fischer–Tropsch synthesis activity and product distribution over Co-based catalysts, Fuel Process. Technol. 110 (2013) 141–149. https://doi.org/10.1016/j.fuproc.2012.12.006.

[6] H. Xiong, M.A. Motchelaho, M. Moyo, L.L. Jewell, N.J. Coville, Correlating the preparation and performance of cobalt catalysts supported on carbon nanotubes and carbon spheres in the Fischer–Tropsch synthesis, J. Catal. 278 (2011) 26–40. https://doi.org/10.1016/j.jcat.2010.11.010.

[7] C.-W. Tang, C.-B. Wang, S.-H. Chien, Characterization of cobalt oxides studied by FT-IR, Raman, TPR and TG-MS, Thermochim. Acta 473 (2008) 68–73. https://doi.org/10.1016/j.tca.2008.04.015.

[8] S. Osswald, J. Chmiola, Y. Gogotsi, Structural evolution of carbide-derived carbons upon vacuum annealing, Carbon 50 (2012) 4880–4886. https://doi.org/10.1016/j.carbon.2012.06.016.

[9] I.C. Gerber, P. Serp, A Theory/Experience Description of Support Effects in Carbon-Supported Catalysts, Chem. Rev. 120 (2020) 1250–1349. https://doi.org/10.1021/acs.chemrev.9b00209.

[10] R. Voggu, B. Das, C.S. Rout, C.N.R. Rao, Effects of charge transfer interaction of graphene with electron donor and acceptor molecules examined using Raman spectroscopy and cognate techniques, J. Phys.: Condens. Matter 20 (2008) 472204. https://doi.org/10.1088/0953-8984/20/47/472204.

[11] C. Zhou, J.A. Szpunar, Hydrogen Storage Performance in Pd/Graphene Nanocomposites, ACS Appl. Mater. Interfaces 8 (2016) 25933–25940. https://doi.org/10.1021/acsami.6b07122.

[12] X. Zheng, W. Chen, G. Wang, Y. Yu, S. Qin, J. Fang, F. Wang, X.-A. Zhang, The Raman redshift of graphene impacted by gold nanoparticles, AIP Adv. 5 (2015). https://doi.org/10.1063/1.4921316.

[13] V.B. Parambhath, R. Nagar, K. Sethupathi, S. Ramaprabhu, Investigation of Spillover Mechanism in Palladium Decorated Hydrogen Exfoliated Functionalized Graphene, J. Phys. Chem. C 115 (2011) 15679–15685. https://doi.org/10.1021/jp202797q.

[14] W.A. Sławiński, E. Zacharaki, H. Fjellvåg, A.O. Sjåstad, Structural Arrangement in Close-Packed Cobalt Polytypes, Cryst. Growth Des. 18 (2018) 2316–2325. https://doi.org/10.1021/acs.cgd.7b01736.

[15] N.E. Tsakoumis, E. Patanou, S. Lögdberg, R.E. Johnsen, R. Myrstad, W. van Beek, E. Rytter, E.A. Blekkan, Structure–Performance Relationships on Co-Based Fischer–Tropsch Synthesis Catalysts: The More Defect-Free, the Better, ACS Catal. 9 (2019) 511–520. https://doi.org/10.1021/acscatal.8b03549.

[16] F. Herold, D. de Oliveira, G. Baade, J. Friedland, R. Güttel, M. Claeys, M. Rønning, Is Carbon Heteroatom Doping the Key to Active and Stable Carbon Supported Cobalt Fischer–Tropsch Catalysts?, ACS Catal. 15 (2025) 6673–6689. https://doi.org/10.1021/acscatal.4c08092.

[17] A.M. Beale, B.M. Weckhuysen, EXAFS as a tool to interrogate the size and shape of mono and bimetallic catalyst nanoparticles, Phys. Chem. Chem. Phys. 12 (2010) 5562–5574. https://doi.org/10.1039/B925206A.
